# 29 m^6^A-RNA Methylation (Epitranscriptomic) Regulators Are Regulated in 41 Diseases including Atherosclerosis and Tumors Potentially via ROS Regulation – 102 Transcriptomic Dataset Analyses

**DOI:** 10.1155/2022/1433323

**Published:** 2022-02-15

**Authors:** Ming Liu, Keman Xu, Fatma Saaoud, Ying Shao, Ruijing Zhang, Yifan Lu, Yu Sun, Charles Drummer, Li Li, Sheng Wu, Satya P. Kunapuli, Gerard J. Criner, Jianxin Sun, Huimin Shan, Xiaohua Jiang, Hong Wang, Xiaofeng Yang

**Affiliations:** ^1^Cardiovascular Research Center, Lewis Katz School of Medicine at Temple University, Philadelphia, PA 19140, USA; ^2^Department of Cell Biology and Genetics, School of Basic Medical Science, Shanxi Medical University, Taiyuan, Shanxi 030001, China; ^3^Department of Nephrology, The Affiliated People's Hospital of Shanxi Medical University, Taiyuan, Shanxi 030001, China; ^4^Metabolic Disease Research; Inflammation, Translational & Clinical Lung Research, Lewis Katz School of Medicine at Temple University, Philadelphia, PA 19140, USA; ^5^Thrombosis Research, Lewis Katz School of Medicine at Temple University, Philadelphia, PA 19140, USA; ^6^Thoracic Medicine and Surgery, Lewis Katz School of Medicine at Temple University, Philadelphia, PA 19140, USA; ^7^Center for Translational Medicine, Department of Medicine, Thomas Jefferson University, Philadelphia, PA 19107, USA

## Abstract

We performed a database mining on 102 transcriptomic datasets for the expressions of 29 m^6^A-RNA methylation (epitranscriptomic) regulators (m^6^A-RMRs) in 41 diseases and cancers and made significant findings: (1) a few m^6^A-RMRs were upregulated; and most m^6^A-RMRs were downregulated in sepsis, acute respiratory distress syndrome, shock, and trauma; (2) half of 29 m^6^A-RMRs were downregulated in atherosclerosis; (3) inflammatory bowel disease and rheumatoid arthritis modulated m^6^A-RMRs more than lupus and psoriasis; (4) some organ failures shared eight upregulated m^6^A-RMRs; end-stage renal failure (ESRF) downregulated 85% of m^6^A-RMRs; (5) Middle-East respiratory syndrome coronavirus infections modulated m^6^A-RMRs the most among viral infections; (6) proinflammatory oxPAPC modulated m^6^A-RMRs more than DAMP stimulation including LPS and oxLDL; (7) upregulated m^6^A-RMRs were more than downregulated m^6^A-RMRs in cancer types; five types of cancers upregulated ≥10 m^6^A-RMRs; (8) proinflammatory M1 macrophages upregulated seven m^6^A-RMRs; (9) 86% of m^6^A-RMRs were differentially expressed in the six clusters of CD4^+^Foxp3^+^ immunosuppressive Treg, and 8 out of 12 Treg signatures regulated m^6^A-RMRs; (10) immune checkpoint receptors TIM3, TIGIT, PD-L2, and CTLA4 modulated m^6^A-RMRs, and inhibition of CD40 upregulated m^6^A-RMRs; (11) cytokines and interferons modulated m^6^A-RMRs; (12) NF-*κ*B and JAK/STAT pathways upregulated more than downregulated m^6^A-RMRs whereas TP53, PTEN, and APC did the opposite; (13) methionine-homocysteine-methyl cycle enzyme Mthfd1 downregulated more than upregulated m^6^A-RMRs; (14) m^6^A writer RBM15 and one m^6^A eraser FTO, H3K4 methyltransferase MLL1, and DNA methyltransferase, DNMT1, regulated m^6^A-RMRs; and (15) 40 out of 165 ROS regulators were modulated by m^6^A eraser FTO and two m^6^A writers METTL3 and WTAP. Our findings shed new light on the functions of upregulated m^6^A-RMRs in 41 diseases and cancers, nine cellular and molecular mechanisms, novel therapeutic targets for inflammatory disorders, metabolic cardiovascular diseases, autoimmune diseases, organ failures, and cancers.

## 1. Introduction

Cardiovascular diseases (CVDs) including coronary heart disease, cerebrovascular disease, and peripheral artery disease have risen to the top of the worldwide death toll [1, 2]. Previous studies showed that different risk factors including elevated plasma lipids [3, 4], elevated blood sugar [5], hyperhomocysteinemia [6, 7], and chronic kidney disease [8–10] promote vascular inflammation and atherosclerotic CVDs by different mechanisms including DNA methylation [11], microRNA regulation of mRNA stabilities [12], histone modifications including histone methylations [13–15], immune metabolic programming and trained immunity [16], innate immune activation [17] of endothelial cells (EC) [18–21], EC injury [22], Ly6C high mouse monocyte and CD40^+^ human monocyte differentiation [23, 24], decreased regulatory T cells (Treg) [25–27], and impaired bone marrow-derived progenitor cells' vascular repairability [28, 29]. Furthermore, we recently proposed new models that include intracellular organelle dangers [30] and reactive oxygen species (ROS) as an integrated sensing system for metabolic homeostasis and alarming, which showed that metabolic remodeling and dysfunction trigger mitochondrial ROS [31–34], caspase-1/inflammasome activation [8, 10], histone modification enzyme downregulation [14], and increased expressions of trained immunity pathway enzymes. These reports have demonstrated that epigenomic mechanisms play significant roles in connecting metabolic reprogramming and dysfunction to inflammation initiation and gene transcription. However, the mechanism by which epitranscriptomic-RNA methylation controls the progression of many diseases is still unknown.

RNA carries a spectrum of more than 100 chemical modifications including RNA methylation that play significant roles in the regulation of gene expression [35, 36]. As the most dominant mRNA modification, N^6^-methyladenosine (m^6^A), installed onto mRNA by the methyltransferase-like 3 (METTL3)/methyltransferase-like 14 (METTL14) methyltransferase complex, is at a frequency of 0.15-0.6% of all adenosines in polyadenylated RNA [37]. In addition, six other RNA methylations have also been reported such as pseudouridine (*Ψ*), 5-methylcytidine (m^5^C), N1-methyladenosine (m^1^A), N4-acetylcytidine (ac^4^C), ribose methylations (Nm), and N7-methylguanosine (m^7^G) [38]. At 25-60% of transcriptome, m^6^A methylation regulates gene expression by influencing numerous aspects of mRNA processes of RNA polymerase II-transcribed mRNAs such as pre-mRNA processing, nuclear export, decay, and translation as well as long noncoding RNAs (lncRNAs) [39]. In addition, m^6^A plays an important role on noncoding chromosome-associated regulatory RNAs (carRNAs) for gene expression, which includes enhancer RNAs (eRNAs), promoter-associated RNAs (paRNAs), and transposable element transcribed RNA (repeat RNAs). Similar to DNA methylation as a mode of epigenomic regulation, the m^6^A methylation that occurred in RNA as a mode of epitranscriptomic regulation becomes important in the crucial roles of m^6^A-mediated gene regulation in many physiological and disease processes [37]. Another important feature of m^6^A methylation is the reversibility, allowing for the regulation of m^6^A levels after initial deposition. Fat mass and obesity-associated protein (FTO) and AlkB Homolog 5 (ALKBH5) have been discovered as m^6^A demethylases (erasers).

The m^6^A methylation also plays a significant role in human disease pathology. Loss of METTL14 has been shown to increase endometrial cancer cells' proliferation and tumorigenicity. In contrast, METTL3/METTL14 have been found to play significant roles in acting as oncogenes in acute myeloid leukemia and glioblastoma and promoting or inhibiting roles in hepatocellular carcinoma [40]. Since METTL3/METTL14 have been indicated to be promising drug targets, phase 1 trials of small-molecule inhibitors for METTL3/METTL14 have been planned for 2021-2022 [37]. In addition, METTL3/METTL14 and m^6^A methylation have been reported to play roles in other diseases such as heart failure, viral infection, type 2 diabetes [37], cardiac remodeling, atherosclerosis, congenital heart disease, inflammation, obesity, insulin resistance, adipogenesis, and hypertension [41]. Moreover, decreased expressions of fat mass and obesity-associated protein (FTO) [42] have been related to heart failure, and overexpression of FTO in failing heart results in decrease of ischemia-triggered loss of cardiac function. Finally, YT521-B homology (YTH, m^6^A-dependent RNA binding) domain family (YTHDF) [43] reader proteins are also involved in pathogenic processes since YTHDF2 is essential for acute myeloid leukemia initiation and leukemia stem cell development [37].

**Figure 1:**
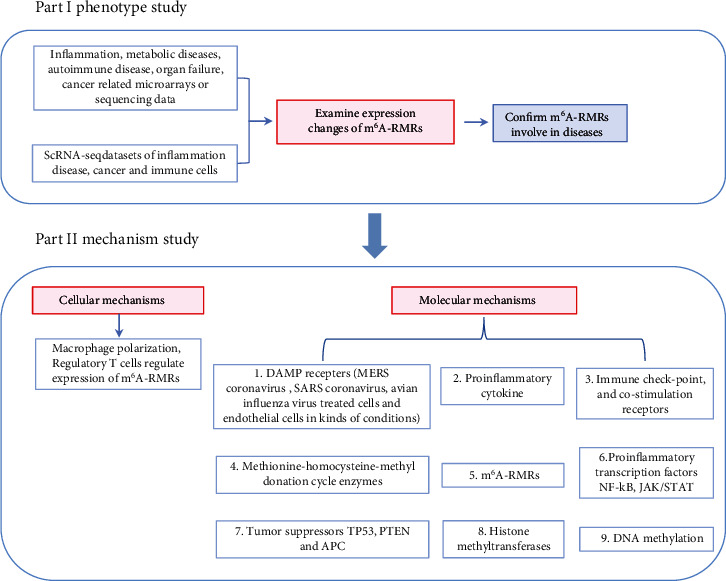
Flow chart of the study. Data mining work includes two parts: (I) the expression changes of m^6^A-RMRs in diseases including acute inflammation, sepsis, acute respiratory distress syndrome, shock, trauma, cardiovascular diseases (CVDs), autoimmune diseases, organ failures, and cancers were examined; (II) cellular mechanisms including macrophages and CD4^+^Foxp3^+^ regulatory T cell (Treg) modulation and molecular mechanisms including the role of danger-associated molecular pattern receptors (DAMP receptors); proinflammatory cytokines; immune checkpoint and costimulation receptors; methionine-homocysteine-methyl donation cycle enzymes; m^6^A-RMRs; proinflammatory transcription factors NF-*κ*B and JAK/STAT; tumor suppressors TP53, PTEN, and APC; histone methyltransferases; and DNA methyltransferases in regulation of m^6^A-RMRs were explored.

Despite major advancements in the discipline, the following questions remain unanswered: (1) whether the expressions of 29 m^6^A-RMRs are modulated in 41 diseases and cancers including acute inflammation, sepsis, acute respiratory distress syndrome, shock, trauma, cardiovascular diseases (CVDs), autoimmune diseases, and organ failures; (2) whether macrophages and CD4^+^Foxp3^+^ regulatory T cells (Treg) serve as the key cellular mechanisms underlying the roles of m^6^A-RMRs in various diseases and cancers; and (3) whether nine types of molecular mechanisms including danger-associated molecular pattern receptors (DAMP receptors); proinflammatory cytokines; immune checkpoint and costimulation receptors; methionine-homocysteine-methyl donation cycle enzymes; m^6^A-RMRs; proinflammatory transcription factors NF-*κ*B and JAK/STAT; tumor suppressors TP53, PTEN, and APC; histone methyltransferases; and DNA methyltransferases play significant roles in modulating m^6^A-RMRs. After analyzing 102 transcriptomic datasets according to our flow chart (Figure 1), we made significant findings as summarized in Abstract. Our findings reveal new information about the roles of elevated m^6^A-RMRs in the pathogenesis of 41 illnesses and tumors, as well as new therapeutic targets for inflammation, sepsis, trauma, organ failures, autoimmunity, metabolic cardiovascular disorders, and cancers.

## 2. Materials and Methods

### 2.1. Expression Levels of m^6^A-RMRs in Patients with Various Inflammatory Disorders and Tumors

We collected 23 microarray datasets of acute inflammation, metabolic and cardiovascular diseases, autoimmune diseases, and organ failures (Table 1); one microarray of Middle-East respiratory syndrome (MERS) coronavirus-infected human microvascular endothelial cells; one microarray dataset (nine comparisons) of subacute respiratory syndrome coronavirus- (SARS-CoV-) infected human airway epithelial cells; one microarray dataset (23 comparisons) of influenza virus-infected lung epithelial cells (Table 2); and eight microarray datasets of endothelial cells (Table 3) from National Institutes of Health- (NIH-) National Center for Biotechnology Information- (NCBI-) Gene Expression Omnibus (GEO) databases (https://www.ncbi.nlm.nih.gov/gds/). These datasets were analyzed with GEO2R (https://www.ncbi.nlm.nih.gov/geo/geo2r/). Some datasets were overlapped with our previous studies [44]. In addition, the Oncomine database (https://www.oncomine.org) was used to analyze the gene expression profile from 19 tumors [45], with threshold parameters of fold change > 2, *p* < 0.05, and gene rank in the top 10%. Because these microarray studies employed diverse cell types, we were unable to compare the effects of illness circumstances on m^6^A-RMR regulation in the same cell types. It is worth noting that our strategy was well justified. For example, we and others frequently investigated gene expression in nonideal heterogeneous peripheral blood mononuclear cell populations (PBMCs) in pathophysiological conditions, which are made up of a variety of cell types (also see Discussion).

### 2.2. Expression Profile of m^6^A-RMRs in Single-Cell RNA Sequencing (scRNA-Seq) Datasets from Studies of Sepsis, Atherosclerosis, Tumors, and Endothelial Cell

Five scRNA-Seq datasets were collected from the Single Cell Portal database (https://singlecell.broadinstitute.org/single_cell), including one study about sepsis, one study about atherosclerosis, two studies about tumors (astrocytoma and melanoma), and one study about endothelial cell populations (Supplementary Table 1). The expressions of 29 m^6^A-RMRs were online analyzed.

### 2.3. Expression Regulation Analysis of m^6^A-RMRs from Deficiency of Folate Cycle and Metabolism-Related Enzymes, m^6^A-RMRs, H3K4 Methylase, DNA Methyltransferase, Regulatory T Cells' Signature Genes, Proinflammatory Cytokines, Oncogene, Tumor Suppressors, and Immune Checkpoint Receptors

The 68 microarray datasets in the NIH-NCBI-GeoDataset database (https://www.ncbi.nlm.nih.gov/gds/) were collected in analyzing the regulatory mechanisms of m^6^A-RMRs (Supplementary Table 2). There are six microarrays about deficiencies of the folate cycle and metabolism-related enzymes and m^6^A-RMRs, four datasets of deficiencies of H3K4 methylase, six datasets of deficiencies of DNA methyltransferase, three datasets of Treg versus conventional T cells, 11 datasets of deficiencies of Treg cells' signature genes, one dataset of macrophage polarization, eight datasets of deficiencies of proinflammatory cytokines, nine datasets of deficiencies of oncogene, ten datasets of deficiencies of tumor suppressors, and ten datasets of deficiencies of immune checkpoint receptors.

### 2.4. Statistical Analysis of Microarray Data

The expression changes of m^6^A-RMRs are not big enough in diseases; we may miss some important information if the expression change fold thresholds are set high. Thus, the expression changes were listed in the results with *p* < 0.05 (statistical significance), m^6^A-RMRs with expression changes more than 1-fold (red) were defined as the upregulated genes, while genes with expression decreased more than 1-fold (blue) were defined as downregulated genes. The missing value was marked with “NA,” and “NA” was excluded in the total count number.

## 3. Results

### 3.1. A Few m^6^A-RNA Methylation Regulators (m^6^A-RMRs) Are Upregulated, and Most m^6^A-RMRs Are Downregulated in Sepsis, Sepsis plus Acute Respiratory Distress Syndrome, Sepsis plus Shock, and Trauma, and Upregulated Two RNA Methyltransferases WTAP and PCIF1 and Three RNA Binding Proteins YTHDF3, IGF2BP2, and IGF2BP3 May Promote Acute Inflammations

**Figure 2:**
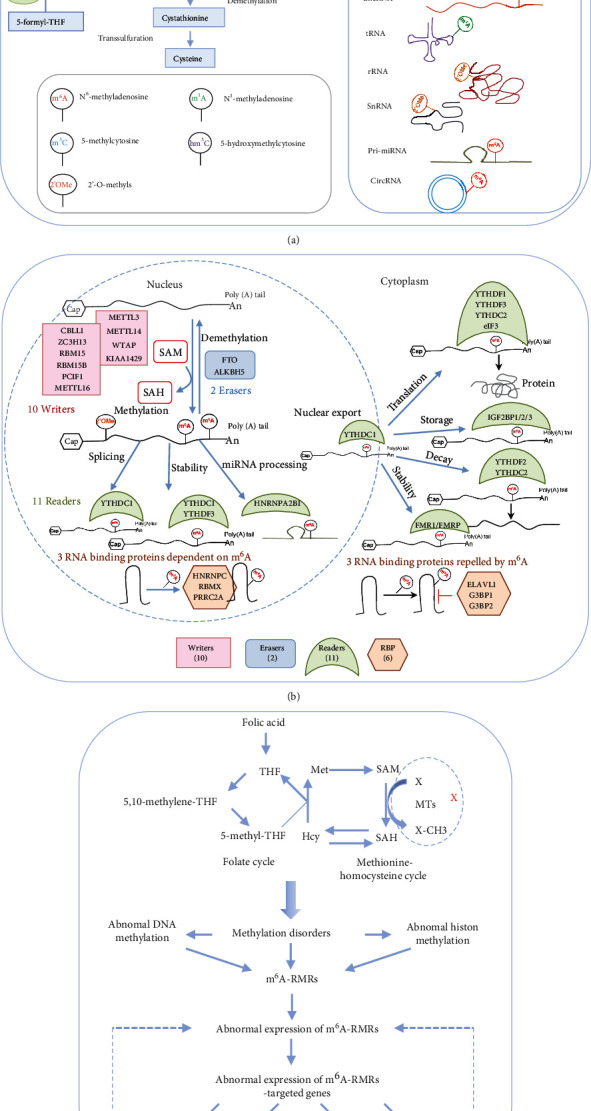
An overview of RNA methylation processes, which include (a) homocysteine-methionine-folate cycles for methyl generation and donation; (b) 29 m^6^A-RNA methylation regulators (m^6^A-RMRs) are classified into five groups: (i) RNA methylation writers, (ii) RNA methylation erasers, (iii) RNA methylation readers, (iv) m^6^A-dependent RNA binding proteins (RBPs), and (v) m^6^A repelled RNAs; (c) the hypothesis we proposed. (a) The methyl group is transferred to proteins, nucleic acids, and other biochemicals from the biochemical reaction of S-adenosylmethione (SAM) to S-adenosylhomocysteine (SAH) in homocysteine-methionine metabolic cycle. For RNA methylation, mRNA, lncRNA, tRNA, SnRNA, pre-miRNA, and CirRNA can be methylated in many different ways. In addition to the basic methylated forms, the common methylated forms include N6-methyladenosine (m^6^A), N1-methyladenosine, N1-methyladenosine, 2′-O-methyls at mRNA, N6-methyladenosine at lncRNA, pre-miRNA, circRNA [50–55], N1-methyladenosine at tRNA, 2′-O-methyls at rRNA, and snRNA [56]. (b) The reported 29 m^6^A-RMRs were chosen for analysis, including 10 m^6^A methyltransferase enzymes (writers), two m6A demethylases (erasers), 11 m^6^A binding proteins (readers), three m^6^A dependent on RNA binding proteins, and three RNA binding proteins repelled by m^6^A. The detailed information including the reference articles of the 29 m^6^A-RMRs is listed in Supplementary Table 3. (c) The methyl donor is derived from folate cycle and coupled homocysteine-methionine cycle. When the donor source is impaired, the DNA, RNA, and protein methylations will be in disorder. We proposed the hypothesis: mRNA methylation disorders will affect the expressions of m^6^A-RMRs; then, the m^6^A-RMRs will be involved in inflammation, metabolic diseases, and tumors by methylating or demethylating target genes including RNAs.

**Figure 3:**
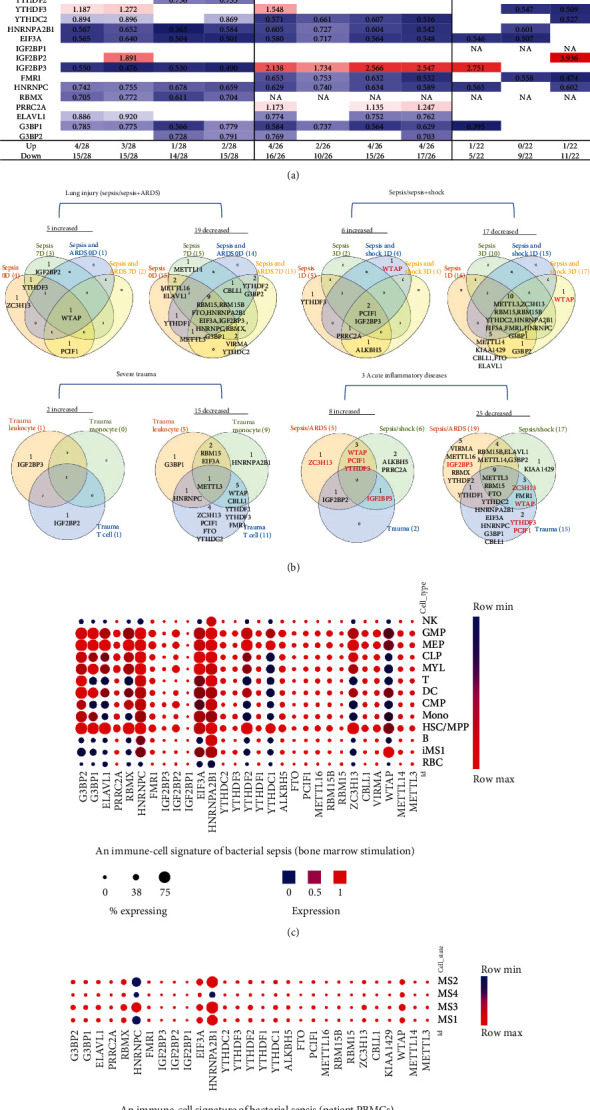
Most m^6^A-RNA methylation regulators (m^6^A-RMRs) were downregulated in acute inflammatory diseases, suggesting that most m^6^A-RMRs are not required for the pathogenesis of acute inflammatory diseases. (a) The results showed that m^6^A-RMRs were downregulated (marked in blue) in sepsis and acute respiratory distress syndrome (ARDS), sepsis and shock, and severe trauma. (b) Venn diagram showed that in the same base disease, there were the shared upregulated or downregulated m^6^A regulators. There were five upregulated m^6^A-RMRs including shared writers WTAP and 19 downregulated regulators including nine shared genes in sepsis and ARDS of lung injury. There were six upregulated regulators including common writer PCIF1 and reader IGF2BP3 and 17 downregulated m^6^A methylation regulators (10 common genes) in sepsis and combined shock. WTAP was upregulated on the first day of sepsis and shock. In severe trauma, there were two genes upregulated and 15 genes downregulated including one common writer METTL3. In the three acute inflammatory diseases, nine downregulated m6A regulators (METTL3, RBM15, FTO, YTHDC2, HNRNPA2B1, EIF3A, HNRNPC, G3BP1, and CBLL1) were shared by three acute inflammation diseases. Note: the red marked genes are those that are up- or downregulated in different diseases. (c) 29 m^6^A-RMRs were examined in single-cell sequencing dataset online (https://singlecell.broadinstitute.org/single_cell) in an immune-cell signature of bacterial sepsis study (PMID: 32066974). The 11 out of 29 m^6^A methylation regulators including WTAP, ZC3H13, YTHDC1, YTHDF2, HNRNPA2B1, EIF3A, HNRNPC, RBMX, ELAVL1, G3BP1, and G3BP2 expressed differentially in different cell populations of bone-marrow under stimulation. (d) The expressions of most m^6^A methylation regulators except HNRNPA2B1 and HNRNPC were lower than those of others in PBMC subset cells (*p* < 0.05). Abbreviations: ARDS: acute respiratory distress syndrome; FC: fold change; NA: not available (missing value); NK: natural killer cell; GMP: granulocyte-macrophage progenitor; MEP: megakaryocyte-erythroid progenitors; MYL: myeloblasts; DC: dendritic cell; CMP: common myeloid progenitors; HSC/MPP: hematopoietic stem cells and multipotent progenitors; RBC: red blood cells; MS1: CD14^+^ cells with high expression of resistin (RETN), arachidonate 5-lipoxygenase activating protein (ALOX5AP), and interleukin-1 receptor type 2 (IL1R2); MS2: with high expression of class II major histocompatibility complex (MHC); MS3: similar to nonclassical CD16hi monocytes; MS4: which is composed of the remaining CD14^+^ cells that express low levels of both class II MHC and inflammatory cytokines; NHP: nonhuman primate; mTB: Mycobacterium tuberculosis; SHIV: simian-human immunodeficiency virus; CAF: cancer-associated fibroblasts.

We hypothesized that pathological conditions significantly modulate the expressions of m^6^A-RMRs in cell type-specific and disease-specific manners. To test this hypothesis, we collected 29 m^6^A-RMRs in five groups from PubMed database (https://pubmed.ncbi.nlm.nih.gov/) (Figure 2 and Supplementary Table 3). The 29 m^6^A-RMRs included (1) 10 m^6^A-RNA methylation writers (methyltransferases) including METTL14, METTL3, WTAP, KIAA1429, ZC3H13,CBLL1, RBM15, METTL16,RBM15B, and PCIF1; (2) two m^6^A-RNA demethylases, FTO and ALKBH5; (3) 11 RNA binding proteins (readers) including YTHDC1, YTHDF1, YTHDF2, YTHDF3, YTHDC2, HNRNPA2B1, EIF3A, IGF2BP1, IGF2BP2, IGF2BP3, and FMR1; (4) three m^6^A-dependent RNA binding proteins including HNRNPC, RMMX, and PRRC2A; and (5) three m^6^A-repelled RNA binding proteins including ELAVL1, G3BP1, and G3BP2. As shown in Figure 3(a), one to four out of 28 m^6^A-RMRs were upregulated; and 14 to 15 out of 28 m^6^A-RMRs were downregulated in 0 day and 7 days in patients with sepsis and sepsis plus acute respiratory distress syndrome (ARDS) [46], respectively. In the second datasets with sepsis and sepsis plus shock, two to four out of 26 m^6^A-RMRs were upregulated; and 10 to 17 out of 26 m^6^A-RMRs were downregulated in 1 day and 3 days in patients with sepsis and sepsis plus shock [47, 48], respectively. In the third datasets with leukocytes, monocytes, and T cells from trauma, zero to one out of 22 m^6^A-RMRs was upregulated; and 5 to 11 out of 22 m^6^A-RMRs were downregulated in leukocytes, monocytes, and T cells from trauma [49], respectively.

We then analyzed the shared and disease-specific m^6^A-RMRs using Venn diagram analysis. As shown in Figure 3(b), four diseases including sepsis at 0 day and 7 days and sepsis plus ARDS at 0 day and 7 days shared one RNA methyltransferase WTAP. In the second sepsis datasets, four diseases including sepsis at 1 day and 3 days and sepsis plus shock at 0 day and 7 days shared one RNA methyltransferase PCIF1 and one RNA methylation reader IGF2BP3. In the trauma datasets, three immune cell types, leukocytes, monocytes, and T cells did not share any upregulated m^6^A-RMRs. When comparing three groups of acute inflammations, sepsis plus ARDS shared three upregulated m^6^A-RMRs such as writers WTAP, PCIF1, and reader YTHDF3 with sepsis plus shock; sepsis plus ARDS shared upregulation of one RNA binding protein IGF2BP2 with trauma, and sepsis plus shock shared one RNA binding protein IGF2BP3 with trauma.

We noticed that in the trauma datasets (Figure 3(b)), three immune cell types, leukocytes, monocytes, and T cells did not share any upregulated RNA methylation regulators, suggesting that upregulation of m^6^A-RMRs is in a cell-specific manner. To further examine this issue, we collected single-cell RNA-sequencing (scRNA-Seq) datasets in sepsis [57] in a comprehensive single-cell sequencing database (https://singlecell.broadinstitute.org/single_cell). As shown in Figure 3(c), the 11 out of 29 m^6^A-RMRs (38%) including WTAP, ZC3H13, YTHDC1, YTHDF2, HNRNPA2B1, EIF3A, HNRNPC, RBMX, ELAVL1, G3BP1, and G3BP2 were expressed differentially in 13 different immune cell types (natural killer cell (NK), granulomyeloid progenitor (GMP), megakaryocyte-erythrocyte progenitor (MEP), common lymphoid progenitor (CLP), myeloblasts (MYL), T cells, dendritic cell (DC), common myeloid progenitor (CMP), monocyte (mono), hematopoietic stem cell/multipotent progenitor (HSC/MPP), B cells, CD14^+^RETN^+^ALOX5AP^+^IL1R2^+^ monocyte (iMS1), and red blood cells (RBC)) in bacterial sepsis. The expressions of most m^6^A-RMRs except HNRNPA2B1 and HNRNPC were lower in monocyte subsets than those in other peripheral mononuclear cell (PBMC) types (Figure 3(d)).

These results have demonstrated that first, a few m^6^A-RMRs are upregulated and most m^6^A-RMRs are downregulated in sepsis, sepsis plus acute respiratory distress syndrome, sepsis plus shock, and trauma; second, two RNA methyltransferases WTAP and PCIF1 and three RNA binding proteins such as YTHDF3, IGF2BP2, and IGF2BP3 are commonly upregulated in sepsis, sepsis plus ARDS, sepsis plus shock, and trauma, suggesting that these five m^6^A-RMRs are the emergency m^6^A-RMRs for promoting acute inflammatory diseases; third, nine m^6^A-RMRs including METTL3, RBM15, FTO, YTHDC2, HNRNPA2B1, EIF3A, HNRNPC, G3BP1, and CBLL1 are commonly downregulated in sepsis, sepsis plus ARDS, sepsis plus shock, and trauma, suggesting that those m^6^A-RMRs play more important roles in maintaining homeostasis and suppressing inflammation than emergency roles for acute inflammations; and fourth, the expressions of 38% m^6^A-RMRs in immune cell types in response to sepsis stimulation are different.

### 3.2. Type 2 Diabetes Has More Modulation of m^6^A-RMR Expressions Than Atherogenic Diseases and Obesity; Nearly Half of 29 m^6^A-RMRs Are Downregulated as Atherosclerosis Progression Compared with That of Atherosclerosis Regression

**Figure 4:**
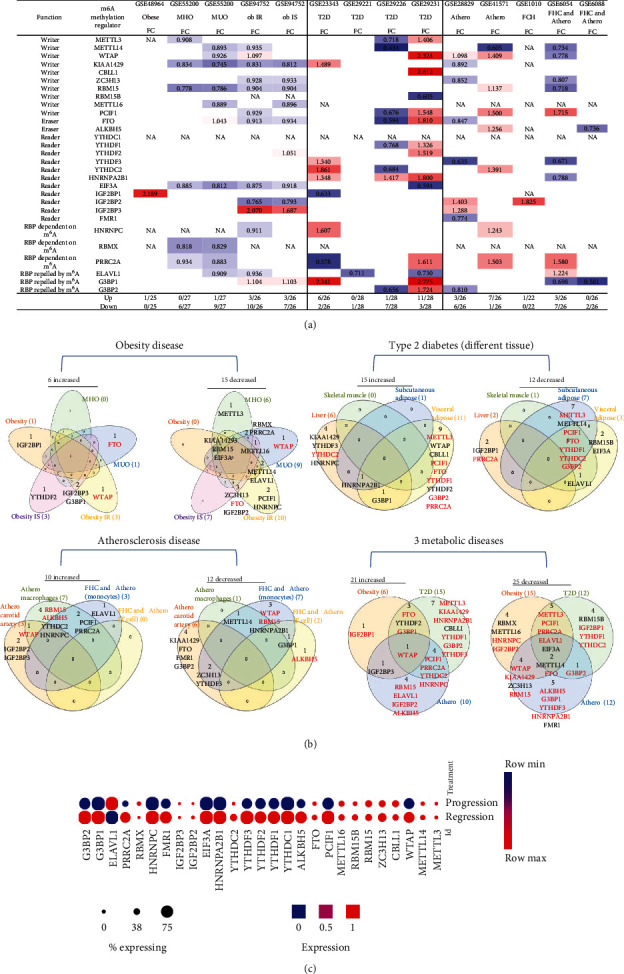
More m^6^A-RNA methylation regulators (m^6^A-RMRs) upregulated in type 2 diabetes and abnormal lipid metabolism than in obesity and obesity-related diseases. (a) The result showed that the majority m^6^A-RMRs were downregulated (marked in blue) in obesity and obesity-related diseases, and only the expression fold change of IGF2BP1 and IGF2BP3 are above two. The expression pattern of m^6^A-RMRs in different tissues of T2D and atherosclerosis disease is different. (b) Venn diagram showed that in obesity, the decreased genes are more than the increased genes (six and 15, respectively); in T2D, few genes are shared in different tissues; in atherosclerosis disease, PCIF and PRRC2A are commonly upregulated and METTL14 is commonly downregulated in macrophages and monocytes. In the three metabolic diseases, the majority m^6^A-RMRs showed the heterogeneity expression (genes marked in red). METTL14 is the common decreased m^6^A methylation regulator among obesity, T2D, and atherosclerosis diseases. Note: the red marked genes are those that are up- or downregulated in different diseases. Expression of m^6^A-RMRs is different in cell populations of atherosclerosis study. 29 RNA m^6^A-RMRs were examined in single-cell sequencing dataset online (https://singlecell.broadinstitute.org/single_cell) in atherosclerotic mice (PMID: 30830865). The results showed that expressions of Wtap, Pcif1, Alkbh5, Ythdc1, Ythdf1, Ythdf2, Ythdf3, Hnrnpa2b1, Eif3a, Fmr1, Hnrnpc, Prrc2a, G3bp1, and G3bp2 were decreased in progressive atherosclerosis compared with regression atherosclerosis. The expression of Elavl1 was increased in progressive atherosclerosis compared with regression atherosclerosis (Virma, Igf2bp1 not found) (*p* < 0.05). Abbreviations: FC: fold change; T2D: type 2 diabetes; MUO: metabolically unhealthy obese; FCH: familial combined hyperlipidemia; FHC: familial hypercholesterolemia; NA: not available (missing value).

We hypothesized that major metabolic cardiovascular disease groups such as obesity [58, 59], type 2 diabetes, and atherogenic diseases differentially modulate the expressions of m^6^A-RMRs. To test this hypothesis, we collected five datasets of obesity including obese, metabolically unhealthy obesity (MUO), metabolically healthy obesity (MHO), obese with insulin resistance (ob IR), and obese with insulin sensitivity (ob IS); four datasets of type 2 diabetes; and five datasets of atherosclerosis, familial hypercholesterolemia (FHC) plus atherosclerosis, and familial combined hyperlipidemia (FCH) from the NCBI-GeoDataset (https://www.ncbi.nlm.nih.gov/gds/). As shown in Figure 4(a), in five diseases in the obesity group, zero to three m^6^A-RMRs were upregulated and zero to 10 m^6^A-RMRs were downregulated. However, in the four type 2 diabetes datasets, zero to 11 m^6^A-RMRs were upregulated and one to seven m^6^A-RMRs were downregulated. The five datasets of atherosclerotic diseases had modulations of m^6^A-RMRs higher than those in the obesity group but lower than those of type 2 diabetes group: zero to seven m^6^A-RMRs were upregulated, and zero to seven m^6^A-RMRs were downregulated.

We also analyzed the shared and disease-specific m^6^A-RMRs using Venn diagram analysis (Figure 4(b)). In six m^6^A-RMRs upregulated in the obesity group, two regulators IGF2BP3 (RNA methyltransferase) and G3BP1 (m^6^A repelled RNA binding protein) were shared by obesity IS and obesity IR. Among the 15 m^6^A-RMRs upregulated in four different tissues in type 2 diabetes, one regulator HNRNPA2B1 (reader) was shared by liver, subcutaneous adipose, and visceral adipose, and one regulator G3BP1 was shared by liver and visceral adipose. In 10 m^6^A-RMRs upregulated in atherosclerotic diseases, one regulator WTAP (RNA methyltransferase) was shared by atherosclerosis (athero) carotid artery and atheromacrophages, and two regulators PCIF1 (RNA methyltransferase) and PRRC2A (m^6^A dependent RNA binding protein) were shared by atheromacrophages and FHC and atheromonocytes. Among 21 m^6^A-RMRs upregulated in three major metabolic cardiovascular disease groups, one regulator WTAP (RNA methyltransferase) was shared by three major groups; one regulator IGF2BP3 was shared by obesity and athero; three regulators FTO (demethylase), YTHDF2 (RNA binding protein), and G3BP1 were shared by obesity and type 2 diabetes; and four regulators such as PCIF1 (RNA methyltransferase), PRRC2A (m^6^A-dependent RNA binding protein), YTHDC2 (RNA binding protein), and HNRNPC (m^6^A-dependent RNA binding protein) were shared by type 2 diabetes and atherosclerotic diseases.

We then examined a hypothesis that atherosclerosis progression and regression differentially modulate the expressions of m^6^A-RMRs. We collected a dataset of scRNA-Seq in the scRNA-Seq database in the Broad Institute at MIT. As shown in Figure 4(c), the expressions of 14 out of 27 m^6^A-RMRs including Wtap, Pcif1, Alkbh5, Ythdc1, Ythdf1, Ythdf2, Ythdf3, Hnrnpa2b1, Eif3a, Fmr1, Hnrnpc, Prrc2a, G3bp1, and G3bp2 were decreased in the progressive atherosclerosis compared with those in the regressive atherosclerosis. The expression of Elavl1 was increased in the progressive atherosclerosis compared with that in the regressive atherosclerosis. Of note, the expressions of Virma and Igf2bp1 were not found. These results have illustrated that (1) atherosclerotic macrophages have higher upregulation of m^6^A-RMRs than atherosclerotic carotid artery, and FHC and atherosclerotic monocytes have more modulation of m^6^A-RMRs than FHC and atherosclerotic T cells; (2) type 2 diabetes adipose tissues have the highest modulation of m^6^A-RMRs (11 upregulated and three downregulated) among the 14 metabolic disease datasets; (3) obesity diseases have more downregulation than upregulation of m^6^A-RMRs, and (4) nearly half of 29 m^6^A-RMRs are downregulated as atherosclerosis progression compared with that of atherosclerosis regression.

Additionally, for further understanding the m^6^A-RMRs's modulation in cardiovascular diseases, our study of m^6^A-RMRs in atherosclerosis and other three studies of m^6^A-RMRs in cardiovascular disease (CVDs) [60–62] were compared. From Table 4, METTL14 is upregulated in our study and other two studies; FTO is downregulated in our study and other two studies. At least, these data revealed that in atherosclerosis and other CVDs, m^6^A writer METTL14 is upregulated and eraser FTO is downregulated.

### 3.3. Inflammatory Bowel Diseases and Rheumatoid Arthritis Modulate m^6^A-RMRs More Than Autoimmune Lupus and Psoriasis; and Some Autoimmune Diseases Share Five Upregulated m^6^A-RMRs such as PCIF1, G3BP2, G3BP1, WTAP, and FTO

**Figure 5:**
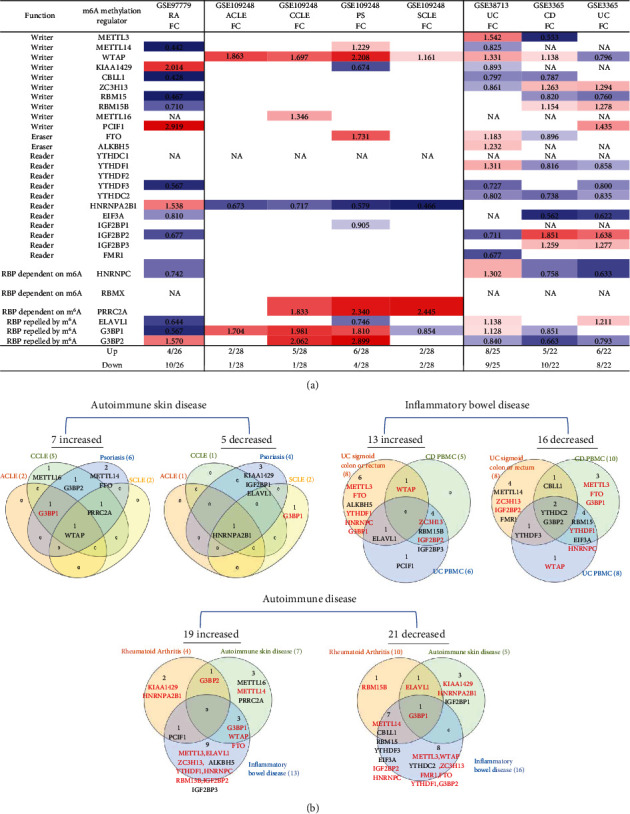
The m^6^A-RNA methylation regulators (m^6^A-RMRs) were more differentially expressed in rheumatoid arthritis (RA) and autoimmune skin diseases than those in other autoimmune diseases. (a) The expression changes of m^6^A-RMRs showed the higher expression fold changes in RA and skin autoimmune diseases than those in inflammatory bowel disease. KIAA1429 and PCIF1 were highly increased; and METTL14, CBLL1, and RBM15 were highly decreased in RA; WTAP, PRRC2A, and G3BP2 were highly increased genes in skin autoimmune disease psoriasis. (b) Venn diagram showed that there were seven upregulated m^6^A-RMRs and five downregulated m^6^A-RMRs in autoimmune skin diseases. The writer WTAP and reader HNRNPA2B1 were the common increased and decreased genes in autoimmune skin diseases. There were 13 upregulated m^6^A-RMRs and 16 downregulated m^6^A-RMRs in inflammatory bowel diseases. YTHDC2 and G3BP2 were the common downregulated m^6^A-RMRs. PCIF1 was the shared upregulated m^6^A-RMR by RA and inflammatory bowel disease. CBLL1, RBM15, YTHDF3, and EIF3A were the shared downregulated m^6^A-RMRs by RA and inflammatory bowel disease. There were 19 upregulated m^6^A-RMRs and 21 downregulated m^6^A-RMRs in these three types of autoimmune diseases. Note: the red marked genes are those that are up- or downregulated in different diseases (*p* < 0.05). Abbreviations: RA: rheumatoid arthritis; ACLE: acute cutaneous lupus; CCLE: chronic cutaneous lupus; PS: psoriasis; SCLE: subacute cutaneous lupus; UC: ulcerative colitis; CD: Crohn's disease. FC: fold change; NA: not available (missing value).

We hypothesized that major autoimmune diseases including rheumatoid arthritis (RA), psoriasis (PS), acute cutaneous lupus (ACLE), chronic cutaneous lupus (CCLE), subacute cutaneous lupus (SCLE), Crohn's disease (CD), and ulcerative colitis (UC) differentially modulate the expressions of m^6^A-RMRs. To examine this hypothesis, we collected four microarray datasets (eight comparisons) from the NCBI-GeoDatasets (https://www.ncbi.nlm.nih.gov/gds/). In a RA dataset, four out of 26 m^6^A-RMRs were upregulated; and 10 out of 26 m^6^A-RMRs were downregulated. In four lupus datasets, two to six out of 28 m^6^A-RMRs were upregulated; and one to four out of 28 m^6^A-RMRs were downregulated. In three colitis datasets, five to eight m^6^A-RMRs were upregulated; and eight to nine m^6^A-RMRs were downregulated (Figure 5).

We also analyzed the shared and disease-specific m^6^A-RMRs using Venn diagram analysis. In seven m^6^A-RMRs upregulated in the autoimmune skin disease group, one m^6^A-RMR WTAP was shared by four autoimmune skin diseases; one m^6^A-RMR G3BP1 was shared by three autoimmune skin diseases including ACLE, CCLE, and psoriasis; one m^6^A-RMR PRRC2A was shared by three autoimmune skin diseases including CCLE, psoriasis, and SCLE; and one m^6^A-RMR G3BP2 was shared by two autoimmune skin diseases including CCLE and psoriasis. In 13 m^6^A-RMRs upregulated in the inflammatory bowel disease group, one m^6^A-RMR ELAVL1 was shared by UC sigmoid colon or rectum and UC PBMCs; one m^6^A-RMR WTAP was shared by UC sigmoid colon or rectum and CD PBMCs; and four m^6^A-RMRs including ZC3H13, RBM15B, IGF2BP2, and IGF2BP3 were shared by CD PBMCs and UC PBMCs. In 19 m^6^A-RMRs upregulated in all eight autoimmune diseases, one m^6^A-RMR PCIF1 was shared by RA and inflammatory bowel disease; one m^6^A-RMR G3BP2 was shared by RA and autoimmune skin diseases; and three m^6^A-RMRs including G3BP1, WTAP, and FTO were shared by autoimmune skin diseases and inflammatory bowel diseases. Taken together, our results have shown that first, inflammatory bowel diseases and RA modulate m^6^A-RMRs more than autoimmune lupus and psoriasis; second, autoimmune skin diseases share four upregulated m^6^A-RMRs including G3BP1, G3BP2, PRRC2A, and WTAP; third, inflammatory bowel diseases share six upregulated m^6^A-RMRs including ELAVL1, WTAP, ZC3H13, RBM15B, IGF2BP2, and IGF2BP3; and fourth, autoimmune diseases have no any commonly shared m^6^A-RMRs but share five upregulated m^6^A-RMRs between groups including PCIF1, G3BP2, G3BP1, WTAP, and FTO.

### 3.4. Some Organ Failures Share Eight Upregulated m^6^A-RMRs between Groups including Four Writers WTAP, PCIF1, RBM15, and RBM15B; Two m^6^A-Dependent RNA Binding Proteins PRRC2A and HNRNPC; and Two m^6^A-Repelled RNA Binding Proteins IGF2BP2 and IGF2BP3; End-Stage Renal Failure (ESRF) Downregulates 85% of 26 m^6^A-RMRs, and Upregulation of 45% m^6^A-RMRs in Hemodialysis from ESRF (15%) May Be Associated with Clinical Benefits

**Figure 6:**
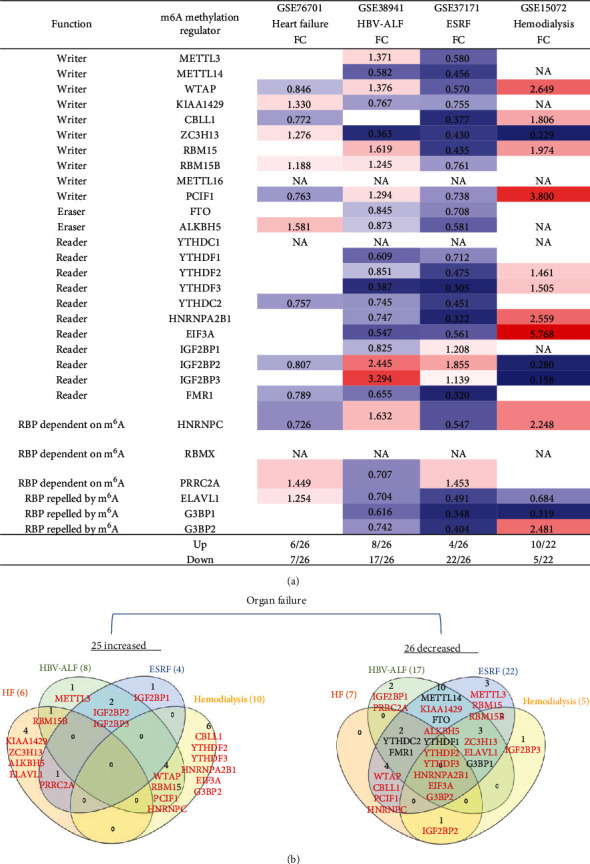
The m^6^A-RNA methylation regulators (m^6^A-RMRs) were differentially expressed in several different organ failure diseas**es**. (a) More m^6^A-RMRs were significantly increased and decreased in hepatitis B virus- (HBV-) associated acute liver failure (HBV-ALF) and hemodialysis diseases. The expression fold changes of IGF2BP1 and IGF2BP2 were higher, and the expression fold changes of ZC3H13 and YTHDF3 were lower in HBV-ALF. The expression fold changes of most m^6^A-RMRs were lower in whole blood of end-stage renal failure (ESRF) while the expression fold changes of some m^6^A-RMRs (WTAP, PCIF1, and EIF3A) were higher in PBMC of hemodialysis compared with healthy control. (b) Venn diagram showed that there were no shared upregulated or downregulated m^6^A-RMRs among these four studies. Some m^6^A-RMRs were decreased between organ failure diseases: YTHDC2 and FMR1 were shared between HF and HBV-ALF; METTL14, FTO, and YTHDF1 were shared between HBV-ALF and ESRF; G3BP1 was shared between HBV-ALF and ESRF. Note: the red marked genes are those that are up- or downregulated in different diseases (*p* < 0.05). Abbreviations: HBV-ALF: hepatitis B virus- (HBV-) associated acute liver failure; CKD: chronic kidney disease; ESRF: end-stage renal failure; FC: fold change; NA: not available (missing value).

We hypothesized that major organ failures including heart failure, hepatitis B virus-associated acute liver failure (HBV-ALF), end-stage renal failure (ESRF), and hemodialysis modulate the expressions of m^6^A-RMRs. We collected four microarray datasets from the NCBI-GeoDatasets (https://www.ncbi.nlm.nih.gov/gds/) to examine this hypothesis. As shown in Figure 6, in the heart failure dataset, six out of 26 m^6^A-RMRs were upregulated; and seven out of 26 m^6^A-RMRs were downregulated. In the HBV-ALF dataset, eight out of 26 m^6^A-RMRs were upregulated; and 17 out of 26 m^6^A-RMRs were downregulated. In the ESRF dataset, four out of 26 m^6^A-RMRs were upregulated; and 22 out of 26 m^6^A-RMRs were downregulated. In the hemodialysis dataset, 10 out of 22 m^6^A-RMRs were upregulated; and five out of 22 m^6^A-RMRs were downregulated. We also analyzed the shared and disease-specific m^6^A-RMRs using Venn diagram analysis. In 20 m^6^A-RMRs upregulated in four organ failure groups, one upregulated m^6^A-RMR (RBM15B, RNA methyltransferase) was shared by HF and HBV-ALF; one upregulated m^6^A-RMR (PRRC2A) was shared by HF and ESRF; two upregulated m^6^A-RMRs (IGF2BP2 and IGF2BP3, RNA binding proteins) were shared by HBV-ALF and ESRF; and four m^6^A-RMRs including WTAP (RNA methyltransferase), RBM15 (RNA methyltransferase), PCIF1 (RNA methyltransferase), and HNRNPC (m^6^A-dependent RNA binding protein) were shared by HBV-ALF and hemodialysis.

These results demonstrated that first, four major organ failures have no commonly shared upregulated m^6^A-RMRs but share eight m^6^A-RMRs between groups including RBM15B, IGF2BP2, IGF2BP3, PRRC2A, WTAP, RBM15, PCIF1, and HNRNPC; second, hemodialysis is the only organ failure that upregulates more m^6^A-RMRs than downregulates m^6^A-RMRs; other organ failures have less upregulation of m^6^A-RMRs than downregulation of m^6^A-RMRs; third, surprisingly, ESRF and hemodialysis have no any shared upregulated m^6^A-RMRs, suggesting that the clinical improvements of hemodialysis from ESRF are benefited from upregulation of m^6^A-RMRs by hemodialysis; and fourth, ESRF modulates 100% of 26 m^6^A-RMRs, which is the highest modulation rate found among all the diseases examined.

### 3.5. MERS-CoV Infections at 36 Hours in Human Microvascular Endothelial Cells Modulate the Highest Numbers of m^6^A-RMRs (Upregulated 20 and Downregulated Four) among Three Types of Viral Infections (MERS-CoV, SARS-CoV, and Influenza Virus); and the Differences in Modulating the Expressions of m^6^A-RMRs May Be Related to the Virulence of Virus and Virus Replication

**Figure 7:**
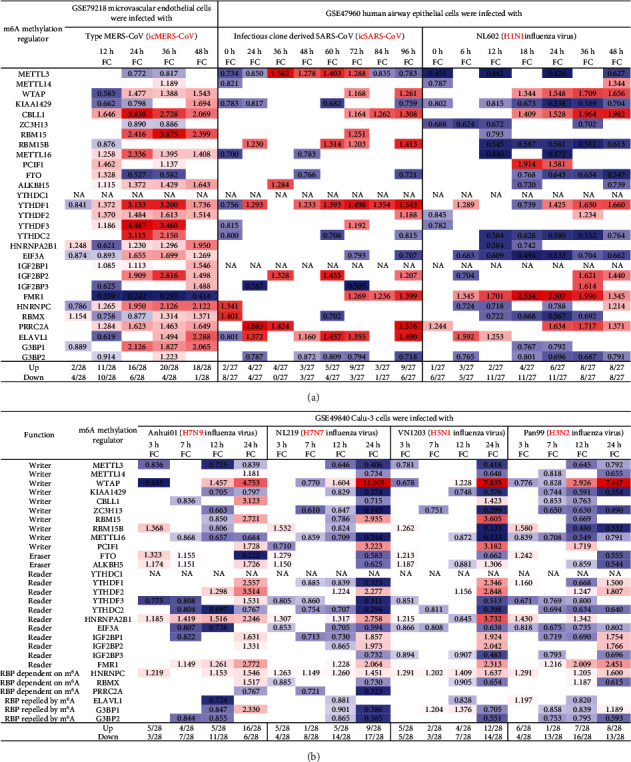
The m^6^A-RNA methylation regulators (m^6^A-RMRs) were upregulated in virus-infected cells as the infection time was extended. (a) In general, upregulated m^6^A-RMRs were more than downregulated m^6^A-RMRs in the condition of MERS and SARS infections. The expressions of m^6^A methylation writers such as WTAP, KIAA1429, CBLL1, and RBM15 and readers YTHDF1, YTHDF2, YTHDF3, and RBP dependent on m^6^A PRRC2A were increased; and the expression of eraser FTO was decreased when endothelial cells were infected with MERS coronavirus (MERS-CoV) and SARS coronavirus (SARS-CoV). (b) In avian influenza virus studies, it was obvious that the expressions of writer WTAP and PCIF1 were increased, and the expression of eraser FTO was decreased in all avian influenza virus-infected cells; the longer the infected time was, the higher the numbers of differentially expressed m^6^A methylation regulators there were. The results suggest that MERS coronavirus, SARS coronavirus, and avian influenza virus infections upregulated the expressions of m^6^A-RMRs (*p* < 0.05). Abbreviations: MERS: Middle East respiratory syndrome; SARS: severe acute respiratory syndrome; H1N1, H7N9, H7N7, H5N1, and H3N2 are the cell populations of avian influenza virus. FC: fold change; NA: not available (missing value).

We hypothesized that viral infections with Middle East respiratory syndrome (MERS) coronavirus infection [63], severe acute respiratory syndrome (SARS) coronavirus (SARS-CoV) [64], and influenza virus infections [65] modulate the expressions of m^6^A-RMRs. To test this hypothesis, we collected three groups of microarray datasets from the NCBI-GeoDatasets (https://www.ncbi.nlm.nih.gov/gds/). We found that MERS-CoV infection of human microvascular endothelial cells at the time course of 0, 12, 24, 36, and 48 hours (h) [63] after infection resulted in upregulation of two to 20 out of 28 m^6^A-RMRs and downregulation of one to 10 out of 28 m^6^A-RMRs (Figure 7). In addition, SARS-CoV infection of human airway epithelial cells at the time course of 0, 24, 36, 48, 60, 72, 84, and 96 hours (h) [64] after infection resulted in upregulation of two to nine out of 27 m^6^A-RMRs and downregulation of zero to eight out of 27 m^6^A-RMRs. H1N1 influenza virus infection of human airway epithelial cells at the time course of 0, 6, 12, 18, 24, 36, and 48 hours after infection resulted in upregulation of one to eight out of 27 m^6^A-RMRs and downregulation of five to 11 out of 27 m^6^A-RMRs. Moreover, infections of Calu-3 cells (a non-small-cell lung cancer cell line) with four different strains of influenza viruses (H7N9, H7N7, H5N1, and H3N2) [65] resulted in upregulation of four to 16, one to nine, two to 12, and one to eight out of 28 m^6^A-RMRs, respectively, and downregulation of three to 11, four to 17, three to 14, and four to 16 out of 28 m^6^A-RMRs, respectively. Taken together, these results have demonstrated that (1) MERS-CoV infections in human microvascular endothelial cells modulate the highest numbers of m^6^A-RMRs (upregulated 20 and downregulated four at the time course of 36 hours) among the three types of viral infections examined; (2) similar to MERS-CoV infection, SARS-CoV infections in human airway epithelial cells upregulated more m^6^A-RMRs than downregulated but significantly less than that in MERS-CoV infections; (3) influenza virus (H1N1) infection in human airway epithelial cells downregulated more m^6^A-RMRs than upregulated; and (4) novel avian-origin influenza virus (IAV) H7N9 infections at 24-hour postinfection upregulated 16 and downregulated six out of 28 m^6^A-RMRs, which are significantly different from that of upregulation of nine, 12, and eight and downregulation of 17, 14, and 13 out of 28 m^6^A-RMRs by highly pathogenic avian-origin influenza viruses H7N7, H5N1, and human seasonal H3N2 IAV, respectively. The differences in modulating the expressions of m^6^A-RMRs may be related to the virulence of the virus, virus replication, and other aspects [65].

### 3.6. Proinflammatory Lipid oxPAPC and NOTCH1 Knockdown Modulates m^6^A-RMRs More Than Other DAMP Stimulation of ECs including LPS, oxLDL, and IFNs; and Two m^6^A-RMRs Such as Hnrnpa2b1 and Eif3a Were Differentially Expressed in Three Aortic EC Clusters

**Figure 8:**
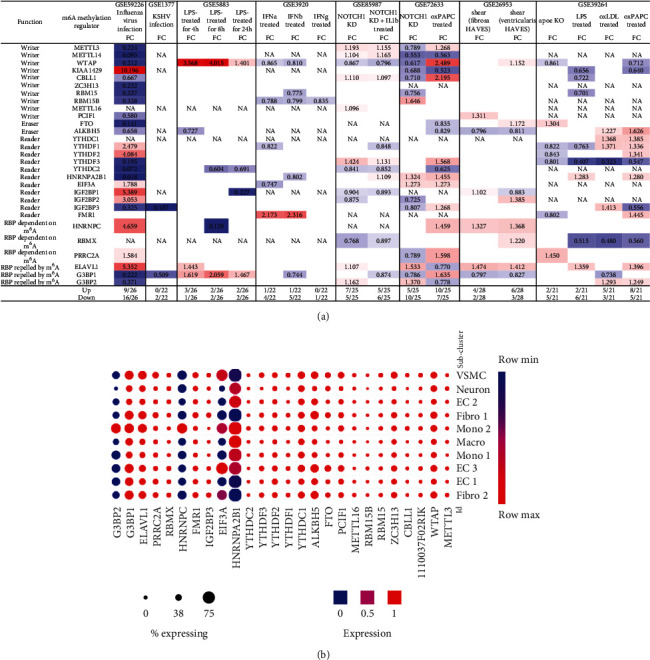
The expressions of m^6^A-RNA methylation regulators (m^6^A-RMRs) were modulated in endothelial cells with different stimulations. (a) The expression changes of m^6^A-RMRs were the most obvious in influenza virus infections with the highest expression FC values. The expressions of writer KIAA1429 and readers YTHDF1, YTHDF2, IGF2BP1, IGF2BP2, HNRNPC, and ELAVL1 were upregulated with the FC > 2; the expressions of writers METTL3, METTL14, WTAP, ZC3H13, RBM5, RBM15B; eraser FTO; and readers YTHDF3, YTHDC2, HNRNPA2B1, IGF2BP3, G3BP1, and G3BP2 were downregulated with the FC < 0.5. In KSHV-infected ECs, IGF2BP3 was downregulated with low FC values. In LPS-treated microvascular ECs, writer WTAP was upregulated in four and eight hours. In IFNa- and IFNb-treated cells, FMR1 was increased. In the cells with NOTCH1 knockdown combined with IL-1*β* treatment, the expression changes of m^6^A-RMRs were not obvious. ECs activated with ox-PAPC showed that the expressions of WTAP and CBLL1 were upregulated. There were gentle expression changes of m^6^A-RMRs for ECs exposed to shear or for mouse aortic ECs from apolipoprotein E- (ApoE-) deficient (ApoE knock out) or treated with LPS, oxLDL, and oxPAPC, respectively. (b) The m^6^A-RMRs were differentially expressed in aortic EC populations. The 12 out of 29 m^6^A-RMRs including Wtap, Zc3h13, Pcif1, FTO, Alkbh5, Ythdc1, YthDf2, Ythdf3, Fmr1, Prrc2a, Elavl1, and G3bp1 were in medium expression levels in almost all aortic cell populations of normal mouse aorta; and m^6^A-RMRs such as Hnrnpa2b1, Eif3a, Hnmpc, and G3bp2 were differentially expressed in aortic cell populations. (Mettl14, Igf2bp1, and Igf2bp2 not found) (*p* < 0.05). Abbreviations: KSHV: Kaposi sarcoma-associated herpes virus; oxPAPC: oxidized 1-palmitoyl-2-arachidonoyl-sn-glycero-3-phosphocholine (inflammatory lipids); oxLDL: oxidized LDL; FC: fold change; NA: not available (missing value); VSMC: vascular smooth muscle cells; EC: endothelial cells.

We hypothesized that pathogen-associated molecular patterns (PAMPs)/danger-associated molecular patterns (DAMPs) modulate the expressions of m^6^A-RMRs in various endothelial cells. To examine this hypothesis, we collected eight microarray datasets from various endothelial cells stimulated by PAMPs/DAMPs. As shown in Figure 8(a), influenza virus infection of human umbilical vein endothelial cells (HUVEC) resulted in upregulation of nine out of 26 m^6^A-RMRs (with the highest upregulation of writer KIAA1429 for 10 folds) and downregulation of 16 out of 26 m^6^A-RMRs. By comparison, Kaposi's sarcoma-associated herpesvirus (KSHV) infection of human dermal endothelial cells modulated m^6^A-RMR expressions significantly less than that of influenza virus infection, upregulating zero and downregulating two out of 22 m^6^A-RMRs. Toll-like receptor 4 (TLR4) ligand lipopolysaccharide (LPS) stimulation of human lung microvascular endothelial cells for 4, 8, and 24 hours resulted in upregulation of two to three m^6^A-RMRs (with the high upregulation of writer WTAP) and downregulation of one to two m^6^A-RMRs, respectively. In addition, cytokine interferon-*α* (IFN*α*), IFN*β*, and IFN*γ* treatments of HUVEC led to upregulation of zero to one and downregulation of one to five out of 22 m^6^A-RMRs, respectively. The knockdown (KD) of proinflammatory NOTCH1 signaling and NOTCH1 KD plus proinflammatory cytokine interleukin-1*β* (IL-1*β*) in HUVEC led to upregulation of seven and five out of 25 m^6^A-RMRs and downregulation of five and six out of 25 m^6^A-RMRs. The differences of m^6^A-RMR expression modulation between NOTCH1 KD and NOTCH1 KD plus IL-1*β* may contribute to the inflammatory status of treated HUVEC [66]. In contrast, another report found that NOTCH1 is antiatherogenic; and proinflammatory lipids oxidized 1-palmitoyl-2-arachidonoyl-sn-glycero-3-phosphocholine (Ox-PAPC) decrease NOTCH1 expression in human aortic endothelial cells (HAEC) [67]. In this experimental setting, NOTCH1 KD in HAEC resulted in upregulation of five and downregulation of 10 out of 25 m^6^A-RMRs; proinflammatory lipid oxPAPC stimulation in HAEC led to upregulation of 10 and downregulation of seven out of 25 m^6^A-RMRs. It was reported that oscillatory shear (OS) present on the fibrosa stimulates fibrosa human aortic valve endothelial cells (HAVEC), which may contribute to aortic valve disease [68]. In this experimental setting, oscillatory shear on fibrosa HAVEC and ventricularis HAVEC resulted in upregulation of four and six and downregulation of two and three out of 28 m^6^A-RMRs, respectively. Moreover, mouse aortic endothelial cells (MAEC) isolated from atherogenic apolipoprotein E-deficient (ApoE^−/−^) mice [3], TLR4 ligand LPS-treated MAEC, another TLR4 ligand oxidized low-density lipoprotein- (oxLDL-) stimulated MAEC [69, 70], and oxPAPC-stimulated MAEC [71] upregulated two, two, five, and eight out of 21 m^6^A-RMRs and downregulated five, six, three, and five out of 21 m^6^A-RMRs, respectively.

We then examined the expressions of m^6^A-RMRs in three mouse aortic endothelial cell (EC) clusters identified recently with single-cell RNA sequencing (scRNA-Seq) [72]. As shown in Figure 8(b), 12 out of 26 m^6^A-RMRs (46%) including Wtap, Zc3h13, Pcif1, FTO, Alkbh5, Ythdc1, YthDf2, Ythdf3, Fmr1, Prrc2a, Elavl1, and G3bp1 were in medium expression levels in almost all aortic cell populations of normal mouse aortas; and two m^6^A-RMRs such as Hnrnpa2b1 and Eif3a were differentially expressed in three aortic EC clusters, which were Cytl1^+^Gkn3^+^ endothelial cell (EC) cluster 1, Fabp4^+^Gphbp1^+^Rgcc^+^ EC cluster 2, and Ccl21a^+^Lrg1^+^ EC cluster 3 [72]. Of note, the expressions of Mettl14, Igf2bp1, and Igf2bp2 were not found.

These results conclude that (1) influenza virus infection of HUVEC results in expression modulation of most m^6^A-RMRs (25 out of 26), which were similar to that found in influenza virus infections of human airway epithelial cells and Calu-3 lung cells for 24 hours in Figure 7; (2) proinflammatory lipids oxPAPC and NOTCH1 knockdown modulate m^6^A-RMRs more than other DAMP stimulation of ECs including LPS, oxLDL, and IFNs; (3) ten m^6^A methyltransferases were not significantly modulated in MAEC from ApoE KO aorta with or without further stimulations of LPS, oxLDL, and oxPAPC, respectively (Figure 8, GSE39264); and (4) 12 out of 26 m^6^A-RMRs (46%) were in medium expression levels in almost all aortic cell populations of normal mouse aortas; and two m^6^A-RMRs including Hnrnpa2b1 and Eif3a were differentially expressed in three aortic EC clusters.

### 3.7. Upregulated m^6^A-RMRs Were More Than Downregulated m^6^A-RMRs in Various Cancer Types; Head and Neck Cancer, Cervical Cancer, Brain and CNS Cancer, Other Cancer, and Kidney Cancer Upregulated ≥10 m^6^A-RMRs; and IGF2BP3, G3BP1, IGF2BP2, and HNRNPC Were Upregulated in ≥10 Cancer Types

**Figure 9:**
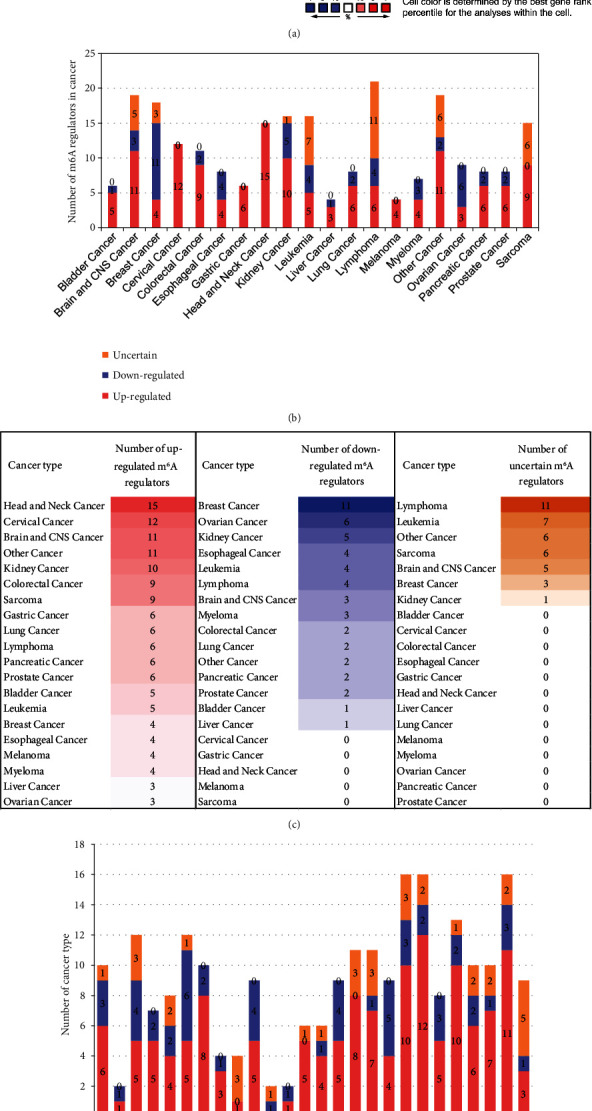
The m^6^A-RNA methylation regulators (m^6^A-RMRs) were differentially expressed in 18 cell populations of cancers in the Oncomine database (https://www.oncomine.org/). (a) The numbers in red were the numbers of m^6^A-RMRs upregulated, and the numbers in blue were the numbers of m^6^A-RMRs downregulated. The results showed that m^6^A-RMRs upregulated were more than m^6^A methylation regulators downregulated. The “Significant Unique Analyses” indicated the numbers of studies in which the analyzed genes were significantly upregulated (red) or downregulated (blue). The “Total Unique Analysis” indicated the numbers of studies in which the analyzed genes were included (*p* < 0.05, fold change > 2) (METTL16, YTHDC1, and PRRC2A not found). (b) Different expression patterns of m^6^A-RMRs in different cell populations of cancers. The bar graph showed differential expression m^6^A regulators IGF2BP2 (upregulated in ten cancers, downregulated in three cancers, and uncertain in three cancers), IGF2BP3, and G3BP1 are the top 3 genes involved in different cancers. (c) IGF2BP3, G3BP1, and IGF2BP2/HNRNPC were upregulated in 12, 11, and 10 cancers, respectively. (d) ZC3H13, IGF2BP1, and WTAP/FTO/YTHDC2 were downregulated in six, five, and four cancers, respectively. (e) The expression of G3BP2 in five cancers was uncertain; WTAP, PCIF1, HNRNPA2B1, EIF3A, and IGF2BP2 in three cancers were uncertain.

We hypothesized that the expressions of m^6^A-RMRs are modulated in various cancer cell populations. To test this hypothesis, we collected 19 cancer datasets in a comprehensive cancer gene expression database Oncomine (https://www.oncomine.org) [45] including brain and CNS cancer, head and neck cancer, esophageal cancer, gastric cancer, bladder cancer, breast cancer, cervical cancer, ovarian cancer, colorectal cancer, kidney cancer, liver cancer, lung cancer, pancreatic cancer, prostate cancer, leukemia, lymphoma, melanoma, myeloma, sarcoma, and other cancer (Figure 9(a)). As shown in Figure 9, the numbers in red indicated the numbers of studies with upregulated m^6^A-RMRs; and the numbers in blue indicated the numbers of studies with downregulated m^6^A-RMRs. The results showed that upregulated m^6^A-RMRs were more than downregulated m^6^A-RMRs in various cancer types (number of red cells is more than that of blue cells). Of note, the “Significant Unique Analyses**”** indicated the numbers of studies in which the analyzed genes were significantly upregulated (red) or downregulated (blue). The “Total Unique Analysis” indicated the numbers of studies in which the analyzed genes were included (*p* < 0.05, fold change > 2). Three m^6^A-RMRs such as METTL16, YTHDC1, and PRRC2A were not found.

We then determined the top cancer types that upregulated or downregulated m^6^A-RMRs the most. As shown in Figures 9(b) and 9(c), head and neck cancer, cervical cancer, brain and CNS cancer, other cancer, and kidney cancer upregulated ≥10 m^6^A-RMRs. In contrast, breast cancer, ovarian cancer, kidney cancer, esophageal cancer, leukemia, and lymphoma downregulated ≥four m^6^A-RMRs. In addition, lymphoma, leukemia, other cancer, sarcoma, and brain and CNS cancer modulated ≥five m^6^A-RMRs in uncertain manners. We also determine the top m^6^A-RMRs that upregulated or downregulated in cancer types the most. As shown in Figures 9(d) and 9(e), IGF2BP3, G3BP1, IGF2BP2, and HNRNPC were upregulated in ≥10 cancer types. In contrast, ZC3H13, IGF2BP1, WTAP, FTO, and YTHDC2 were downregulated in ≥four cancer types. In addition, G3BP2, WTAP, PCIF1, HNRNPA2B1, EIF3A, and IGF2BP2 were modulated in ≥three cancer types in uncertain manners.

The results indicated that most m^6^A-RMRs were downregulated in acute inflammatory diseases, while in cancer, the upregulated m^6^A-RMRs were more than downregulated m6A-RMRs. This situation is because acute inflammation is a part of an innate immune system reaction that can be produced by various factors, such as signaling pathways of the receptors for danger-associated molecule patterns (DAMPs/PAMPs) derived from pathogens, viruses, bacteria, and toxic substances as well as cytokine signals and stress hormone signal. These factors may activate the interactions and cross-talks among the receptors of cytokines, viruses, DAMPs, or PAMPs and promote the migration of macrophages or neutrophils to the area of inflammation [73]. However, during cancer occurs, tumor cells will generate tumor antigens that activate adaptive immune responses and induce T cells and B cells. T cell activation could cause a variety of immune signaling, including T cell antigen receptor signaling, costimulation signaling, immune checkpoint coinhibition signaling, and cytokine signaling. The composition of signaling receptors and pathways is different between acute inflammation and cancer. Therefore, RNA methylation and its transcription, splicing, and mRNA stability are also different under these two situations.

We further hypothesized that m^6^A-RMRs were differentially expressed as different cancer progression. To examine this hypothesis, the expression matrix GSE114783 was downloaded from the NIH-NCBI-GeoDataset database (https://www.ncbi.nlm.nih.gov/gds); and the expressions of m^6^A-RMRs were screened. The heat map was generated from Cluster web tool [45]. As shown in Supplementary figure 1A, the results showed that the expressions of m^6^A-RMRs were different among healthy controls, hepatitis B virus carriers, and the patients with liver cirrhosis and hepatocellular carcinoma, especially genes in the red box; compared with healthy controls, there were more upregulated m^6^A-RMRs in patients with chronic hepatitis B (CHB) group than those in healthy controls. Potentially, due to the tumor heterogeneity or the small sample sizes, there were no differentially expressed m^6^A-RMRs in the hepatocellular carcinoma group compared with healthy controls. In addition, as shown in Supplementary figure 1B, using the same method as Supplementary figure 1A, the expressions of m^6^A-RMRs were analyzed in human preinvasive and invasive cervical squamous cell carcinomas and normal cervical epithelia. The differential expressions of m^6^A-RMRs were shown in the red box. The 22 m^6^A-RMRs were examined in these three groups. The results showed that the expressions of WTAP were downregulated; and the expressions of four m^6^A-RMRs such as HNRNPA2B1, IGF2BP2, FMR1, and HNRNPC were upregulated in patients with preinvasive and invasive cervical squamous cell carcinomas compared with normal controls. Moreover, as shown in Supplementary figure 1C, the expressions of m^6^A-RMRs were analyzed in prostate cancer from benign prostatic hyperplasia to metastatic prostate cancer. The expressions of RBMX were gradually increased from prostatic intraepithelial neoplasia to hormone-naïve prostate cancer compared with normal samples. There were more obvious differential expressions of m^6^A-RMRs between adjacent prostate cancer samples and prostate carcinoma samples. The expressions of two m^6^A-RMRs such as EIF3A and RBMX were upregulated in prostate carcinoma samples compared with normal prostate epithelium-adjacent samples.

Finally, we examined the expressions of 29 m^6^A-RMRs [74] in single-cell RNA-Seq data. As shown in Supplementary figure 2A, in a single-cell RNA-seq analysis of astrocytoma dataset [75], 23 out of 28 m^6^A-RMRs (82%) (except ALKBH5, IGF2BP1, IGF2BP2, IGF2BP3, and G3BP2) were in the medium to high expression levels in malignant cells of astrocytoma. In addition, as shown in Supplementary figure 2B, in a melanoma intratumor heterogeneity dataset [76], 24 out of 29 m^6^A-RMRs (83%) (except RBM15B, IGF2BP1, IGF2BP3, HNRNPA2B1, and HNRNPC) were in the medium to high expression levels in cancer-associated fibroblasts (CAF) of melanoma.

Taken together, these results have demonstrated that first, upregulated m^6^A-RMRs were more than downregulated m^6^A-RMRs in various cancer types; second, head and neck cancer, cervical cancer, brain and CNS cancer, other cancer, and kidney cancer upregulated ≥10 m^6^A-RMRs; third, IGF2BP3, G3BP1, IGF2BP2, and HNRNPC were upregulated in ≥10 cancer types; fourth, four m^6^A-RMRs such as HNRNPA2B1, IGF2BP2, FMR1, and HNRNPC were upregulated in patients with preinvasive and invasive cervical squamous cell carcinomas; and the expressions of two m^6^A-RMRs such as EIF3A and RBMX were upregulated in prostate carcinoma samples; and fifth, 82-83% of 29 m^6^A-RMRs were in the medium to high expression levels in astrocytoma and melanoma.

### 3.8. M1 Macrophage Polarization Upregulates Seven m^6^A-RMRs including METTL14, WTAP, PCIF1, HNRNPA2B1, IGF2BP2, FMR1, and G3BP1, among Which WTAP and IGF2BP2 May Not Only Promote M1 Polarization but Also Inhibit M2 Polarization

**Figure 10:**
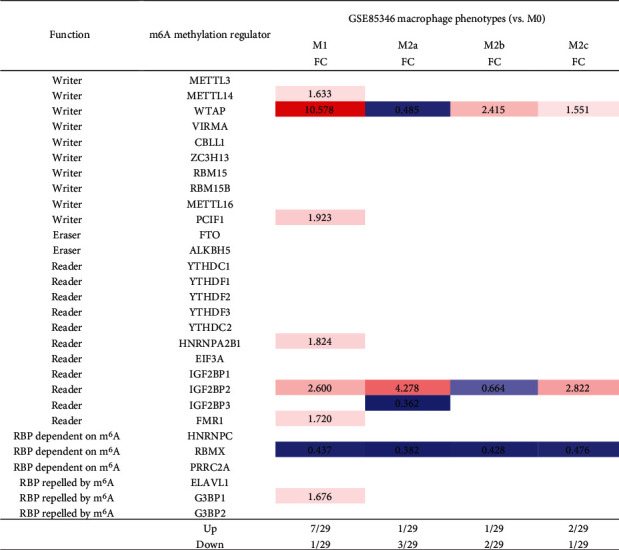
Macrophage polarization regulated the expression of m^6^A-RNA methylation regulators (m^6^A-RMRs). Macrophage polarization microarray analysis result showed that expression of WTAP is significantly upregulated in M1 with a fold change of 10.578 and in M2b with a fold change of 2.415 compared with M0 cells; expression of IGF2BP2 is upregulated in M1, M2a, and M2c cells; RBMX is the common downregulated m^6^A-RMRs in M1, M2a, M2b, and M2c macrophages.

We hypothesized that macrophage polarization from M0 into type 1 proinflammatory macrophages (M1) or M2 anti-inflammatory macrophages modulate the expressions of m^6^A-RMRs. To examine this hypothesis, we collected a microarray dataset GSE85346 from the NCBI-GeoDataset database. As shown in Figure 10, M1 macrophage polarization upregulated seven m^6^A-RMRs including METTL14, WTAP, PCIF1, HNRNPA2B1, IGF2BP2, FMR1, and G3BP1 whereas M2 polarization into M2a, M2b, or M2c downregulated three m^6^A-RMRs including WTAP, IGF2BP2, and IGF2BP3. These results suggest that these M1 polarization upregulated seven m^6^A-RMRs may promote proinflammatory M1 polarization. In contrast, two M1 polarization-upregulated and M2 polarization-downregulated m^6^A-RMRs such as WTAP and IGF2BP2 may not only promote M1 polarization but also inhibit M2 polarization.

Macrophages are diverse immune cells polarized by numerous stimuli, resulting in a wide range of traits and functions. The polarized process from M0 to M1 is induced by the stimulations of TLR ligands or Th1 cytokines, like TNF-a, IFN-*γ*, and CSF2 signaling. Under inflammation, these macrophage polarization signals are specific and robust [77], whereas, during tumorigenesis, so many factors can be involved. Moreover, different cancers are related to different cell types. A great amount of different signaling cross-talks may cause chaos signaling [78]. Consequently, the signaling that modulates the upregulation of WTAP could be diffused by the chaos cross-talking signaling.

### 3.9. The 86% of m^6^A-RMRs Were Differentially Expressed in the Six Spleen Treg Clusters; and 8 Out of 12 Treg Signature Genes including FOXO1, HDAC9, Dicer, BLIMP1, GATA3, EP300, BCL6, and PPAR Significantly Regulated the Expressions of m^6^A-RMRs

**Figure 11:**
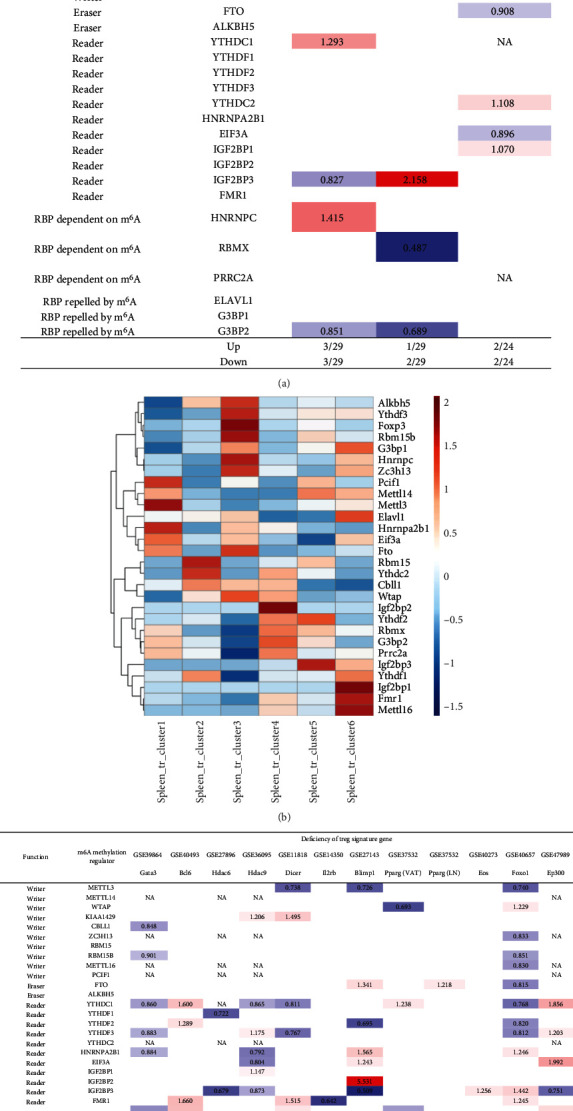
Regulatory T cells regulate the expression of m^6^A-RNA methylation regulators (m^6^A-RMRs). (a) Compared with conventional T cells, expression of IGF2BP3 is upregulated and RBMX is downregulated in regulatory T cells. (b) The expression data matrix was obtained from scRNA study (PMID: 29434354), and the expression heat map using Cluster web tool (PMID: 25969447) was generated. The result showed that the expression of m^6^A-RMRs in six types of Treg clusters was different. Expression of Ythdf3 and Rbm15b have the same pattern as Treg signature gene Foxp3. (c) Most m^6^A-RMRs are downregulated in deficiencies of GATA3, HDAC6, HDAC9, IL2RB, and FOXO1. The numbers of upregulated regulators are more than those of downregulators in deficiencies of BCL6, DICER, BLIMP1, PPARG, EOS, and EP300.

Our and others' reports demonstrated that CD4^+^Foxp3^+^ regulatory T cells play significant roles in suppressing immune responses, inflammations, atherosclerosis [13, 25–27, 70, 79–83], and antitumor immune reactions [84] and promoting tissue regeneration. We hypothesized that m^6^A-RMRs are modulated in Treg differentiation and Treg responses to tissue microenvironment. As shown in Figure 11(a), Treg upregulated six m^6^A-RMRs such as ZC3H13, YTHDC1, YTHDC2, IGF2BP1, IGF2BP3, and HNRNPC in three Treg datasets compared to that of CD4^+^Foxp3^−^ effector T cells.

In addition, a recent report with single-cell RNA sequencing (scRNA-Seq) characterizes spleen Treg into six clusters including S100a4highS100a6high cluster 1 (activated), Itgb1high cluster 2 (activated), Dusp2highNr4a1highFoxp3highIL2rahigh cluster 3 (activated), Ikzf2highFoxp3high cluster 4 (resting), Bach2high cluster 5 (resting), and Satb1highSellhigh cluster 6 (resting) [85]. We hypothesized that the m^6^A-RMRs are differentially expressed in these six Treg clusters. As shown in Figure 11(b), four m^6^A-RMRs including PCIF1, METTL3, HNRNPA2B1, and EIF3A were upregulated in the cluster 1 Treg; two m^6^A-RMRs including RBM15 and YTHDC2 were upregulated in the cluster 2 Treg; seven m^6^A-RMRs including ALKBH5, YTHDF3, RBM15B, HNRNPC, ZC3H13, FTO, Wand TAP were upregulated in cluster 3 Treg; four m^6^A-RMRs including IGF2BP2, RBMX, G3BP2, and PRRC2A were upregulated in cluster 4 Treg; three m^6^A-RMRs including METTL14, YTHDF2, and IGF2BP3 were upregulated in cluster 5 Treg; and five m^6^A-RMRs including G3BP1, ELAVL1, IGF2BP1, FMR1, and METTL16 were upregulated in cluster 6 Treg. Therefore, 25 out of 29 m^6^A-RMRs (86%) were differentially expressed in the six spleen Treg clusters.

Moreover, we hypothesized that Treg signature genes play significant roles in regulating the expressions of m^6^A-RMRs. To examine this hypothesis, we collected the microarray datasets of 12 Treg signature gene deficiencies. As shown in Figure 11(c), the deficiencies of type 2 T helper (Th2) transcription factor (TF) GATA binding protein 3 (GATA3), follicular Th cell (Tfh) TF B cell lymphoma 6 protein (BCL6), histone deacetylase 6 (HDAC6), HDAC9, microRNA maturation enzyme DICER, interleukin-2 receptor b chain (IL2rb), B-lymphocyte-induced maturation protein 1 (BLIMP1, PR domain Zinc finger protein 1), peroxisome proliferator activated receptor gamma (PPARg) (visceral adipose tissue, VAT), PPARg (lymph nodes, LN), IKAROS family zinc finger 4 (EOS), Foxhead box 1 (FOXO1), and histone acetyltransferase P300 (EP300) modulated and upregulated 1, 6, 0, 3, 5, 0, 6, 3, 1, 1, 6, and 5 m^6^A-RMRs, respectively, and downregulated 6, 0, 2, 5, 3, 1, 3, 2, 0, 0, 11, and 2 m^6^A-RMRs, respectively. The 12 Treg signature genes were ranked based on the total modulation numbers when they were deficient, FOXO1 (17) > HDAC9 (8) = Dicer (8) = BLIMP1 (8) > GATA3 (7) = EP300 (7) > BCL6 (6) > PPARg (VAT) (5) > HDAC6 (2) > IL − 2RB (1) = PPARG (LN) (1) = EOS (1). It has been reported that mechanistic target of rapamycin kinase (mTOR)/interferon regulatory factor 4 (IRF4)/GATA3 promotes IL-4^+^ Th2-like Treg; phosphatidylinositol-4,5-bisphosphate 3-kinase (PI3K)/AKT serine/threonine kinase 1 (Akt)/Foxo, Th1 TF T-box transcription factor 21 (T-bet), hypoxia inducible factor 1 subunit alpha (HIF-1a), glycolysis promote interferon-gamma (IFNg)^+^ Th1-like Treg, microbiota/MAF BZIP transcription factor (c-Maf)/signal transducer and activator of transcription 3 (Stat3)/RAR-related orphan receptor C (RORgt), aryl hydrocarbon receptor (AhR) promote IL-17+ Th17 like Treg, and mTOR/Stat3/hepatocyte nuclear factor 1-alpha (TCF1) promote Bcl6+ follicular Treg (Tfr).

Our results have shown that (1) Treg upregulated six m^6^A-RMRs including ZC3H13, YTHDC1, YTHDC2, IGF2BP1, and HNRNPC; (2) 25 out of 29 m^6^A-RMRs (86%) were differentially expressed in the six spleen Treg clusters, suggesting that m^6^A-RMRs play significant roles in reprogramming Treg activation status (clusters 1-3) and resting status (clusters 4-6); and (3) the transcription data from 12 Treg signature gene deficiencies suggest that eight out of 12 Treg signature genes including FOXO1, HDAC9, Dicer, BLIMP1, GATA3, EP300, BCL6, and PPAR*γ* regulate the expressions of m^6^A-RMRs significantly; and m^6^A-RMRs play important roles in reshaping Treg stability and plasticity.

### 3.10. Immune Checkpoint Receptors TIM3, TIGIT, PD-L2, and CTLA4 Play Significant Roles in Modulating the Expressions of m^6^A-RMRs; and Inhibition of Costimulation Receptor CD40 with Anti-CD40 Antibody Significantly Upregulates the Expressions of m^6^A-RMRs

**Figure 12:**
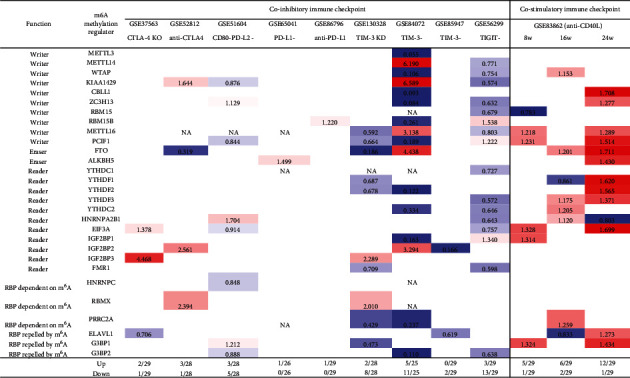
Immune checkpoint receptors regulate the expression of m^6^A-RNA methylation regulators (m^6^A-RMRs). T cell activation can regulate the expression of m^6^A-RMRs. Microarrays about immune checkpoint knockout (KO), knockdown (KD), anticheckpoint, or sorted negative and positive checkpoint were searched from the GEO database to analyze the regulation relationship between immune checkpoint and m^6^A-RMRs. The result showed that when the coinhibitory immune checkpoints PD-L2, TIM-3, and TIGIT were inhibited (KO or KD vs. control, anti- vs. control) or reduced (negative vs. positive), the expressions of m^6^A-RMRs were decreased. However, the costimulatory immune checkpoint CD40L was inhibited, and more m^6^A-RMRs' expressions were increased. That is, maybe, T cell activation negatively regulates the expression of m^6^A-RMRs.

We recently reported that cosignaling receptors localized on cell membrane including costimulation receptors and immune checkpoint receptors (coinhibition receptors) serve as a prototypic cell-cell contact interaction and regulate T cell plasticity, immune tolerance, cellular physiology, and sterile inflammatory disorders [86]. We hypothesized that cosignaling receptors regulate the expressions of m^6^A-RMRs. We collected 10 cosignaling receptor deficiency/suppression microarray datasets from the NIH-NCBI GeoDataset database. As shown in Figure 12, cytotoxic T-lymphocyte-associated protein 4 (CTLA4) KO and anti-CTLA4 upregulated 2-3 m^6^A-RMRs and downregulated one m^6^A-RMR. CD80^+^ programmed cell death 1 ligand 2 (PD-L2)+ (memory B cells) [87] upregulated three and downregulated five m^6^A-RMRs. PD-L1 and anti-PD-L1 upregulated one m^6^A-RMRs and downregulated zero m^6^A-RMRs. T cell immunoglobulin and mucin domain-containing protein 3 (TIM-3) KO, CD3^+^CD8^+^TIM-3^−^ cells [88], and CD8^+^PD1^+^TIM3^−^ cells [89] upregulated 0-5 m^6^A-RMRs and downregulated 2-11 m^6^A-RMRs. T cell immunoreceptor with Ig and ITIM domain- (TIGIT-) Treg upregulated 3 m^6^A-RMRs and downregulated 13 m^6^A-RMRs. In addition to the modulation of m^6^A-RMR expressions by immune checkpoint receptors, the modulation with anti-CD40 (tumor necrosis factor receptor superfamily member 5), a costimulation receptor, resulted in upregulation of 5-12 m^6^A-RMRs and downregulation of 1-2 m^6^A-RMRs. Taken together, these results have demonstrated that immune checkpoint receptors TIM3, TIGIT, PD-L2, and CTLA4 play significant roles in modulating the expressions of m^6^A-RMRs; and inhibition of costimulation receptor CD40 with anti-CD40 antibody significantly upregulates the expressions of m^6^A-RMRs, which is associated with suppression of autoimmune responses by anti-CD40 antibody therapy [90].

### 3.11. Proinflammatory Cytokine Signaling Pathways Significantly Modulate the Expressions of m^6^A-RMRs, and Suppression of Proinflammatory Cytokine Pathways Upregulate More Than Downregulate the Expressions of m^6^A-RMRs

**Figure 13:**
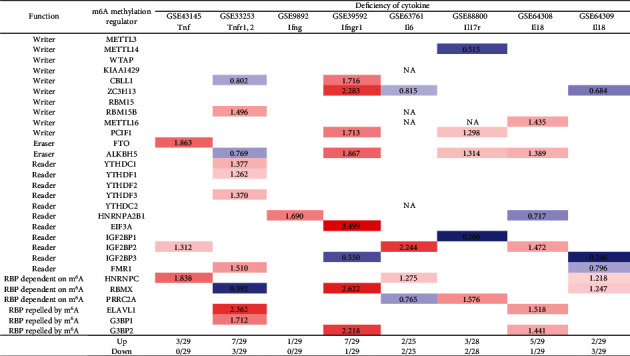
Proinflammatory cytokines can regulate m^6^A-RNA methylation regulators (m^6^A-RMRs). In deficiency of proinflammatory cytokine microarrays, the number of upregulated m^6^A-RMRs is more than that of downregulated m^6^A-RMRs. That is, maybe, proinflammatory cytokines negatively regulate m^6^A methylation. The result is consistent with the result of the m^6^A-RMRs' expression in acute inflammation.

We recently reported that cytokines including IL-17 [91–93], IL-2 [26], and IL-35 [15, 94, 95] play significant roles in inflammation regulation, Treg maintenance, and various pathologies. We hypothesized that signal pathways of cytokines and cytokine receptors regulate the expressions of m^6^A-RMRs. We collected eight cytokine and cytokine receptor microarray datasets from the NIH-NCBI GeoDataset database. As shown in Figure 13, deficiencies of tumor necrosis factor-*α* (TNF*α*, TNF receptor 1-2 (TNFR1-2), interferon-*γ* (IFN*γ*), IFN*γ* receptor-1 (IFN*γ*R1), IL6, IL-17 receptor (IL17R), IL18, and IL18 upregulated 3, 7, 1, 7, 2, 3, 5, and 2 m^6^A-RMRs, respectively, and downregulated 0, 3, 0, 1, 2, 2, 1, and 3 m^6^A-RMRs, respectively. These results have demonstrated that (1) proinflammatory cytokine signaling pathways significantly modulate the expressions of m^6^A-RMRs, and (2) suppression of proinflammatory cytokine pathways upregulates more than downregulates the expressions of m^6^A-RMRs.

### 3.12. NF-*κ*B Components and JAK/STAT Signaling (Except STAT1) Play Important Roles in Upregulating More Than Downregulating m^6^A-RMRs; and Tumor Suppressors TP53, PTEN, and APC Play Significant Roles in Downregulating More Than Upregulating m^6^A-RMRs

**Figure 14:**
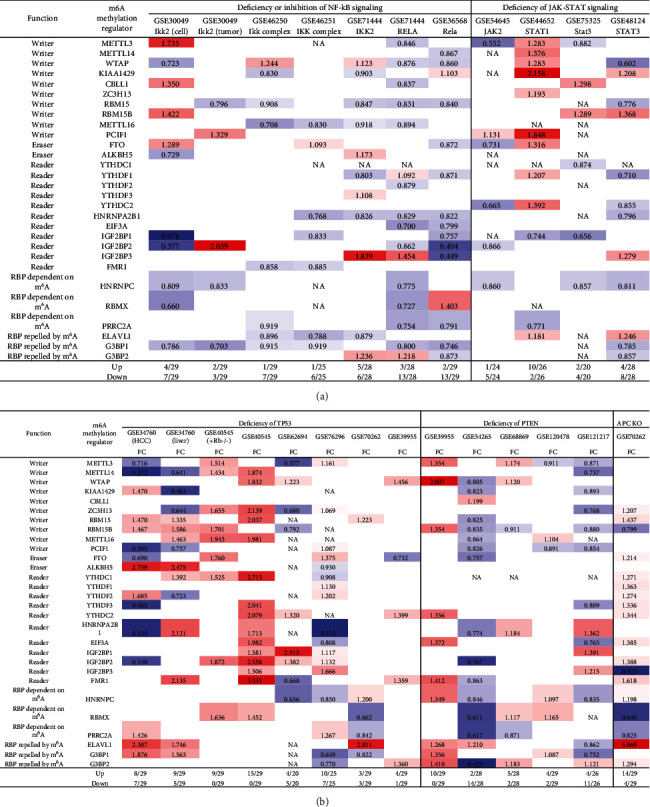
Oncogenes and tumor suppressors can regulate m^6^A-RNA methylation regulators (m^6^A-RMRs). (a) In deficiency of oncogenes, the number of upregulated m^6^A-RMRs is less than that of downregulated m^6^A-RMRs in addition to individual research (GSE44652). (b) In deficiency of tumor suppressors, the number of upregulated m^6^A-RMRs is more than that of downregulated m^6^A-RMRs in addition to individual research. That is, maybe, tumor suppressors negatively regulate m^6^A methylation. These results are consistent with the result of the m^6^A-RMRs' expression in cancers.

We and others reported that proinflammatory/immune regulatory transcription factors (TFs), Janus kinase (JAK)/signal transducer and activator of transcription (STAT) [94], nuclear factor kappa B (NF-*κ*B) [96], tumor protein P53 (TP53), phosphatase and tensin homolog (PTEN), and adenomatous polyposis coli (APC regulator of WNT signaling pathway, APC) [97] play significant roles in regulating inflammation, Treg maintenance, cytokine responses, tumorigenesis, and development. We hypothesized that proinflammatory and immune regulatory TFs, NF-*κ*B, and JAK/STAT pathways and three tumor suppressors TP53, PTEN, and APC pathways regulate the expressions of m^6^A-RMRs. We collected five NF-*κ*B subunit deficiency datasets (seven comparisons), one JAK2 deficiency dataset, one STAT1 deficiency dataset, two STAT3 deficiency datasets, six TP53 deficiency datasets (eight comparisons), five PTEN datasets, and one APC dataset from the NIH-NCBI GeoDataset database. As shown in Figure 14, deficiencies of IKK2 (cell line), IKK2 (tumor), IKK complex, IKK complex, IKK2, RELA, and Rela upregulated 4, 2, 1, 1, 5, 3, and 2 m^6^A-RMRs, respectively, and downregulated 7, 3, 7, 6, 6, 13, and 13, respectively. Of note, deficiencies of NF-*κ*B signaling components resulted in more downregulation than upregulation of m^6^A-RMRs. Deficiencies of JAK2, STAT1, STAT3, and STA3 resulted in upregulating 1, 10, 2, and 4 m^6^A-RMR, respectively, and downregulating 5, 2, 4, and 8 m^6^A-RMRs, respectively. STAT1 deficiency upregulated more than downregulated m^6^A-RMRs, but deficiencies of JAK2 and STAT3 downregulated more than upregulated m^6^A-RMRs. Deficiencies of TP53 in eight datasets upregulated 8, 9, 9, 15, 4, 10, 3, and 4 m^6^A-RMRs, respectively, and downregulated 7, 5, 0, 0, 5, 7, 3, and 1 m^6^A-RMRs, respectively. Of note, six out of 8 TP53-deficient datasets had more upregulation than downregulation of m^6^A-RMR, suggesting that tumor progression triggered by TP53 deficiencies may require more upregulation than downregulation of m^6^A-RMRs. Deficiencies of PTEN in five datasets upregulated 10, 2, 5, 4, and 4 m^6^A-RMRs, respectively, and downregulated 0, 14, 2, 2, and 11 m^6^A-RMRs, respectively. Of note, three out of five PTEN deficient datasets had more upregulation than downregulation of m^6^A-RMR, suggesting that tumor progression triggered by PTEN deficiencies may require more upregulation than downregulation of m^6^A-RMRs. Deficiencies of APC upregulated 14 and downregulated 4 m^6^A-RMRs, respectively. These results have exhibited that (1) NF-*κ*B signaling components play important roles in upregulating more than downregulating m^6^A-RMRs; (2) JAK/STAT signaling pathways play important roles in upregulating more than downregulating m^6^A-RMRs except STAT1; and (3) tumor suppressors TP53, PTEN, and APC play significant roles in downregulating more than upregulating m^6^A-RMRs, suggesting that upregulation of m^6^A-RMRs may favor more than inhibit tumor progression.

### 3.13. Methionine-Homocysteine Cycle Enzyme Mthfd1 Downregulates More Than Upregulates m^6^A-RMRs; One m^6^A Writer RBM15 and One m^6^A Eraser FTO Significantly Modulate the Expressions of m^6^A-RMRs; and H3K4-Specific Methyltransferase MLL1 and DNA Methyltransferase, DNMT1, Significantly Regulate the Expressions of m^6^A-RMRs

**Figure 15:**
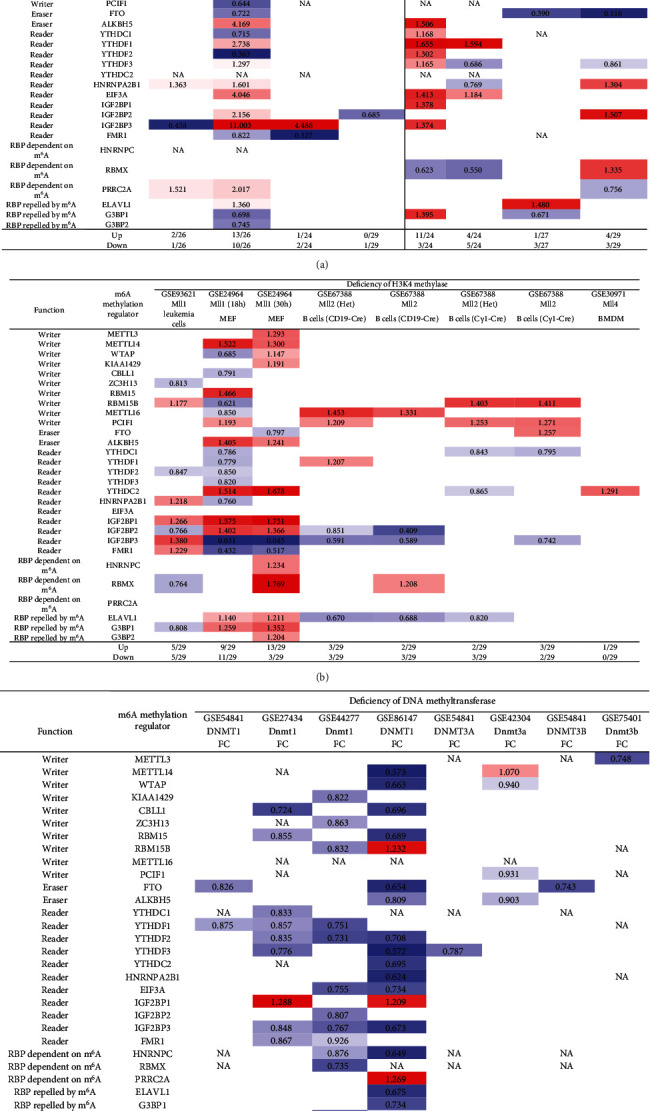
Hyperhomocysteinemia, folate cycle-related enzymes, m^6^A-RNA methylation regulators (m^6^A-RMRs), H3K4 methylases, and DNA methyltransferase regulate the expression of m^6^A-RMRs, suggesting a methylation hierarchy. (a) Hyperhomocysteinemia regulated fewer numbers of m^6^A-RMRs but more m^6^A-RMRs were changed after antihyperhomocysteinemia treatment in hyperhomocysteinemia animal model- (Rattus norvegicus) related microarray (GSE30308). The deficiencies of the folate cycle and metabolism related enzymes and m^6^A-RMRs affect the expression of m^6^A-RMRs. IGF2BP3 was upregulated with fold changes of 4.486, and FMR1 was downregulated with fold changes of 0.327 in the deficiency of methylenetetrahydrofolate Mthfd1. In the deficiencies of m^6^A-RMRs, writer RBM15 and WTAP were downregulated, and CBLL1 and YTHDF1 were upregulated. WTAP, HNRNPA2B1, IGF2BP2, ELAVL1, and RBMX were upregulated in the deficiency of FTO in thoracic aortae. (b) In mammals, SET1a and SET1b and MLL1–MLL4 are the main six enzymes that catalyze strimethylation of lysine 4 in histone H3 (H3K4). The deficiencies of MLL1, MLL2, and MLL4 can regulate the expression change of m^6^A-RMRs; regulation of m^6^A-RMRs by MLL1 is different in a circadian study (GSE24964); upregulated m^6^A-RMRs were increased in 30 hours compared to 18 hours in deficiency of MLL1; regulation of m^6^A-RMRs by the heterozygote of MLL2 is similar to the deficiency of homozygous (GSE67388); deficiency of homozygous MLL4 only regulates one of the 29 m^6^A-RMRs compared with heterozygous MLL4 (GSE30971). (c) In deficiency of DNA methyltransferases microarrays, the number of downregulated m^6^A-RMRs is more than that of upregulated m^6^A-RMRs. That is, maybe, DNA methyltransferases positively regulate m^6^A methylation. More differentially expressed m^6^A-RMRs in microarrays with deficiencies of DNMT1 are more than those in microarrays with DNMT3A or DNMT3B. Most m^6^A-RMRs (18/26) are differentially expressed, and the downregulated genes (*n* = 15) are more than the upregulated genes (*n* = 3) in DNMT1 knockdown myeloma cells (GSE86147).

We reported that hyperhomocysteinemia promotes differentiation of the inflammatory Ly6C^+^ monocyte [5, 98, 99], decreased high-density lipoprotein (HDL) [100, 101], endothelial cell activation, and programmed cell death [6] and atherosclerosis [102]. We hypothesized that m^6^A-RMRs, histone lysine methylases, DNA methyltransferases, and methyl donating homocysteine-methionine metabolism cycle play significant roles in regulating the expressions of m^6^A-RMRs as a feedback mechanism. To examine this hypothesis, we collected numerous microarray datasets including a methionine diet-fed model, Mthfd1 KO, Mthfr KO, eight histone 3 lysine 4 (H3K4) methylase KOs, and eight DNA methyltransferase KOs from the NIH-NCBI-GeoDataset database. As shown in Figure 15(a), the methionine diet-fed model upregulated three m^6^A-RMRs and downregulated one m^6^A-RMR; deficiencies of methylenetetrahydrofolate dehydrogenase (Mthfd1) and methylenetetrahydrofolate reductase (Mthfr) resulted in upregulation of one and zero m^6^A-RMR, respectively, and downregulation of two and one m^6^A-RMRs, respectively. Deficiencies of m^6^A-RMRs RBM15 and FTO upregulated 1 to 11 m^6^A-RMRs and downregulated 3-5 m^6^A-RMRs. As shown in Figure 15(b), deficiencies of histone lysine 4 methylase in eight datasets upregulated one to 13 m^6^A-RMRs and downregulated zero to 11 m^6^A-RMRs. Among the eight comparison datasets, three datasets of deficiencies of H3K4-specific methyltransferase MLL1 [103] modulated m^6^A-RMRs the most, with 10 out of 29, 20 out of 29, and 16 out of 29 m^6^A-RMRs, respectively. Moreover, deficiencies of DNA methyltransferase 1 (DNMT1) in GSE54841, GSE27434, GSE44277, and GSE86147; DNMT3A at GSE54841 and GSE42304; and DNMT3B at GSE54841 and GSE75401 upregulated 0, 1, 0, 3, 0, 1, 0, and 0 m^6^A-RMRs, respectively, and downregulated 2, 8, 12, 15, 1, 3, 1, and 1 m^6^A-RMRs, respectively. Among the eight datasets of DNA methyltransferase deficiencies, DNMT1 deficiencies modulated the most with 9, 12, and 18 m^6^A-RMRs except GSE54841 (Figure 15(c)). Taken together, these results have demonstrated that (1) methionine diet-fed model and methionine-homocysteine cycle enzyme Mthfr do not significantly regulate the expressions of m^6^A-RMRs but methionine-homocysteine cycle enzyme Mthfd1 downregulates more than upregulates m^6^A-RMRs; (2) one m^6^A writer RBM15 and one eraser FTO significantly modulate the expressions of m^6^A-RMRs, suggesting a feedback mechanism for regulation of m^6^A-RMR expressions; (3) H3K4-specific methyltransferase MLL1 significantly regulates the expressions of m^6^A-RMRs; and (4) DNA methyltransferase, DNMT1, modulates the expressions of m^6^A-RMRs.

### 3.14. The 18 Out of 165 ROS Regulators (11%) Were Modulated by Four Human m^6^A Writers KIAA1429, METTL14, METTL3, and WTAP, and Gene KO Data Showed That 40 Out of 165 ROS Regulators (24%) Were Modulated by m^6^A Eraser FTO and Two m^6^A Writers METTL3 and WTAP

Our and others' reports showed that reactive oxygen species (ROS) play significant roles not only in mediating endothelial cell activation and vascular inflammation [104] but also in sensing metabolic homeostasis and stress in organelle metabolic process as an integrated system. An important question remained whether m^6^A-RMRs regulate ROS regulators. We hypothesized that m^6^A-RMRs regulate the expressions of ROS regulators. To examine this hypothesis, the ROS regulators were the targets of RNA methylation regulators in human cell studies as shown in Table 5. The TREW data (Target of m^6^A Readers, Erasers, and Writers) was download from Met-DB v2.0 (MeT-DB V2.0: the MethylTranscriptome DataBase Version 2.0 http://180.208.58.19/metdb_v2/html/index.php) [105]. The 165 ROS regulators were examined as we reported. The result showed that 18 out of 165 ROS regulators (11%) (F2RL1, PDK4, TIGAR, BCL2, SESN2, GNAI2, DDIT4, SH3PXD2A, FOXM1, AATF, TGFB1, TSPO, G6PD, GNAI3, and CYP1B1) were modulated by four m^6^A writers KIAA1429, METTL14, METTL3, and WTAP (several ROS regulators were modulated in more than one position in chromosome or by more than one RNA methylation regulators) (Table 5). The deficiencies of four m^6^A writers (KIAA1429, METTL14, METTL3, and WTAP) downregulated the m^6^A modification of ROS regulators. As shown in Supplementary Table 4, the expressions of ROS regulators were also the targets of RNA methylation regulators in mouse cell studies. TREW (Target of m^6^A Readers, Erasers and Writers) was download from Met-DB v2.0 (MeT-DB V2.0: the MethylTranscriptome DataBase Version 2.0 http://180.208.58.19/metdb_v2/html/index.php) [105]. The 165 ROS regulators were examined; and the result showed that 40 out of 165 ROS regulators (24%) were modulated by m^6^A eraser FTO and two m^6^A writers METTL3 AND WTAP. The deficiency of m^6^A eraser FTO upregulated the m^6^A modification of ROS regulators, and the deficiencies of two m^6^A writers METTL3 or WTAP downregulated the m^6^A modification of ROS regulators.

## 4. Discussion

Recent progress has reported that m^6^A-RNA methylation [50] plays a significant role in regulating cardiovascular diseases (CVD) and CVD-related diseases and complications such as cardiac remodeling, atherosclerosis, heart failure, inflammation adipogenesis, obesity, insulin resistance, hypertension, and type 2 diabetes mellitus [41]. In addition, m^6^A-RNA methylation plays critical roles in the pathogenesis of cancers and tumors [106, 107], aging [107], immune responses and autoimmunity, and viral infections [108, 109]. However, two major issues remain unknown: first, transcriptomic regulation of a complete list of m^6^A-RNA methylation regulators in various diseases and second, cellular mechanisms and molecular mechanisms underlying transcriptomic changes of m^6^A-RNA methylation regulators in pathophysiological conditions. To solve these problems, we performed a transcriptomic data mining of the expressions of 29 m^6^A-RNA methylation regulators in diseases and cancers and made significant findings: (1) a few m^6^A-RMRs were upregulated; and most m^6^A-RMRs were downregulated in sepsis, acute respiratory distress syndrome, shock, and trauma; (2) half of 29 m^6^A-RMRs were downregulated in atherosclerosis progression compared with those of regression; (3) IBD and RA modulated m^6^A-RMRs more than lupus and psoriasis; and some autoimmune diseases share five upregulated m^6^A-RMRs; (4) some organ failures shared eight upregulated m^6^A-RMRs; end-stage renal failure (ESRF) downregulated 85% of m6A-RMRs; and upregulation of m^6^A-RMRs in hemodialysis more than in ESRF may have clinical benefits; (5) MERS-CoV infections modulated m^6^A-RMRs the most among viral infections; (6) oxPAPC and NOTCH1 knockdown modulated m^6^A-RMRs more than other DAMs stimulation of endothelial cells including LPS, oxLDL, and IFNs; (7) upregulated m^6^A-RMRs were more than downregulated m^6^A-RMRs in cancer types; five types of cancers upregulated ≥10 m^6^A-RMRs; (8) M1 macrophages upregulated seven m^6^A-RMRs; WTAP and IGF2BP2 may not only promote M1 but also inhibit M2 polarization; (9) 86% of m^6^A-RMRs were differentially expressed in the six spleen Treg clusters; and 8 out of 12 Treg signatures significantly regulated m^6^A-RMRs; (10) immune checkpoint receptors TIM3, TIGIT, PD-L2, and CTLA4 significantly modulated m^6^A-RMRs; and inhibition of costimulation receptor CD40 with anti-CD40 significantly upregulated m^6^A-RMRs; (11) proinflammatory cytokines significantly modulated m^6^A-RMRs; (12) NF-*κ*B and JAK/STAT pathways (except STAT1) upregulated more than downregulated m^6^A-RMRs; and TP53, PTEN, and APC downregulated more than upregulated m^6^A-RMRs; (13) methionine-homocysteine cycle enzyme Mthfd1 downregulated more than upregulated m^6^A-RMRs; m^6^A writer RBM15 and one m^6^A eraser FTO significantly modulated m^6^A-RMRs; and H3K4 methyltransferase MLL1 and DNA methyltransferase, DNMT1, significantly regulated m^6^A-RMRs; and (14) 40 out of 165 ROS regulators were modulated by m^6^A eraser FTO and two m^6^A writers METTL3 and WTAP.

For some diseases, both eraser and writing enzymes of m^6^A-RMRs have changed or have the same expression trend. In order to explain this phenotype, we checked the m^6^A-RMRs changes in Met-DB v2.0 and the result supports m^6^A-RMRs regulate expression of m^6^A-RMRs themselves (Supplementary Tables 5 and 6). When the writer METTL3 or WTAP was knocked down, the expression of eraser ALKBH5 also was down regulated (Supplementary Table 5). Then, Protein-Protein Interaction (PPI) analysis was performed by using STRING (https://string-db.org/) and the result also suggest the interactions among m^6^A-RMRs are complex (Supplementary Figure 3). Additionally, the expression of two kinds of m^6^A-RMRs such as writer WTAP and eraser FTO is positively correlated in some tumors (Supplementary Figure 4). So, in some diseases, writers and erasers both are upregulated or downregulated, which is reasonable and the main function of m^6^A-RMRs can be confirmed by using some well-designed experiments.

One of the potential issues related to database mining is that we were unable to compare the impact of different regulators in controlling the expressions of m^6^A-RMRs in the same cell types since the original microarray studies we looked at employed different cells. Although our database mining strategy was not optimal, our approach was justified in filling up a critical knowledge gap. This is, in fact, a common practice that we and others [110] often use in studying gene expression in nonideal, heterogeneous peripheral blood mononuclear cell populations (PBMCs) in disease conditions versus healthy conditions, and PBMCs are actually made up of a variety of cell types, including B cells (~15%), T cells (~70%), monocytes (~5%), and natural killer (NK) cells (~10%) among others [111]. Another limitation of the current study is that, due to the low-throughput nature of verification techniques in every laboratory, including ours, we were unable to confirm every result we uncovered using high-throughput data analyses. We recognize that in the future, carefully designed in vitro and in vivo experimental models will be required to confirm regulator gene deficiency-upregulated m6A-RMRs further and the underlying mechanisms we disclose here.

**Figure 16:**
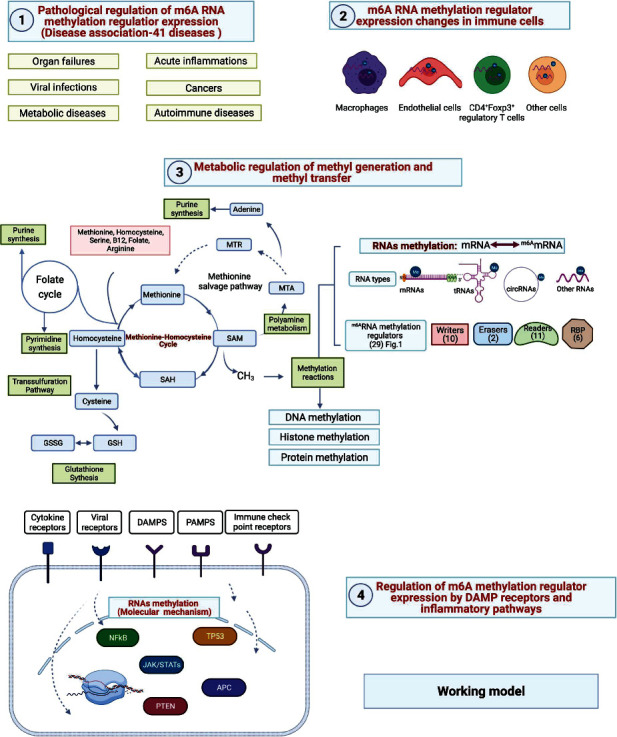
A novel working model was proposed in our study. (1) M^6^A-RMRs differently expressed in acute inflammations, metabolic diseases, autoimmune diseases, organ failures, viral infections, and tumors. (2) Cellular mechanisms such as macrophage polarization, endothelial cell activation, CD4^+^Foxp3^+^ regulatory T cell activation, and resting status, and pathophysiological changes of other cells regulate the transcriptomic changes of m^6^A-RMRs. (3) In folate cycle, transsulfuration pathway, glutathione synthesis, polyamine metabolism, and methionine salvage pathway, homocysteine-methionine cycle serves as a sensor-receptor system to sense the intracellular metabolic homeostasis and stresses of four amino acids such as methionine, homocysteine, serine, and arginine as well as vitamin B12 and folate. The metabolic homeostasis and stress signals relay the metabolic reprogramming signals into cellular methylation processes via various methyltransferases to methylate DNAs, proteins, histones, RNAs, and other molecules. (4) Cell surface receptors such as cytokine receptors, viral receptors, danger-associated molecular pattern (DAMPs) receptors/pathogen-associated molecular pattern (PAMPs) receptors, immune checkpoint receptors, and cosignaling receptors regulate the transcriptomic changes of m^6^A-RMRs. Nuclear transcription factors (TFs) including proinflammatory TFs NF-*κ*B, Jak-STATs, tumor suppressors TP53, PTEN, and APC regulate the transcriptomic changes of m^6^A-RMRs. The figure was created with http://BioRender.com.

Based on our findings, we proposed a novel working model in Figure 16. First, we recently proposed a new theory that because of their connections with three metabolic pathways including folate cycle, transsulfuration pathway, glutathione synthesis, polyamine metabolism, and methionine salvage pathway, homocysteine-methionine cycle serves as a sensor-receptor system to sense the intracellular metabolic homeostasis and stresses of four amino acids such as methionine, homocysteine, serine, and arginine as well as vitamin B12 and folate; second, similar to protein phosphorylation/dephosphorylation-based signaling pathways, the metabolic homeostasis and stress signals relay the metabolic reprogramming signals into cellular methylation processes via various methyltransferases to methylate DNAs, proteins, histones, RNAs, and other molecules. The methylations of those important molecules regulate their biological functions; third, m^6^A-RNA methylation is a dominant RNA methylation for various RNA types including mRNAs, tRNAs, rRNAs, and noncoding RNAs. Throughout transcriptomic data analyses of 102 microarrays, RNA-Seq, and single-cell RNA-Seq related to 41 diseases in six categories organ failures, viral infections, metabolic diseases, acute inflammations, cancers, and autoimmune diseases, our data have demonstrated that several layers of regulatory systems regulate the transcriptomic changes of m^6^A-RMRs in diseases as well as pathophysiological conditions in various cell types, which include (1) cell surface receptors such as cytokine receptors, viral receptors, danger-associated molecular pattern (DAMP) receptor, pathogen-associated molecular pattern (PAMP) receptors, immune checkpoint receptors, and cosignaling receptors. Of note, immune checkpoint receptors and cosignaling receptors are the prototypic membrane protein interactions between cells; (2) cellular mechanisms such as macrophage polarization, endothelial cell activation, CD4^+^Foxp3^+^ regulatory T cell activation, resting status, and pathophysiological changes of other cells; and (3) nuclear transcription factors (TFs) including proinflammatory TFs NF-*κ*B, Jak-STATs, and tumor suppressors TP53, PTEN, and APC. In summary, our results have demonstrated that transcriptional regulations of m^6^A-RMRs are highly significant mechanisms in regulating m^6^A-RNA methylations related to various pathophysiological processes and diseases. Our findings provide novel insights on the roles of m^6^A-RMRs in the development of inflammatory disorders and malignancies as well as novel pathways for future therapeutic strategies for inflammatory diseases, sepsis, trauma, organ failures, autoimmune diseases, metabolic CVDs, and cancers.

## Figures and Tables

**Table 1:** List of microarray datasets (23) of acute inflammations, metabolic diseases, autoimmune diseases, and organ failures in the NIH-NCBI-GeoDataset database (https://www.ncbi.nlm.nih.gov/gds/) we collected in this study.

GEO ID	Disease/phenotype	Tissue	Comparison	No. of samples	PMID
Acute inflammation					
GSE32707	Lung injury	Whole blood	Sepsis day 0 vs. no sepsis	30/34	22461369
	Lung injury	Whole blood	Sepsis day 7 vs. no sepsis	28/34	22461369
	Lung injury	Whole blood	Sepsis/ARDS day 0 vs. no sepsis ARDS	18/34	22461369
	Lung injury	Whole blood	Sepsis/ARDS day 7 vs. no sepsis ARDS	13/34	22461369
GSE13904	Septic shock	Whole blood	Sepsis day 1 vs. normal	32/18	19325468
	Septic shock	Whole blood	Sepsis day 3 vs. normal	20/18	19325468
	Septic shock	Whole blood	Sepsis/shock day 1 vs. normal	67/18	19325468
	Septic shock	Whole blood	Sepsis/shock day 3 vs. normal	39/18	19325468
GSE5580	Severe trauma	Monocytes	Severe trauma vs. health	7/7	17032758
	Severe trauma	Leukocytes	Severe trauma vs. health	7/7	17032758
	Severe trauma	T cells	Severe trauma vs. health	7/7	17032758
Metabolic disease					
GSE48964	Obese	Adipose stem cells	Morbidly obese vs. nonobese	3/3	24040759
GSE55200	MHO	Subcutaneous adipose	MHO vs. LH	8/7	24933025
	MUO	Subcutaneous adipose	MUO vs. LH	8/7	24933025
GSE94752	Obese IR	Adipocytes	Obese IR vs. lean	18/9	28570579
	Obese IS	Adipocytes	Obese IS vs. lean	21/9	28570579
GSE23343	T2D	Liver	T2D vs. normal glucose tolerance	10/7	21035759
GSE29221	T2D	Skeletal muscle	T2D vs. nondiabetes	12/12	23308243
GSE29226	T2D	Subcutaneous adipose	T2D vs. nondiabetes	12/12	23308243
GSE29231	T2D	Visceral adipose	T2D vs. nondiabetes	12/12	23308243
GSE28829	Atherosclerosis	Carotid artery	Advanced plaque vs. early plaque	16/13	22388324
GSE41571	Atherosclerosis	Plaque macrophages	Ruptured plaques vs. stable plaque	5/6	23122912
GSE1010	FCH	Lymphoblastic cells	FCH vs. normal	12/12	15388524
GSE6054	FHC and atherosclerosis	Monocytes	FHC homozygote vs. control	4/13	19040724
GSE6088	FHC and atherosclerosis	T cell	FHC homozygote vs. control	3/13	19040724
Autoimmune disease					
GSE97779	RA	Macrophages^∗^	RA vs. normal	9/5	28813657
GSE109248	ACLE	Skin	ACL vs. normal	7/14	29889098
	CCLE	Skin	CCL vs. normal	6/14	29889098
	Psoriasis	Skin	Psoriasis vs. normal	17/14	29889098
	SCLE	Skin	SCL vs. normal	12/14	29889098
GSE38713	UC	Sigmoid colon or rectum	UC active involved vs. normal	22/13	23135761
GSE3365	UC	PBMC	UC vs. normal	26/42	16436634
	CD	PBMC	CD vs. normal	59/42	16436634
Organ failure					
GSE76701	Heart failure	Left ventricle	Failing heart vs. nonfailing heart	4/4	26756417
GSE38941	HBV-ALF	Liver	HBV-ALF vs. normal	17/10	23185381
GSE37171	ESRF	Whole blood	Chronic renal failure vs. healthy controls	75/40	23809614
GSE15072	CKD hemodialysis	PBMC	Hemodialysis vs. healthy controls	17/8	19698090

Abbreviations: No. of samples: number of samples = (number of diseases/number of controls); ARDS: acute respiratory distress syndrome; ALI: acute liver injury; ALF: acute liver failure; PBMC: peripheral blood mononuclear cell; IS: insulin sensitive; IR: insulin resistance; MHO: metabolically healthy obese; MUO: metabolically unhealthy obese; LH: lean health; T2D: type 2 diabetes; FCH: familial combined hyperlipidemia; FHC: familial hypercholesterolemia; RA: rheumatoid arthritis; ACLE: acute cutaneous lupus; CCLE: chronic cutaneous lupus; SCLE: subacute cutaneous lupus; UC: ulcerative colitis; CD: Crohn's disease; HBV-ALF: hepatitis B virus-associated acute liver failure; ESRF: end-stage renal failure; CKD: chronic kidney disease; PBMC: peripheral blood mononuclear cell, macrophages; ^∗^macrophages from synovial fluids for RA and blood-derived macrophages for control. All the studies and samples are from human.

**Table 2:** Three microarray datasets of respiratory virus infections with time course including Middle-East respiratory syndrome (MERS) coronavirus, severe acute respiratory syndrome (SARS) coronavirus, and avian influenza virus infection in the NIH-NCBI-GeoDataset database (https://www.ncbi.nlm.nih.gov/gds/) were collected to analyze the expression changes of m^6^A-RNA methylation regulators.

GEO ID	Comparison	Cell/tissue	No. of samples	PMID
GSE79218	icMERS-inoculated vs. mock-inoculated (0 hour)	HMEC	5/5	28830941
	icMERS-inoculated vs. mock-inoculated (12 hours)	HMEC	5/5	28830941
	icMERS-inoculated vs. mock-inoculated (24 hours)	HMEC	5/5	28830941
	icMERS-inoculated vs. mock-inoculated (36 hours)	HMEC	5/5	28830941
	icMERS-inoculated vs. mock-inoculated (48 hours)	HMEC	5/4	28830941

GSE47960	SARS-CoV-infected vs. mock-infected (0 hour)	HAEC	4/3	23935999
	SARS-CoV-infected vs. mock-infected (12 hours)	HAEC	4/3	23935999
	SARS-CoV-infected vs. mock-infected (24 hours)	HAEC	4/3	23935999
	SARS-CoV-infected vs. mock-infected (36 hours)	HAEC	2/3	23935999
	SARS-CoV-infected vs. mock-infected (48 hours)	HAEC	4/3	23935999
	SARS-CoV-infected vs. mock-infected (60 hours)	HAEC	4/4	23935999
	SARS-CoV-infected vs. mock-infected (72 hours)	HAEC	4/4	23935999
	SARS-CoV-infected vs. mock-infected (84 hours)	HAEC	4/4	23935999
	SARS-CoV-infected vs. mock-infected (96 hours)	HAEC	4/4	23935999
	H1N1-infected vs. mock-infected (0 hour)	HAEC	3/3	23935999
	H1N1-infected vs. mock-infected (6 hours)	HAEC	3/3	23935999
	H1N1-infected vs. mock-infected (12 hours)	HAEC	3/3	23935999
	H1N1-infected vs. mock-infected (18 hours)	HAEC	3/3	23935999
	H1N1-infected vs. mock-infected (24 hours)	HAEC	3/3	23935999
	H1N1-infected vs. mock-infected (36 hours)	HAEC	3/3	23935999
	H1N1-infected vs. mock-infected (48 hours)	HAEC	4/3	23935999

GSE49840	H7N9-infected vs. mock-infected (3 hours)	Calu-3 cells	4/4	24496798
	H7N9-infected vs. mock-infected (7 hours)	Calu-3 cells	4/4	24496798
	H7N9-infected vs. mock-infected (12 hours)	Calu-3 cells	4/4	24496798
	H7N9-infected vs. mock-infected (24 hours)	Calu-3 cells	4/4	24496798
	H7N7-infected vs. mock-infected (3 hours)	Calu-3 cells	4/4	24496798
	H7N7-infected vs. mock-infected (7 hours)	Calu-3 cells	4/4	24496798
	H7N7-infected vs. mock-infected (12 hours)	Calu-3 cells	4/4	24496798
	H7N7-infected vs. mock-infected (24 hours)	Calu-3 cells	4/4	24496798
	H5N1-infected vs. mock-infected (3 hours)	Calu-3 cells	3/4	24496798
	H5N1-infected vs. mock-infected (7 hours)	Calu-3 cells	4/4	24496798
	H5N1-infected vs. mock-infected (12 hours)	Calu-3 cells	4/4	24496798
	H5N1-infected vs. mock-infected (24 hours)	Calu-3 cells	4/4	24496798
	H3N2-infected vs. mock-infected (3 hours)	Calu-3 cells	34	24496798
	H3N2-infected vs. mock-infected (7 hours)	Calu-3 cells	4/4	24496798
	H3N2-infected vs. mock-infected (12 hours)	Calu-3 cells	4/4	24496798
	H3N2-infected vs. mock-infected (24 hours)	Calu-3 cells	4/4	24496798

Abbreviations: No. of samples: number of samples = (number of diseases/number of controls); MERS: Middle East respiratory syndrome; SARS: severe acute respiratory syndrome; H1N1: influenza A, one type of avian influenza virus.

**Table 3:** Eight microarray datasets about endothelial cells in different conditions in the NIH-NCBI-GeoDataset database (https://www.ncbi.nlm.nih.gov/gds/) were collected to analyze the changes of m^6^A methylation regulators.

GEO ID	Comparison	Cell/tissue	No. of samples	PMID
GSE59226	Influenza virus-infected vs. inactivate virus-infected	Human umbilical vein ECs	2/2	25863179

GSE1377	Kaposi sarcoma-associated herpes virus-infected for 7 days vs. uninfected control	Primary human dermal ECs	2/2	15220917

GSE5883	10 ng LPS stimulation for 4 hours vs. no LPS stimulation	Human lung microvascular ECs	4/4	NA
	10 ng LPS stimulation for 8 hours vs. no LPS stimulation	Human lung microvascular ECs	4/4	NA
	10 ng LPS stimulation for 24 hours vs. no LPS stimulation	Human lung microvascular ECs	4/4	NA

GSE3920	1000 IU IFN*α* treated for 5 hours vs. untreated control	Human umbilical vein ECs	5/5	17202376, 19553003
	1000 IU IFN*β* treated for 5 hours vs. untreated control	Human umbilical vein ECs	2/5	17202376, 19553003
	1000 IU IFN*γ* treated for 5 hours vs. untreated control	Human umbilical vein ECs	3/5	17202376, 19553003

GSE85987	NOTCH1 siRNA vs. scrambled control siRNA	Human umbilical vein ECs	3/3	29449332
	NOTCH1 siRNA+IL-1*β* treated 24 hours vs. scrambled siRNA	Human umbilical vein ECs	3/3	29449332

GSE72633	NOTCH1 siRNA vs. scrambled control siRNA	Human aortic ECs	3/3	26552708
	oxPAPC treated vs. scrambled control siRNA	Human aortic ECs	3/3	26552708

GSE26953	Oscillatory shear vs. laminar shear (fibrosa)	Human aortic valvular ECs	6/6	21705672
	Oscillatory shear vs. laminar shear (ventricularis)	Human aortic valvular ECs	6/6	21705672

GSE39264	apoE KO vs. WT	Mouse aortic ECs	4/6	23990205
	LPS treated for 4 hours vs. DMEM treated for 4 hours	Mouse aortic ECs	3/3	23990205
	oxLDL treated for 4 hours vs. DMEM treated for 4 hours	Mouse aortic ECs	3/3	23990205
	oxPAPC treated for 4 hours vs. DMEM treated for 4 hours	Mouse aortic ECs	3/3	23990205

Abbreviations: LPS: lipopolysaccharide; IFN: interferon; NOTCH1: notch receptor 1; ox-PAPC: oxidized-1-palmitoyl-2-arachidonyl-sn-glycerol-3-phosphocholine; siRNA: small interfering RNA; apoE: apolipoprotein E; KO: knockout; WT: wild type; ox-LDL: oxidized low-density lipoprotein; DMEM: Dulbecco's modified eagle medium; No. of samples: number of samples = (number of diseases/number of controls).

**Table 4:** Comparison of our study with other three studies about cardiovascular disease.

m^6^A-RMRs	Results in our study (Figure 4(a))	Table 1 in PMID: 34489709	Table 1 in PMID:34221238	Table 1 in PMID: 32910911
	M^6^A-RMRs in atherosclerosis	RNA methylation-related enzymes in CVDs	Roles and mechanisms of RNA m^6^A effectors in CVDs	The association between m^6^A methylation and CVDs
Upregulated genes	PCIF1, YTHDC2, IGF2BP2, IGF2BP3, HNRNPC, PRRC2A, ELAVL1	METTL3, YTHDF2, METTL14, KIAA1429,	N/A	METTL14
Downregulated genes	METTL14, KIAA1429,ZC3H13, FTO, YTHDF3, HNRNPA2B1, FMR1, G3BP1, G3BP2	FTO	ALKBH5, YTHDF2	FTO
Genes without expression changes	METTL3, CBLL1, RBM15B, YTHDF1, YTHDF2, EIF3A, IGF2BP1	N/A	N/A	N/A
Expression of genes not known	METTL16, YTHDC1, RBMX	N/A	N/A	N/A
Uncertain (paradox)	WTAP, RBM15, ALKBH5	YTHDF1	METTL3, FTO, WTAP	METTL3

Abbreviations: CVDs: cardiovascular diseases; N/A: not available.

**Table 5:** The reactive oxygen species (ROS) regulators were the targets of RNA methylation regulators in human cell studies. TREW (Target of m^6^A Readers, Erasers, and Writers) was downloaded from Met-DB v2.0 (MeT-DB V2.0: the Methyl Transcriptome DataBase Version 2.0 http://180.208.58.19/metdb_v2/html/index.php PMID: 29126312). 165 ROS regulators were examined, and the result showed 18 ROS regulators (F2RL1, PDK4, TIGAR, BCL2, SESN2, GNAI2, DDIT4, SH3PXD2A, FOXM1, AATF, TGFB1, TSPO, G6PD, GNAI3, and CYP1B1) were modulated by writers KIAA1429, METTL14, METTL3, and WTAP (several ROS regulators were modulated in more than one position in the chromosome or by more than one RNA methylation regulator). The deficiencies of writers (KIAA1429, METTL14, METTL3, and WTAP) can downregulate the m6A modification of ROS regulators (*p* adj < 0.05).

Targeted-ROS regulator	Type	m6A methyl-regulator	*p* adj	p_treated	p_control	Log2_OR	Log2_RR	*q*	Experiment (modification-RNA methylation regulators-cell)	PMID
F2RL1	Writer	KIAA1429	0.01	0.35	0.59	-1.39	-0.74	893.65	m6A-KIAA1429-si-A549	24981863
F2RL1	Writer	KIAA1429	0.02	0.46	0.75	-1.86	-0.72	1959.11	m6A-KIAA1429-si-A549	24981863
PDK4	Writer	KIAA1429	0.02	0.11	0.69	-4.12	-2.60	91.22	m6A-KIAA1429-si-A549	24981863
TIGAR	Writer	KIAA1429	0.04	0.19	0.73	-3.56	-1.95	309.32	m6A-KIAA1429-si-A549	24981863
BCL2	Writer	METTL14	0.03	0.63	0.95	-3.37	-0.58	130.91	m6A-METTL14-sh2-A549	24981863
SESN2	Writer	METTL3	0.03	0.25	0.46	-1.35	-0.88	80.55	m6A-METTL3-kd-Hela	24316715
GNAI2	Writer	METTL3	≤0.01	0.26	0.41	-0.99	-0.66	474.67	m6A-METTL3-kd-Hela	24316715
GNAI2	Writer	METTL3	≤0.01	0.26	0.41	-0.99	-0.66	474.67	m6A-METTL3-kd-Hela	24316715
DDIT4	Writer	METTL3	≤0.01	0.27	0.44	-1.05	-0.68	1549.26	m6A-METTL3-kd-Hela	24316715
DDIT4	Writer	METTL3	≤0.01	0.27	0.44	-1.05	-0.68	1549.26	m6A-METTL3-kd-Hela	24316715
DDIT4	Writer	METTL3	≤0.01	0.27	0.44	-1.05	-0.68	1549.26	m6A-METTL3-kd-Hela	24316715
SH3PXD2A	Writer	METTL3	0.03	0.55	0.60	-0.29	-0.13	2889.11	m6A-METTL3-kd-Hela	24316715
SH3PXD2A	Writer	METTL3	0.03	0.55	0.60	-0.29	-0.13	2889.11	m6A-METTL3-kd-Hela	24316715
SH3PXD2A	Writer	METTL3	0.03	0.55	0.60	-0.29	-0.13	2889.11	m6A-METTL3-kd-Hela	24316715
SH3PXD2A	Writer	METTL3	0.03	0.55	0.60	-0.29	-0.13	2889.11	m6A-METTL3-kd-Hela	24316715
FOXM1	Writer	METTL3	0.02	0.33	0.40	-0.43	-0.27	427.19	m6A-METTL3-kd-Hela	24316715
AATF	Writer	METTL3	≤0.01	0.19	0.39	-1.40	-1.00	132.56	m6A-METTL3-kd-Hela	24316715
TGFB1	Writer	METTL3	≤0.01	0.46	0.60	-0.79	-0.37	1205.15	m6A-METTL3-kd-Hela	24316715
TSPO	Writer	METTL3	0.01	0.25	0.37	-0.78	-0.54	186.38	m6A-METTL3-kd-Hela	24316715
G6PD	Writer	METTL3	≤0.01	0.36	0.40	-0.28	-0.17	2992.01	m6A-METTL3-kd-Hela	24316715
GNAI3	Writer	WTAP	0.02	0.17	0.46	-2.01	-1.41	154.65	m6A-WTAP-kd-Hela	24316715
GNAI3	Writer	WTAP	0.02	0.17	0.46	-2.01	-1.41	154.65	m6A-WTAP-kd-Hela	24316715
TIGAR	Writer	WTAP	0.01	0.19	0.56	-2.42	-1.54	104.56	m6A-WTAP-kd-Hela	24316715
CYP1B1	Writer	WTAP	0.02	0.50	0.95	-4.33	-0.93	583.55	m6A-WTAP-si-A549	24981863
DDIT4	Writer	WTAP	≤0.01	0.46	0.73	-1.66	-0.66	825.45	m6A-WTAP-si-Hek293T	24981863

Met-DB v2.0 contains a significant increase in context-specific m^6^A peaks and single-base sites predicted from 185 samples from 26 separate studies for 7 species. It has also been updated to include a new database for targets of m^6^A readers, erasers, and writers, as well as additional functional data gathering. The abbreviation TREW stands for Target of m^6^A Readers, Erasers, and Writers. To discover their target sits, we collected ParCLIP-seq and MeRIP-seq data for 8 regulator/reader proteins (including FTO, KIAA1429, METTL14, METTL3, WTAP, HNRNPC, YTHDC1, and YTHDF1) from 10 independent studies. Then, the differential m6A peaks that showed significant hypermethylation (hypomethylation) after knocking down of a demethylase (methylase) were determined to the target peaks. p_treat: peak of treated group; p_control: peak of control group; OR: odds ratio; RR: relative risk or risk ratio.

## Data Availability

All the datasets used in this study are publicly available. The analyzed results in this study are included within the article and Supplementary Materials.

## Supplementary Materials

Supplementary Materials See Table S1 to S4 and Figures S1-S2 in the supplementary material for comprehensive data analysis.
