# Supplementary material for: 29 m6A-RNA Methylation (Epitranscriptomic) Regulators Are Regulated in 41 Diseases including Atherosclerosis and Tumors Potentially via ROS Regulation – 102 Transcriptomic Dataset Analyses
Source: J Immunol Res. 2022 Feb 15;2022:1433323. doi: 10.1155/2022/1433323 (PMC8863469; doi:10.1155/2022/1433323)
Supplement: Supplementary Materials — See Table S1 to S4 and Figures S1-S2 in the supplementary material for comprehensive data analysis. [file 1433323.f1.pptx]

## Slide 1
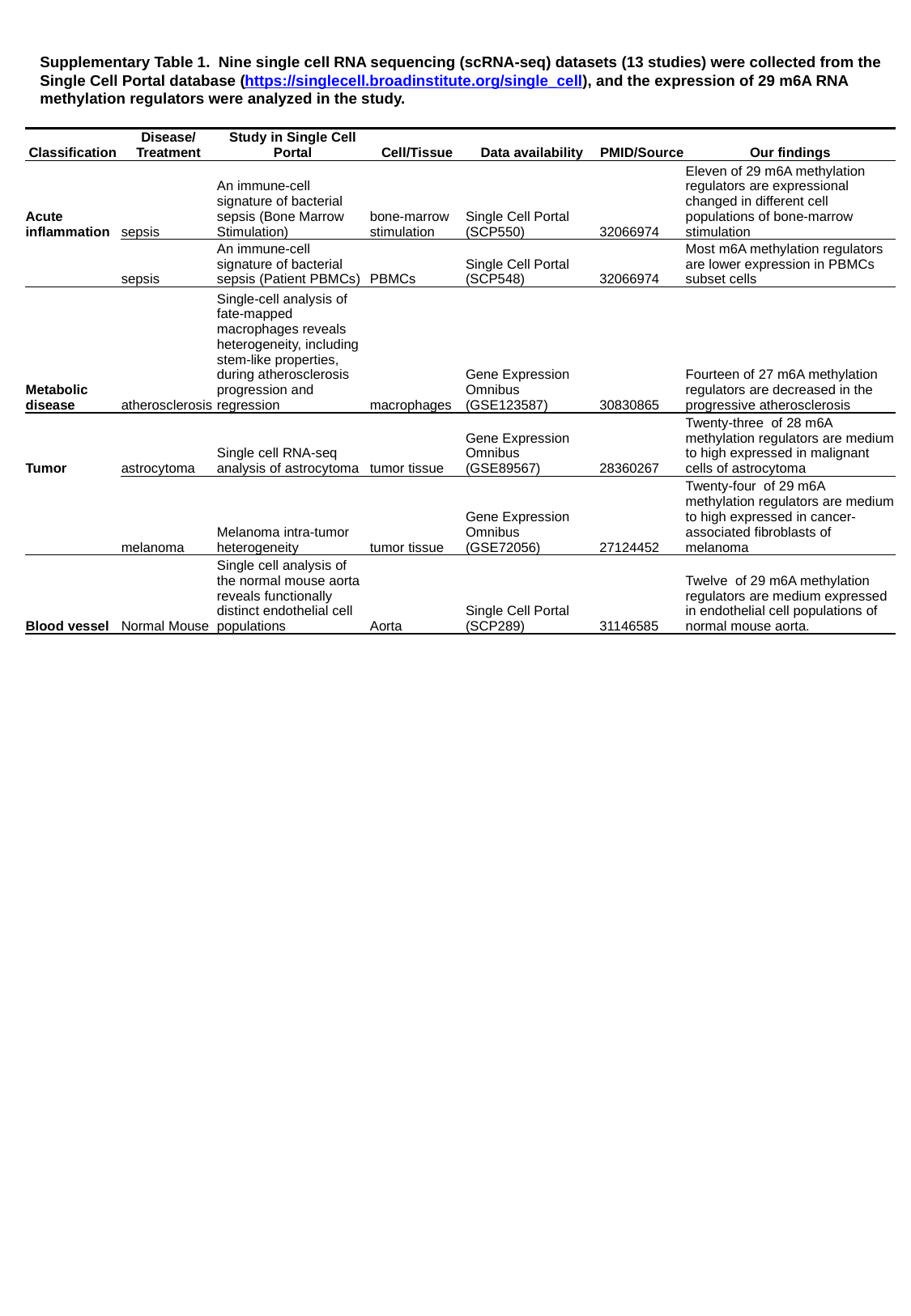

Supplementary Table 1. Nine single cell RNA sequencing (scRNA-seq) datasets (13 studies) were collected from the Single Cell Portal database (https://singlecell.broadinstitute.org/single_cell), and the expression of 29 m6A RNA methylation regulators were analyzed in the study.
| Classification | Disease/ Treatment | Study in Single Cell Portal | Cell/Tissue | Data availability | PMID/Source | Our findings |
| --- | --- | --- | --- | --- | --- | --- |
| Acute inflammation | sepsis | An immune-cell signature of bacterial sepsis (Bone Marrow Stimulation) | bone-marrow stimulation | Single Cell Portal (SCP550) | 32066974 | Eleven of 29 m6A methylation regulators are expressional changed in different cell populations of bone-marrow stimulation |
| | sepsis | An immune-cell signature of bacterial sepsis (Patient PBMCs) | PBMCs | Single Cell Portal (SCP548) | 32066974 | Most m6A methylation regulators are lower expression in PBMCs subset cells |
| Metabolic disease | atherosclerosis | Single-cell analysis of fate-mapped macrophages reveals heterogeneity, including stem-like properties, during atherosclerosis progression and regression | macrophages | Gene Expression Omnibus (GSE123587) | 30830865 | Fourteen of 27 m6A methylation regulators are decreased in the progressive atherosclerosis |
| Tumor | astrocytoma | Single cell RNA-seq analysis of astrocytoma | tumor tissue | Gene Expression Omnibus (GSE89567) | 28360267 | Twenty-three of 28 m6A methylation regulators are medium to high expressed in malignant cells of astrocytoma |
| | melanoma | Melanoma intra-tumor heterogeneity | tumor tissue | Gene Expression Omnibus (GSE72056) | 27124452 | Twenty-four of 29 m6A methylation regulators are medium to high expressed in cancer-associated fibroblasts of melanoma |
| Blood vessel | Normal Mouse | Single cell analysis of the normal mouse aorta reveals functionally distinct endothelial cell populations | Aorta | Single Cell Portal (SCP289) | 31146585 | Twelve of 29 m6A methylation regulators are medium expressed in endothelial cell populations of normal mouse aorta. |

## Slide 2
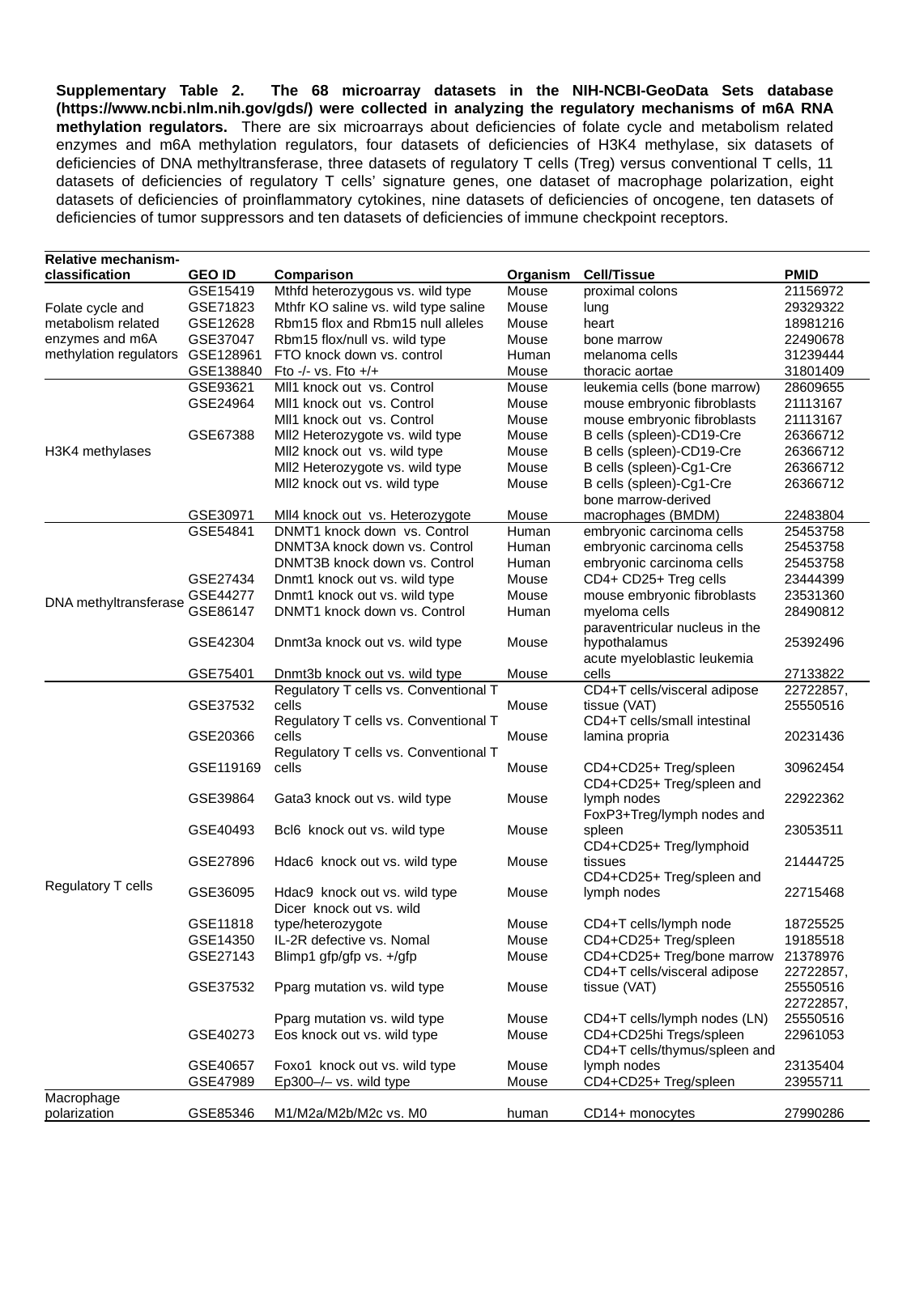

Supplementary Table 2. The 68 microarray datasets in the NIH-NCBI-GeoData Sets database (https://www.ncbi.nlm.nih.gov/gds/) were collected in analyzing the regulatory mechanisms of m6A RNA methylation regulators. There are six microarrays about deficiencies of folate cycle and metabolism related enzymes and m6A methylation regulators, four datasets of deficiencies of H3K4 methylase, six datasets of deficiencies of DNA methyltransferase, three datasets of regulatory T cells (Treg) versus conventional T cells, 11 datasets of deficiencies of regulatory T cells’ signature genes, one dataset of macrophage polarization, eight datasets of deficiencies of proinflammatory cytokines, nine datasets of deficiencies of oncogene, ten datasets of deficiencies of tumor suppressors and ten datasets of deficiencies of immune checkpoint receptors.
| Relative mechanism-classification | GEO ID | Comparison | Organism | Cell/Tissue | PMID |
| --- | --- | --- | --- | --- | --- |
| Folate cycle and metabolism related enzymes and m6A methylation regulators | GSE15419 | Mthfd heterozygous vs. wild type | Mouse | proximal colons | 21156972 |
| | GSE71823 | Mthfr KO saline vs. wild type saline | Mouse | lung | 29329322 |
| | GSE12628 | Rbm15 flox and Rbm15 null alleles | Mouse | heart | 18981216 |
| | GSE37047 | Rbm15 flox/null vs. wild type | Mouse | bone marrow | 22490678 |
| | GSE128961 | FTO knock down vs. control | Human | melanoma cells | 31239444 |
| | GSE138840 | Fto -/- vs. Fto +/+ | Mouse | thoracic aortae | 31801409 |
| H3K4 methylases | GSE93621 | Mll1 knock out vs. Control | Mouse | leukemia cells (bone marrow) | 28609655 |
| | GSE24964 | Mll1 knock out vs. Control | Mouse | mouse embryonic fibroblasts | 21113167 |
| | | Mll1 knock out vs. Control | Mouse | mouse embryonic fibroblasts | 21113167 |
| | GSE67388 | Mll2 Heterozygote vs. wild type | Mouse | B cells (spleen)-CD19-Cre | 26366712 |
| | | Mll2 knock out vs. wild type | Mouse | B cells (spleen)-CD19-Cre | 26366712 |
| | | Mll2 Heterozygote vs. wild type | Mouse | B cells (spleen)-Cg1-Cre | 26366712 |
| | | Mll2 knock out vs. wild type | Mouse | B cells (spleen)-Cg1-Cre | 26366712 |
| | GSE30971 | Mll4 knock out vs. Heterozygote | Mouse | bone marrow-derived macrophages (BMDM) | 22483804 |
| DNA methyltransferase | GSE54841 | DNMT1 knock down vs. Control | Human | embryonic carcinoma cells | 25453758 |
| | | DNMT3A knock down vs. Control | Human | embryonic carcinoma cells | 25453758 |
| | | DNMT3B knock down vs. Control | Human | embryonic carcinoma cells | 25453758 |
| | GSE27434 | Dnmt1 knock out vs. wild type | Mouse | CD4+ CD25+ Treg cells | 23444399 |
| | GSE44277 | Dnmt1 knock out vs. wild type | Mouse | mouse embryonic fibroblasts | 23531360 |
| | GSE86147 | DNMT1 knock down vs. Control | Human | myeloma cells | 28490812 |
| | GSE42304 | Dnmt3a knock out vs. wild type | Mouse | paraventricular nucleus in the hypothalamus | 25392496 |
| | GSE75401 | Dnmt3b knock out vs. wild type | Mouse | acute myeloblastic leukemia cells | 27133822 |
| Regulatory T cells | GSE37532 | Regulatory T cells vs. Conventional T cells | Mouse | CD4+T cells/visceral adipose tissue (VAT) | 22722857, 25550516 |
| | GSE20366 | Regulatory T cells vs. Conventional T cells | Mouse | CD4+T cells/small intestinal lamina propria | 20231436 |
| | GSE119169 | Regulatory T cells vs. Conventional T cells | Mouse | CD4+CD25+ Treg/spleen | 30962454 |
| | GSE39864 | Gata3 knock out vs. wild type | Mouse | CD4+CD25+ Treg/spleen and lymph nodes | 22922362 |
| | GSE40493 | Bcl6 knock out vs. wild type | Mouse | FoxP3+Treg/lymph nodes and spleen | 23053511 |
| | GSE27896 | Hdac6 knock out vs. wild type | Mouse | CD4+CD25+ Treg/lymphoid tissues | 21444725 |
| | GSE36095 | Hdac9 knock out vs. wild type | Mouse | CD4+CD25+ Treg/spleen and lymph nodes | 22715468 |
| | GSE11818 | Dicer knock out vs. wild type/heterozygote | Mouse | CD4+T cells/lymph node | 18725525 |
| | GSE14350 | IL-2R defective vs. Nomal | Mouse | CD4+CD25+ Treg/spleen | 19185518 |
| | GSE27143 | Blimp1 gfp/gfp vs. +/gfp | Mouse | CD4+CD25+ Treg/bone marrow | 21378976 |
| | GSE37532 | Pparg mutation vs. wild type | Mouse | CD4+T cells/visceral adipose tissue (VAT) | 22722857, 25550516 |
| | | Pparg mutation vs. wild type | Mouse | CD4+T cells/lymph nodes (LN) | 22722857, 25550516 |
| | GSE40273 | Eos knock out vs. wild type | Mouse | CD4+CD25hi Tregs/spleen | 22961053 |
| | GSE40657 | Foxo1 knock out vs. wild type | Mouse | CD4+T cells/thymus/spleen and lymph nodes | 23135404 |
| | GSE47989 | Ep300–/– vs. wild type | Mouse | CD4+CD25+ Treg/spleen | 23955711 |
| Macrophage polarization | GSE85346 | M1/M2a/M2b/M2c vs. M0 | human | CD14+ monocytes | 27990286 |

## Slide 3
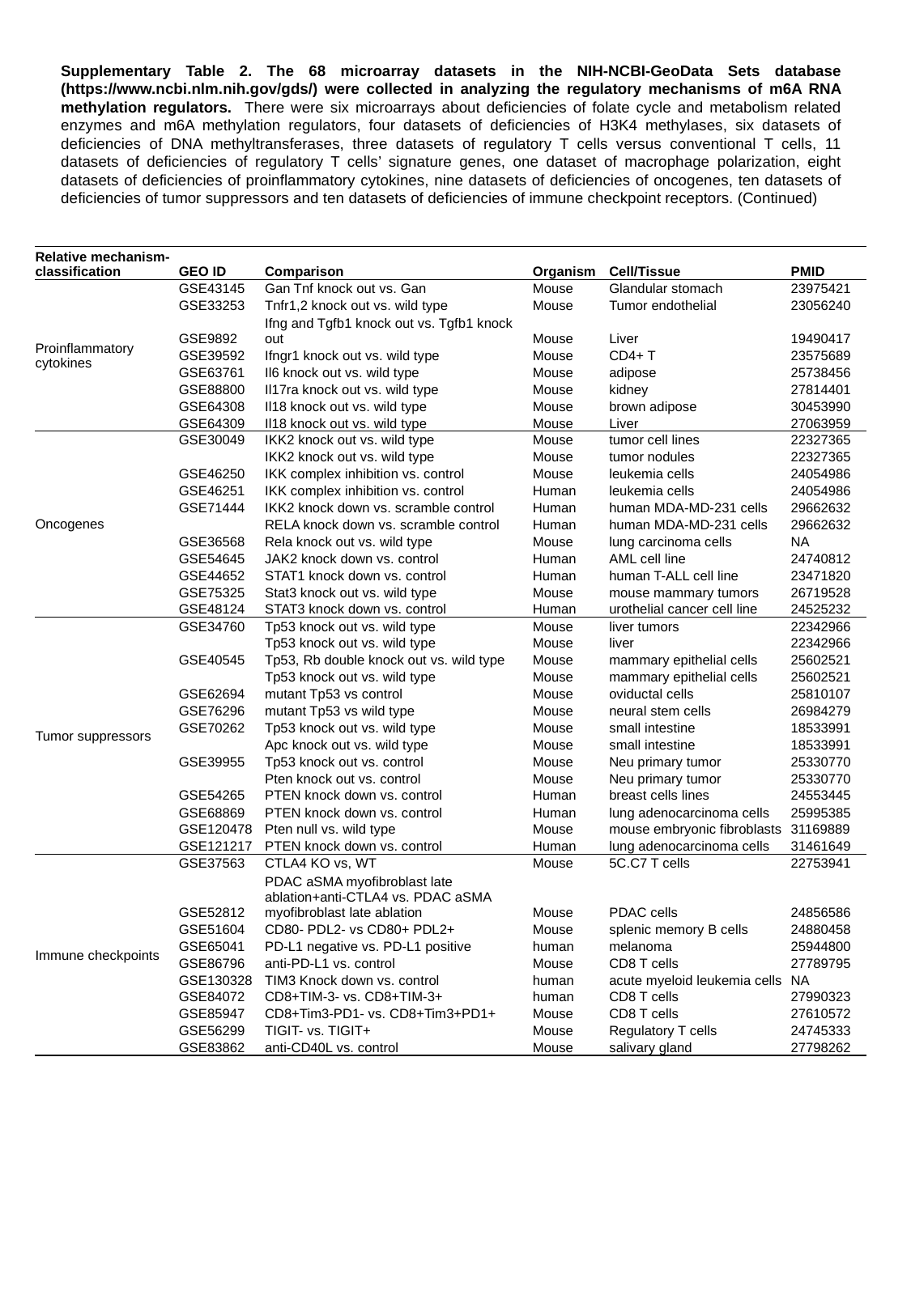

Supplementary Table 2. The 68 microarray datasets in the NIH-NCBI-GeoData Sets database (https://www.ncbi.nlm.nih.gov/gds/) were collected in analyzing the regulatory mechanisms of m6A RNA methylation regulators. There were six microarrays about deficiencies of folate cycle and metabolism related enzymes and m6A methylation regulators, four datasets of deficiencies of H3K4 methylases, six datasets of deficiencies of DNA methyltransferases, three datasets of regulatory T cells versus conventional T cells, 11 datasets of deficiencies of regulatory T cells’ signature genes, one dataset of macrophage polarization, eight datasets of deficiencies of proinflammatory cytokines, nine datasets of deficiencies of oncogenes, ten datasets of deficiencies of tumor suppressors and ten datasets of deficiencies of immune checkpoint receptors. (Continued)
| Relative mechanism-classification | GEO ID | Comparison | Organism | Cell/Tissue | PMID |
| --- | --- | --- | --- | --- | --- |
| Proinflammatory cytokines | GSE43145 | Gan Tnf knock out vs. Gan | Mouse | Glandular stomach | 23975421 |
| | GSE33253 | Tnfr1,2 knock out vs. wild type | Mouse | Tumor endothelial | 23056240 |
| | GSE9892 | Ifng and Tgfb1 knock out vs. Tgfb1 knock out | Mouse | Liver | 19490417 |
| | GSE39592 | Ifngr1 knock out vs. wild type | Mouse | CD4+ T | 23575689 |
| | GSE63761 | Il6 knock out vs. wild type | Mouse | adipose | 25738456 |
| | GSE88800 | Il17ra knock out vs. wild type | Mouse | kidney | 27814401 |
| | GSE64308 | Il18 knock out vs. wild type | Mouse | brown adipose | 30453990 |
| | GSE64309 | Il18 knock out vs. wild type | Mouse | Liver | 27063959 |
| Oncogenes | GSE30049 | IKK2 knock out vs. wild type | Mouse | tumor cell lines | 22327365 |
| | | IKK2 knock out vs. wild type | Mouse | tumor nodules | 22327365 |
| | GSE46250 | IKK complex inhibition vs. control | Mouse | leukemia cells | 24054986 |
| | GSE46251 | IKK complex inhibition vs. control | Human | leukemia cells | 24054986 |
| | GSE71444 | IKK2 knock down vs. scramble control | Human | human MDA-MD-231 cells | 29662632 |
| | | RELA knock down vs. scramble control | Human | human MDA-MD-231 cells | 29662632 |
| | GSE36568 | Rela knock out vs. wild type | Mouse | lung carcinoma cells | NA |
| | GSE54645 | JAK2 knock down vs. control | Human | AML cell line | 24740812 |
| | GSE44652 | STAT1 knock down vs. control | Human | human T-ALL cell line | 23471820 |
| | GSE75325 | Stat3 knock out vs. wild type | Mouse | mouse mammary tumors | 26719528 |
| | GSE48124 | STAT3 knock down vs. control | Human | urothelial cancer cell line | 24525232 |
| Tumor suppressors | GSE34760 | Tp53 knock out vs. wild type | Mouse | liver tumors | 22342966 |
| | | Tp53 knock out vs. wild type | Mouse | liver | 22342966 |
| | GSE40545 | Tp53, Rb double knock out vs. wild type | Mouse | mammary epithelial cells | 25602521 |
| | | Tp53 knock out vs. wild type | Mouse | mammary epithelial cells | 25602521 |
| | GSE62694 | mutant Tp53 vs control | Mouse | oviductal cells | 25810107 |
| | GSE76296 | mutant Tp53 vs wild type | Mouse | neural stem cells | 26984279 |
| | GSE70262 | Tp53 knock out vs. wild type | Mouse | small intestine | 18533991 |
| | | Apc knock out vs. wild type | Mouse | small intestine | 18533991 |
| | GSE39955 | Tp53 knock out vs. control | Mouse | Neu primary tumor | 25330770 |
| | | Pten knock out vs. control | Mouse | Neu primary tumor | 25330770 |
| | GSE54265 | PTEN knock down vs. control | Human | breast cells lines | 24553445 |
| | GSE68869 | PTEN knock down vs. control | Human | lung adenocarcinoma cells | 25995385 |
| | GSE120478 | Pten null vs. wild type | Mouse | mouse embryonic fibroblasts | 31169889 |
| | GSE121217 | PTEN knock down vs. control | Human | lung adenocarcinoma cells | 31461649 |
| Immune checkpoints | GSE37563 | CTLA4 KO vs, WT | Mouse | 5C.C7 T cells | 22753941 |
| | GSE52812 | PDAC aSMA myofibroblast late ablation+anti-CTLA4 vs. PDAC aSMA myofibroblast late ablation | Mouse | PDAC cells | 24856586 |
| | GSE51604 | CD80- PDL2- vs CD80+ PDL2+ | Mouse | splenic memory B cells | 24880458 |
| | GSE65041 | PD-L1 negative vs. PD-L1 positive | human | melanoma | 25944800 |
| | GSE86796 | anti-PD-L1 vs. control | Mouse | CD8 T cells | 27789795 |
| | GSE130328 | TIM3 Knock down vs. control | human | acute myeloid leukemia cells | NA |
| | GSE84072 | CD8+TIM-3- vs. CD8+TIM-3+ | human | CD8 T cells | 27990323 |
| | GSE85947 | CD8+Tim3-PD1- vs. CD8+Tim3+PD1+ | Mouse | CD8 T cells | 27610572 |
| | GSE56299 | TIGIT- vs. TIGIT+ | Mouse | Regulatory T cells | 24745333 |
| | GSE83862 | anti-CD40L vs. control | Mouse | salivary gland | 27798262 |

## Slide 4
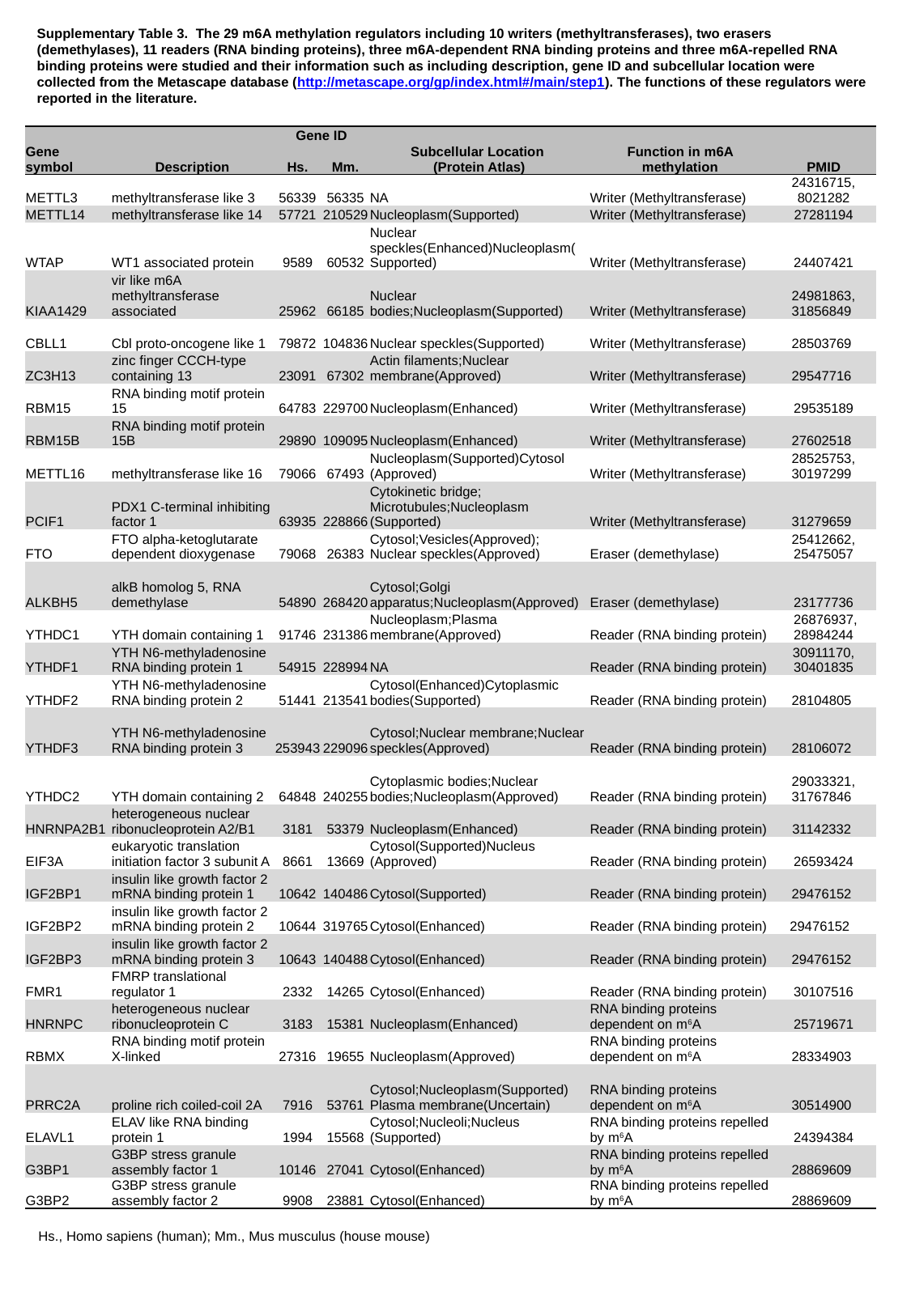

Supplementary Table 3. The 29 m6A methylation regulators including 10 writers (methyltransferases), two erasers (demethylases), 11 readers (RNA binding proteins), three m6A-dependent RNA binding proteins and three m6A-repelled RNA binding proteins were studied and their information such as including description, gene ID and subcellular location were collected from the Metascape database (http://metascape.org/gp/index.html#/main/step1). The functions of these regulators were reported in the literature.
| Gene symbol | Description | Gene ID | | Subcellular Location (Protein Atlas) | Function in m6A methylation | PMID |
| --- | --- | --- | --- | --- | --- | --- |
| | | Hs. | Mm. | | | |
| METTL3 | methyltransferase like 3 | 56339 | 56335 | NA | Writer (Methyltransferase) | 24316715, 8021282 |
| METTL14 | methyltransferase like 14 | 57721 | 210529 | Nucleoplasm(Supported) | Writer (Methyltransferase) | 27281194 |
| WTAP | WT1 associated protein | 9589 | 60532 | Nuclear speckles(Enhanced)Nucleoplasm( Supported) | Writer (Methyltransferase) | 24407421 |
| KIAA1429 | vir like m6A methyltransferase associated | 25962 | 66185 | Nuclear bodies;Nucleoplasm(Supported) | Writer (Methyltransferase) | 24981863, 31856849 |
| CBLL1 | Cbl proto-oncogene like 1 | 79872 | 104836 | Nuclear speckles(Supported) | Writer (Methyltransferase) | 28503769 |
| ZC3H13 | zinc finger CCCH-type containing 13 | 23091 | 67302 | Actin filaments;Nuclear membrane(Approved) | Writer (Methyltransferase) | 29547716 |
| RBM15 | RNA binding motif protein 15 | 64783 | 229700 | Nucleoplasm(Enhanced) | Writer (Methyltransferase) | 29535189 |
| RBM15B | RNA binding motif protein 15B | 29890 | 109095 | Nucleoplasm(Enhanced) | Writer (Methyltransferase) | 27602518 |
| METTL16 | methyltransferase like 16 | 79066 | 67493 | Nucleoplasm(Supported)Cytosol (Approved) | Writer (Methyltransferase) | 28525753, 30197299 |
| PCIF1 | PDX1 C-terminal inhibiting factor 1 | 63935 | 228866 | Cytokinetic bridge; Microtubules;Nucleoplasm (Supported) | Writer (Methyltransferase) | 31279659 |
| FTO | FTO alpha-ketoglutarate dependent dioxygenase | 79068 | 26383 | Cytosol;Vesicles(Approved); Nuclear speckles(Approved) | Eraser (demethylase) | 25412662, 25475057 |
| ALKBH5 | alkB homolog 5, RNA demethylase | 54890 | 268420 | Cytosol;Golgi apparatus;Nucleoplasm(Approved) | Eraser (demethylase) | 23177736 |
| YTHDC1 | YTH domain containing 1 | 91746 | 231386 | Nucleoplasm;Plasma membrane(Approved) | Reader (RNA binding protein) | 26876937, 28984244 |
| YTHDF1 | YTH N6-methyladenosine RNA binding protein 1 | 54915 | 228994 | NA | Reader (RNA binding protein) | 30911170, 30401835 |
| YTHDF2 | YTH N6-methyladenosine RNA binding protein 2 | 51441 | 213541 | Cytosol(Enhanced)Cytoplasmic bodies(Supported) | Reader (RNA binding protein) | 28104805 |
| YTHDF3 | YTH N6-methyladenosine RNA binding protein 3 | 253943 | 229096 | Cytosol;Nuclear membrane;Nuclear speckles(Approved) | Reader (RNA binding protein) | 28106072 |
| YTHDC2 | YTH domain containing 2 | 64848 | 240255 | Cytoplasmic bodies;Nuclear bodies;Nucleoplasm(Approved) | Reader (RNA binding protein) | 29033321, 31767846 |
| HNRNPA2B1 | heterogeneous nuclear ribonucleoprotein A2/B1 | 3181 | 53379 | Nucleoplasm(Enhanced) | Reader (RNA binding protein) | 31142332 |
| EIF3A | eukaryotic translation initiation factor 3 subunit A | 8661 | 13669 | Cytosol(Supported)Nucleus (Approved) | Reader (RNA binding protein) | 26593424 |
| IGF2BP1 | insulin like growth factor 2 mRNA binding protein 1 | 10642 | 140486 | Cytosol(Supported) | Reader (RNA binding protein) | 29476152 |
| IGF2BP2 | insulin like growth factor 2 mRNA binding protein 2 | 10644 | 319765 | Cytosol(Enhanced) | Reader (RNA binding protein) | 29476152 |
| IGF2BP3 | insulin like growth factor 2 mRNA binding protein 3 | 10643 | 140488 | Cytosol(Enhanced) | Reader (RNA binding protein) | 29476152 |
| FMR1 | FMRP translational regulator 1 | 2332 | 14265 | Cytosol(Enhanced) | Reader (RNA binding protein) | 30107516 |
| HNRNPC | heterogeneous nuclear ribonucleoprotein C | 3183 | 15381 | Nucleoplasm(Enhanced) | RNA binding proteins dependent on m6A | 25719671 |
| RBMX | RNA binding motif protein X-linked | 27316 | 19655 | Nucleoplasm(Approved) | RNA binding proteins dependent on m6A | 28334903 |
| PRRC2A | proline rich coiled-coil 2A | 7916 | 53761 | Cytosol;Nucleoplasm(Supported) Plasma membrane(Uncertain) | RNA binding proteins dependent on m6A | 30514900 |
| ELAVL1 | ELAV like RNA binding protein 1 | 1994 | 15568 | Cytosol;Nucleoli;Nucleus (Supported) | RNA binding proteins repelled by m6A | 24394384 |
| G3BP1 | G3BP stress granule assembly factor 1 | 10146 | 27041 | Cytosol(Enhanced) | RNA binding proteins repelled by m6A | 28869609 |
| G3BP2 | G3BP stress granule assembly factor 2 | 9908 | 23881 | Cytosol(Enhanced) | RNA binding proteins repelled by m6A | 28869609 |
Hs., Homo sapiens (human); Mm., Mus musculus (house mouse)

## Slide 5
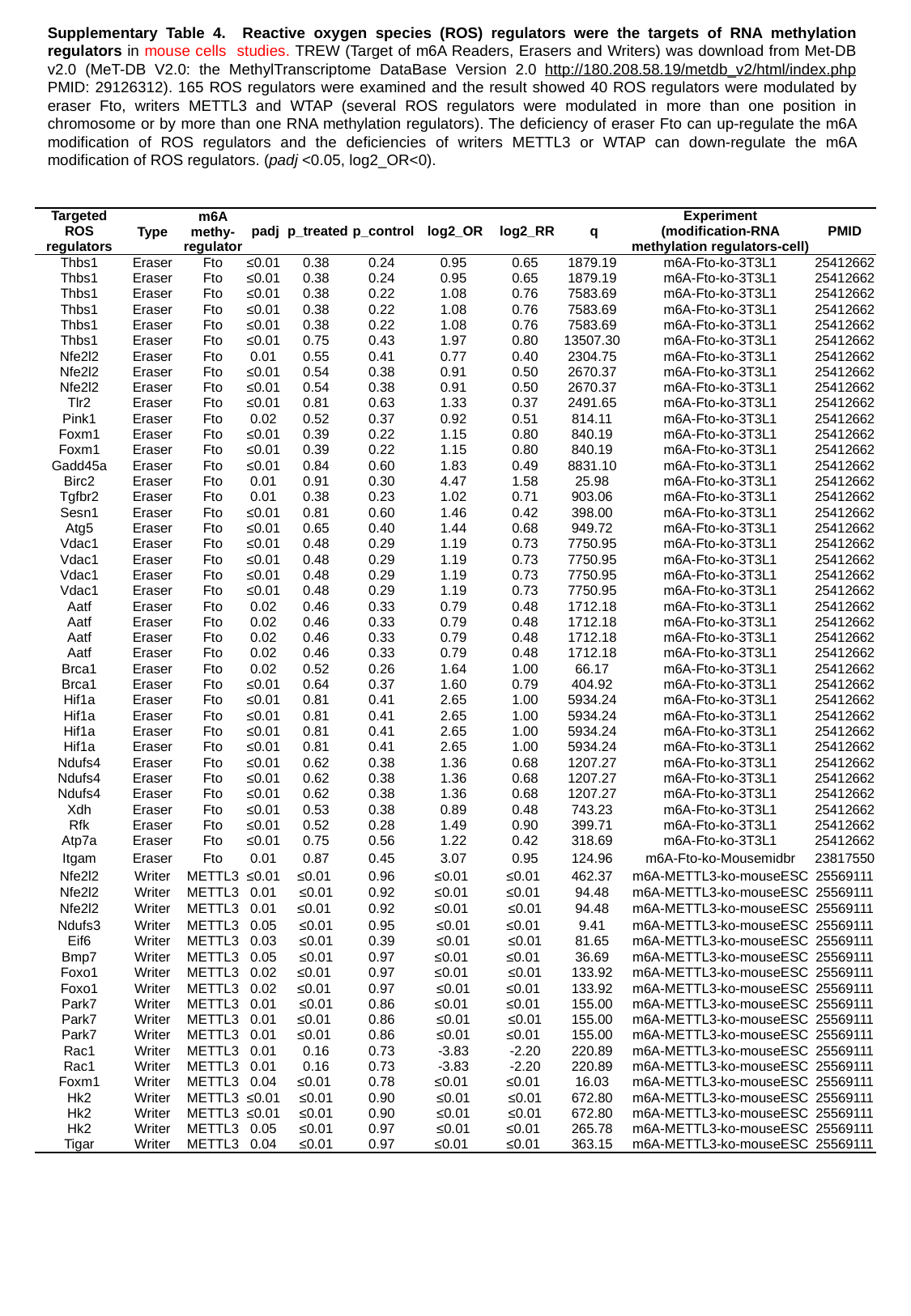

Supplementary Table 4. Reactive oxygen species (ROS) regulators were the targets of RNA methylation regulators in mouse cells studies. TREW (Target of m6A Readers, Erasers and Writers) was download from Met-DB v2.0 (MeT-DB V2.0: the MethylTranscriptome DataBase Version 2.0 http://180.208.58.19/metdb_v2/html/index.php PMID: 29126312). 165 ROS regulators were examined and the result showed 40 ROS regulators were modulated by eraser Fto, writers METTL3 and WTAP (several ROS regulators were modulated in more than one position in chromosome or by more than one RNA methylation regulators). The deficiency of eraser Fto can up-regulate the m6A modification of ROS regulators and the deficiencies of writers METTL3 or WTAP can down-regulate the m6A modification of ROS regulators. (padj <0.05, log2_OR<0).
| Targeted ROS regulators | Type | m6A methy-regulator | padj | p\_treated | p\_control | log2\_OR | log2\_RR | q | Experiment (modification-RNA methylation regulators-cell) | PMID |
| --- | --- | --- | --- | --- | --- | --- | --- | --- | --- | --- |
| Thbs1 | Eraser | Fto | ≤0.01 | 0.38 | 0.24 | 0.95 | 0.65 | 1879.19 | m6A-Fto-ko-3T3L1 | 25412662 |
| Thbs1 | Eraser | Fto | ≤0.01 | 0.38 | 0.24 | 0.95 | 0.65 | 1879.19 | m6A-Fto-ko-3T3L1 | 25412662 |
| Thbs1 | Eraser | Fto | ≤0.01 | 0.38 | 0.22 | 1.08 | 0.76 | 7583.69 | m6A-Fto-ko-3T3L1 | 25412662 |
| Thbs1 | Eraser | Fto | ≤0.01 | 0.38 | 0.22 | 1.08 | 0.76 | 7583.69 | m6A-Fto-ko-3T3L1 | 25412662 |
| Thbs1 | Eraser | Fto | ≤0.01 | 0.38 | 0.22 | 1.08 | 0.76 | 7583.69 | m6A-Fto-ko-3T3L1 | 25412662 |
| Thbs1 | Eraser | Fto | ≤0.01 | 0.75 | 0.43 | 1.97 | 0.80 | 13507.30 | m6A-Fto-ko-3T3L1 | 25412662 |
| Nfe2l2 | Eraser | Fto | 0.01 | 0.55 | 0.41 | 0.77 | 0.40 | 2304.75 | m6A-Fto-ko-3T3L1 | 25412662 |
| Nfe2l2 | Eraser | Fto | ≤0.01 | 0.54 | 0.38 | 0.91 | 0.50 | 2670.37 | m6A-Fto-ko-3T3L1 | 25412662 |
| Nfe2l2 | Eraser | Fto | ≤0.01 | 0.54 | 0.38 | 0.91 | 0.50 | 2670.37 | m6A-Fto-ko-3T3L1 | 25412662 |
| Tlr2 | Eraser | Fto | ≤0.01 | 0.81 | 0.63 | 1.33 | 0.37 | 2491.65 | m6A-Fto-ko-3T3L1 | 25412662 |
| Pink1 | Eraser | Fto | 0.02 | 0.52 | 0.37 | 0.92 | 0.51 | 814.11 | m6A-Fto-ko-3T3L1 | 25412662 |
| Foxm1 | Eraser | Fto | ≤0.01 | 0.39 | 0.22 | 1.15 | 0.80 | 840.19 | m6A-Fto-ko-3T3L1 | 25412662 |
| Foxm1 | Eraser | Fto | ≤0.01 | 0.39 | 0.22 | 1.15 | 0.80 | 840.19 | m6A-Fto-ko-3T3L1 | 25412662 |
| Gadd45a | Eraser | Fto | ≤0.01 | 0.84 | 0.60 | 1.83 | 0.49 | 8831.10 | m6A-Fto-ko-3T3L1 | 25412662 |
| Birc2 | Eraser | Fto | 0.01 | 0.91 | 0.30 | 4.47 | 1.58 | 25.98 | m6A-Fto-ko-3T3L1 | 25412662 |
| Tgfbr2 | Eraser | Fto | 0.01 | 0.38 | 0.23 | 1.02 | 0.71 | 903.06 | m6A-Fto-ko-3T3L1 | 25412662 |
| Sesn1 | Eraser | Fto | ≤0.01 | 0.81 | 0.60 | 1.46 | 0.42 | 398.00 | m6A-Fto-ko-3T3L1 | 25412662 |
| Atg5 | Eraser | Fto | ≤0.01 | 0.65 | 0.40 | 1.44 | 0.68 | 949.72 | m6A-Fto-ko-3T3L1 | 25412662 |
| Vdac1 | Eraser | Fto | ≤0.01 | 0.48 | 0.29 | 1.19 | 0.73 | 7750.95 | m6A-Fto-ko-3T3L1 | 25412662 |
| Vdac1 | Eraser | Fto | ≤0.01 | 0.48 | 0.29 | 1.19 | 0.73 | 7750.95 | m6A-Fto-ko-3T3L1 | 25412662 |
| Vdac1 | Eraser | Fto | ≤0.01 | 0.48 | 0.29 | 1.19 | 0.73 | 7750.95 | m6A-Fto-ko-3T3L1 | 25412662 |
| Vdac1 | Eraser | Fto | ≤0.01 | 0.48 | 0.29 | 1.19 | 0.73 | 7750.95 | m6A-Fto-ko-3T3L1 | 25412662 |
| Aatf | Eraser | Fto | 0.02 | 0.46 | 0.33 | 0.79 | 0.48 | 1712.18 | m6A-Fto-ko-3T3L1 | 25412662 |
| Aatf | Eraser | Fto | 0.02 | 0.46 | 0.33 | 0.79 | 0.48 | 1712.18 | m6A-Fto-ko-3T3L1 | 25412662 |
| Aatf | Eraser | Fto | 0.02 | 0.46 | 0.33 | 0.79 | 0.48 | 1712.18 | m6A-Fto-ko-3T3L1 | 25412662 |
| Aatf | Eraser | Fto | 0.02 | 0.46 | 0.33 | 0.79 | 0.48 | 1712.18 | m6A-Fto-ko-3T3L1 | 25412662 |
| Brca1 | Eraser | Fto | 0.02 | 0.52 | 0.26 | 1.64 | 1.00 | 66.17 | m6A-Fto-ko-3T3L1 | 25412662 |
| Brca1 | Eraser | Fto | ≤0.01 | 0.64 | 0.37 | 1.60 | 0.79 | 404.92 | m6A-Fto-ko-3T3L1 | 25412662 |
| Hif1a | Eraser | Fto | ≤0.01 | 0.81 | 0.41 | 2.65 | 1.00 | 5934.24 | m6A-Fto-ko-3T3L1 | 25412662 |
| Hif1a | Eraser | Fto | ≤0.01 | 0.81 | 0.41 | 2.65 | 1.00 | 5934.24 | m6A-Fto-ko-3T3L1 | 25412662 |
| Hif1a | Eraser | Fto | ≤0.01 | 0.81 | 0.41 | 2.65 | 1.00 | 5934.24 | m6A-Fto-ko-3T3L1 | 25412662 |
| Hif1a | Eraser | Fto | ≤0.01 | 0.81 | 0.41 | 2.65 | 1.00 | 5934.24 | m6A-Fto-ko-3T3L1 | 25412662 |
| Ndufs4 | Eraser | Fto | ≤0.01 | 0.62 | 0.38 | 1.36 | 0.68 | 1207.27 | m6A-Fto-ko-3T3L1 | 25412662 |
| Ndufs4 | Eraser | Fto | ≤0.01 | 0.62 | 0.38 | 1.36 | 0.68 | 1207.27 | m6A-Fto-ko-3T3L1 | 25412662 |
| Ndufs4 | Eraser | Fto | ≤0.01 | 0.62 | 0.38 | 1.36 | 0.68 | 1207.27 | m6A-Fto-ko-3T3L1 | 25412662 |
| Xdh | Eraser | Fto | ≤0.01 | 0.53 | 0.38 | 0.89 | 0.48 | 743.23 | m6A-Fto-ko-3T3L1 | 25412662 |
| Rfk | Eraser | Fto | ≤0.01 | 0.52 | 0.28 | 1.49 | 0.90 | 399.71 | m6A-Fto-ko-3T3L1 | 25412662 |
| Atp7a | Eraser | Fto | ≤0.01 | 0.75 | 0.56 | 1.22 | 0.42 | 318.69 | m6A-Fto-ko-3T3L1 | 25412662 |
| Itgam | Eraser | Fto | 0.01 | 0.87 | 0.45 | 3.07 | 0.95 | 124.96 | m6A-Fto-ko-Mousemidbr | 23817550 |
| Nfe2l2 | Writer | METTL3 | ≤0.01 | ≤0.01 | 0.96 | ≤0.01 | ≤0.01 | 462.37 | m6A-METTL3-ko-mouseESC | 25569111 |
| Nfe2l2 | Writer | METTL3 | 0.01 | ≤0.01 | 0.92 | ≤0.01 | ≤0.01 | 94.48 | m6A-METTL3-ko-mouseESC | 25569111 |
| Nfe2l2 | Writer | METTL3 | 0.01 | ≤0.01 | 0.92 | ≤0.01 | ≤0.01 | 94.48 | m6A-METTL3-ko-mouseESC | 25569111 |
| Ndufs3 | Writer | METTL3 | 0.05 | ≤0.01 | 0.95 | ≤0.01 | ≤0.01 | 9.41 | m6A-METTL3-ko-mouseESC | 25569111 |
| Eif6 | Writer | METTL3 | 0.03 | ≤0.01 | 0.39 | ≤0.01 | ≤0.01 | 81.65 | m6A-METTL3-ko-mouseESC | 25569111 |
| Bmp7 | Writer | METTL3 | 0.05 | ≤0.01 | 0.97 | ≤0.01 | ≤0.01 | 36.69 | m6A-METTL3-ko-mouseESC | 25569111 |
| Foxo1 | Writer | METTL3 | 0.02 | ≤0.01 | 0.97 | ≤0.01 | ≤0.01 | 133.92 | m6A-METTL3-ko-mouseESC | 25569111 |
| Foxo1 | Writer | METTL3 | 0.02 | ≤0.01 | 0.97 | ≤0.01 | ≤0.01 | 133.92 | m6A-METTL3-ko-mouseESC | 25569111 |
| Park7 | Writer | METTL3 | 0.01 | ≤0.01 | 0.86 | ≤0.01 | ≤0.01 | 155.00 | m6A-METTL3-ko-mouseESC | 25569111 |
| Park7 | Writer | METTL3 | 0.01 | ≤0.01 | 0.86 | ≤0.01 | ≤0.01 | 155.00 | m6A-METTL3-ko-mouseESC | 25569111 |
| Park7 | Writer | METTL3 | 0.01 | ≤0.01 | 0.86 | ≤0.01 | ≤0.01 | 155.00 | m6A-METTL3-ko-mouseESC | 25569111 |
| Rac1 | Writer | METTL3 | 0.01 | 0.16 | 0.73 | -3.83 | -2.20 | 220.89 | m6A-METTL3-ko-mouseESC | 25569111 |
| Rac1 | Writer | METTL3 | 0.01 | 0.16 | 0.73 | -3.83 | -2.20 | 220.89 | m6A-METTL3-ko-mouseESC | 25569111 |
| Foxm1 | Writer | METTL3 | 0.04 | ≤0.01 | 0.78 | ≤0.01 | ≤0.01 | 16.03 | m6A-METTL3-ko-mouseESC | 25569111 |
| Hk2 | Writer | METTL3 | ≤0.01 | ≤0.01 | 0.90 | ≤0.01 | ≤0.01 | 672.80 | m6A-METTL3-ko-mouseESC | 25569111 |
| Hk2 | Writer | METTL3 | ≤0.01 | ≤0.01 | 0.90 | ≤0.01 | ≤0.01 | 672.80 | m6A-METTL3-ko-mouseESC | 25569111 |
| Hk2 | Writer | METTL3 | 0.05 | ≤0.01 | 0.97 | ≤0.01 | ≤0.01 | 265.78 | m6A-METTL3-ko-mouseESC | 25569111 |
| Tigar | Writer | METTL3 | 0.04 | ≤0.01 | 0.97 | ≤0.01 | ≤0.01 | 363.15 | m6A-METTL3-ko-mouseESC | 25569111 |

## Slide 6
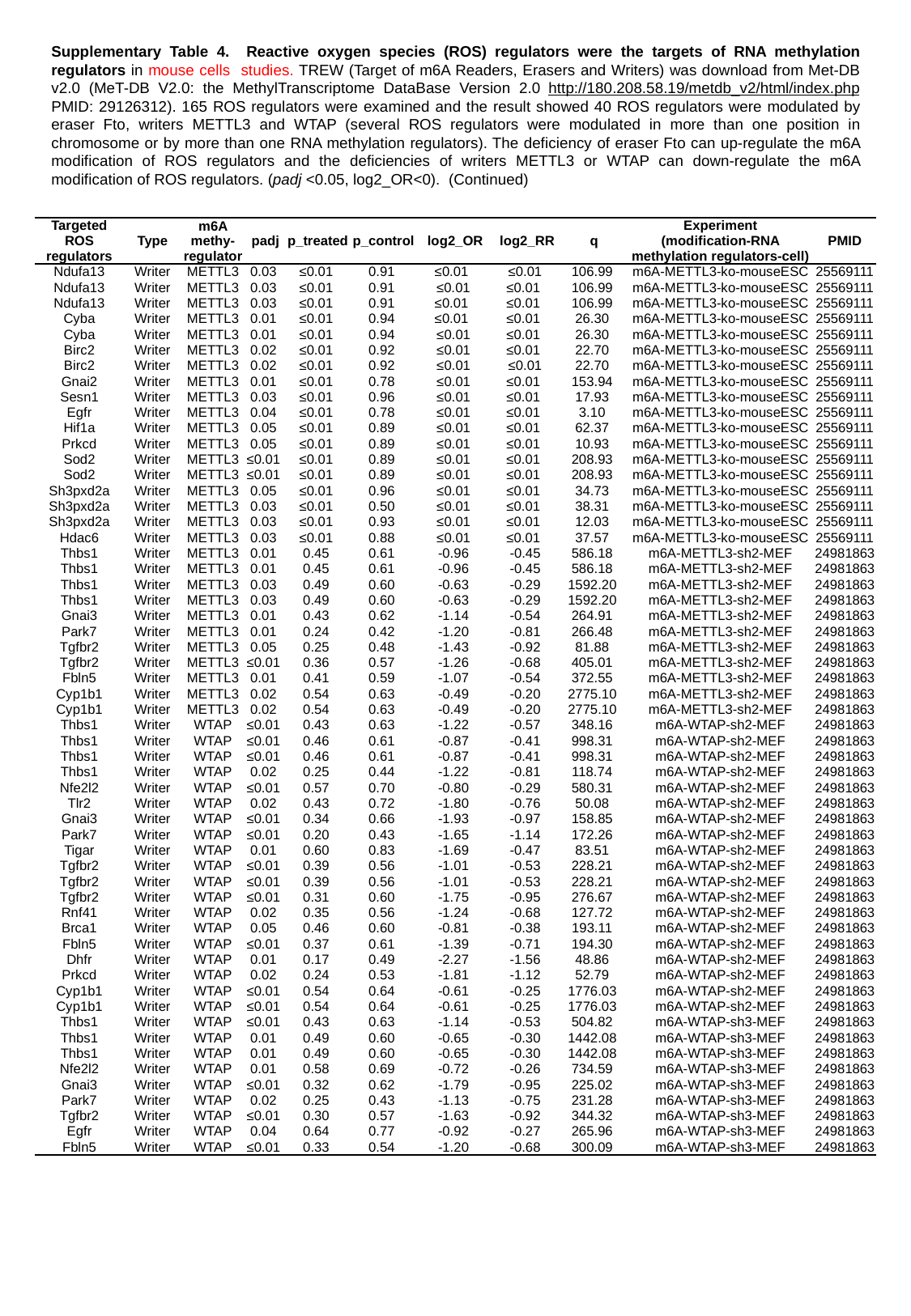

Supplementary Table 4. Reactive oxygen species (ROS) regulators were the targets of RNA methylation regulators in mouse cells studies. TREW (Target of m6A Readers, Erasers and Writers) was download from Met-DB v2.0 (MeT-DB V2.0: the MethylTranscriptome DataBase Version 2.0 http://180.208.58.19/metdb_v2/html/index.php PMID: 29126312). 165 ROS regulators were examined and the result showed 40 ROS regulators were modulated by eraser Fto, writers METTL3 and WTAP (several ROS regulators were modulated in more than one position in chromosome or by more than one RNA methylation regulators). The deficiency of eraser Fto can up-regulate the m6A modification of ROS regulators and the deficiencies of writers METTL3 or WTAP can down-regulate the m6A modification of ROS regulators. (padj <0.05, log2_OR<0). (Continued)
| Targeted ROS regulators | Type | m6A methy-regulator | padj | p\_treated | p\_control | log2\_OR | log2\_RR | q | Experiment (modification-RNA methylation regulators-cell) | PMID |
| --- | --- | --- | --- | --- | --- | --- | --- | --- | --- | --- |
| Ndufa13 | Writer | METTL3 | 0.03 | ≤0.01 | 0.91 | ≤0.01 | ≤0.01 | 106.99 | m6A-METTL3-ko-mouseESC | 25569111 |
| Ndufa13 | Writer | METTL3 | 0.03 | ≤0.01 | 0.91 | ≤0.01 | ≤0.01 | 106.99 | m6A-METTL3-ko-mouseESC | 25569111 |
| Ndufa13 | Writer | METTL3 | 0.03 | ≤0.01 | 0.91 | ≤0.01 | ≤0.01 | 106.99 | m6A-METTL3-ko-mouseESC | 25569111 |
| Cyba | Writer | METTL3 | 0.01 | ≤0.01 | 0.94 | ≤0.01 | ≤0.01 | 26.30 | m6A-METTL3-ko-mouseESC | 25569111 |
| Cyba | Writer | METTL3 | 0.01 | ≤0.01 | 0.94 | ≤0.01 | ≤0.01 | 26.30 | m6A-METTL3-ko-mouseESC | 25569111 |
| Birc2 | Writer | METTL3 | 0.02 | ≤0.01 | 0.92 | ≤0.01 | ≤0.01 | 22.70 | m6A-METTL3-ko-mouseESC | 25569111 |
| Birc2 | Writer | METTL3 | 0.02 | ≤0.01 | 0.92 | ≤0.01 | ≤0.01 | 22.70 | m6A-METTL3-ko-mouseESC | 25569111 |
| Gnai2 | Writer | METTL3 | 0.01 | ≤0.01 | 0.78 | ≤0.01 | ≤0.01 | 153.94 | m6A-METTL3-ko-mouseESC | 25569111 |
| Sesn1 | Writer | METTL3 | 0.03 | ≤0.01 | 0.96 | ≤0.01 | ≤0.01 | 17.93 | m6A-METTL3-ko-mouseESC | 25569111 |
| Egfr | Writer | METTL3 | 0.04 | ≤0.01 | 0.78 | ≤0.01 | ≤0.01 | 3.10 | m6A-METTL3-ko-mouseESC | 25569111 |
| Hif1a | Writer | METTL3 | 0.05 | ≤0.01 | 0.89 | ≤0.01 | ≤0.01 | 62.37 | m6A-METTL3-ko-mouseESC | 25569111 |
| Prkcd | Writer | METTL3 | 0.05 | ≤0.01 | 0.89 | ≤0.01 | ≤0.01 | 10.93 | m6A-METTL3-ko-mouseESC | 25569111 |
| Sod2 | Writer | METTL3 | ≤0.01 | ≤0.01 | 0.89 | ≤0.01 | ≤0.01 | 208.93 | m6A-METTL3-ko-mouseESC | 25569111 |
| Sod2 | Writer | METTL3 | ≤0.01 | ≤0.01 | 0.89 | ≤0.01 | ≤0.01 | 208.93 | m6A-METTL3-ko-mouseESC | 25569111 |
| Sh3pxd2a | Writer | METTL3 | 0.05 | ≤0.01 | 0.96 | ≤0.01 | ≤0.01 | 34.73 | m6A-METTL3-ko-mouseESC | 25569111 |
| Sh3pxd2a | Writer | METTL3 | 0.03 | ≤0.01 | 0.50 | ≤0.01 | ≤0.01 | 38.31 | m6A-METTL3-ko-mouseESC | 25569111 |
| Sh3pxd2a | Writer | METTL3 | 0.03 | ≤0.01 | 0.93 | ≤0.01 | ≤0.01 | 12.03 | m6A-METTL3-ko-mouseESC | 25569111 |
| Hdac6 | Writer | METTL3 | 0.03 | ≤0.01 | 0.88 | ≤0.01 | ≤0.01 | 37.57 | m6A-METTL3-ko-mouseESC | 25569111 |
| Thbs1 | Writer | METTL3 | 0.01 | 0.45 | 0.61 | -0.96 | -0.45 | 586.18 | m6A-METTL3-sh2-MEF | 24981863 |
| Thbs1 | Writer | METTL3 | 0.01 | 0.45 | 0.61 | -0.96 | -0.45 | 586.18 | m6A-METTL3-sh2-MEF | 24981863 |
| Thbs1 | Writer | METTL3 | 0.03 | 0.49 | 0.60 | -0.63 | -0.29 | 1592.20 | m6A-METTL3-sh2-MEF | 24981863 |
| Thbs1 | Writer | METTL3 | 0.03 | 0.49 | 0.60 | -0.63 | -0.29 | 1592.20 | m6A-METTL3-sh2-MEF | 24981863 |
| Gnai3 | Writer | METTL3 | 0.01 | 0.43 | 0.62 | -1.14 | -0.54 | 264.91 | m6A-METTL3-sh2-MEF | 24981863 |
| Park7 | Writer | METTL3 | 0.01 | 0.24 | 0.42 | -1.20 | -0.81 | 266.48 | m6A-METTL3-sh2-MEF | 24981863 |
| Tgfbr2 | Writer | METTL3 | 0.05 | 0.25 | 0.48 | -1.43 | -0.92 | 81.88 | m6A-METTL3-sh2-MEF | 24981863 |
| Tgfbr2 | Writer | METTL3 | ≤0.01 | 0.36 | 0.57 | -1.26 | -0.68 | 405.01 | m6A-METTL3-sh2-MEF | 24981863 |
| Fbln5 | Writer | METTL3 | 0.01 | 0.41 | 0.59 | -1.07 | -0.54 | 372.55 | m6A-METTL3-sh2-MEF | 24981863 |
| Cyp1b1 | Writer | METTL3 | 0.02 | 0.54 | 0.63 | -0.49 | -0.20 | 2775.10 | m6A-METTL3-sh2-MEF | 24981863 |
| Cyp1b1 | Writer | METTL3 | 0.02 | 0.54 | 0.63 | -0.49 | -0.20 | 2775.10 | m6A-METTL3-sh2-MEF | 24981863 |
| Thbs1 | Writer | WTAP | ≤0.01 | 0.43 | 0.63 | -1.22 | -0.57 | 348.16 | m6A-WTAP-sh2-MEF | 24981863 |
| Thbs1 | Writer | WTAP | ≤0.01 | 0.46 | 0.61 | -0.87 | -0.41 | 998.31 | m6A-WTAP-sh2-MEF | 24981863 |
| Thbs1 | Writer | WTAP | ≤0.01 | 0.46 | 0.61 | -0.87 | -0.41 | 998.31 | m6A-WTAP-sh2-MEF | 24981863 |
| Thbs1 | Writer | WTAP | 0.02 | 0.25 | 0.44 | -1.22 | -0.81 | 118.74 | m6A-WTAP-sh2-MEF | 24981863 |
| Nfe2l2 | Writer | WTAP | ≤0.01 | 0.57 | 0.70 | -0.80 | -0.29 | 580.31 | m6A-WTAP-sh2-MEF | 24981863 |
| Tlr2 | Writer | WTAP | 0.02 | 0.43 | 0.72 | -1.80 | -0.76 | 50.08 | m6A-WTAP-sh2-MEF | 24981863 |
| Gnai3 | Writer | WTAP | ≤0.01 | 0.34 | 0.66 | -1.93 | -0.97 | 158.85 | m6A-WTAP-sh2-MEF | 24981863 |
| Park7 | Writer | WTAP | ≤0.01 | 0.20 | 0.43 | -1.65 | -1.14 | 172.26 | m6A-WTAP-sh2-MEF | 24981863 |
| Tigar | Writer | WTAP | 0.01 | 0.60 | 0.83 | -1.69 | -0.47 | 83.51 | m6A-WTAP-sh2-MEF | 24981863 |
| Tgfbr2 | Writer | WTAP | ≤0.01 | 0.39 | 0.56 | -1.01 | -0.53 | 228.21 | m6A-WTAP-sh2-MEF | 24981863 |
| Tgfbr2 | Writer | WTAP | ≤0.01 | 0.39 | 0.56 | -1.01 | -0.53 | 228.21 | m6A-WTAP-sh2-MEF | 24981863 |
| Tgfbr2 | Writer | WTAP | ≤0.01 | 0.31 | 0.60 | -1.75 | -0.95 | 276.67 | m6A-WTAP-sh2-MEF | 24981863 |
| Rnf41 | Writer | WTAP | 0.02 | 0.35 | 0.56 | -1.24 | -0.68 | 127.72 | m6A-WTAP-sh2-MEF | 24981863 |
| Brca1 | Writer | WTAP | 0.05 | 0.46 | 0.60 | -0.81 | -0.38 | 193.11 | m6A-WTAP-sh2-MEF | 24981863 |
| Fbln5 | Writer | WTAP | ≤0.01 | 0.37 | 0.61 | -1.39 | -0.71 | 194.30 | m6A-WTAP-sh2-MEF | 24981863 |
| Dhfr | Writer | WTAP | 0.01 | 0.17 | 0.49 | -2.27 | -1.56 | 48.86 | m6A-WTAP-sh2-MEF | 24981863 |
| Prkcd | Writer | WTAP | 0.02 | 0.24 | 0.53 | -1.81 | -1.12 | 52.79 | m6A-WTAP-sh2-MEF | 24981863 |
| Cyp1b1 | Writer | WTAP | ≤0.01 | 0.54 | 0.64 | -0.61 | -0.25 | 1776.03 | m6A-WTAP-sh2-MEF | 24981863 |
| Cyp1b1 | Writer | WTAP | ≤0.01 | 0.54 | 0.64 | -0.61 | -0.25 | 1776.03 | m6A-WTAP-sh2-MEF | 24981863 |
| Thbs1 | Writer | WTAP | ≤0.01 | 0.43 | 0.63 | -1.14 | -0.53 | 504.82 | m6A-WTAP-sh3-MEF | 24981863 |
| Thbs1 | Writer | WTAP | 0.01 | 0.49 | 0.60 | -0.65 | -0.30 | 1442.08 | m6A-WTAP-sh3-MEF | 24981863 |
| Thbs1 | Writer | WTAP | 0.01 | 0.49 | 0.60 | -0.65 | -0.30 | 1442.08 | m6A-WTAP-sh3-MEF | 24981863 |
| Nfe2l2 | Writer | WTAP | 0.01 | 0.58 | 0.69 | -0.72 | -0.26 | 734.59 | m6A-WTAP-sh3-MEF | 24981863 |
| Gnai3 | Writer | WTAP | ≤0.01 | 0.32 | 0.62 | -1.79 | -0.95 | 225.02 | m6A-WTAP-sh3-MEF | 24981863 |
| Park7 | Writer | WTAP | 0.02 | 0.25 | 0.43 | -1.13 | -0.75 | 231.28 | m6A-WTAP-sh3-MEF | 24981863 |
| Tgfbr2 | Writer | WTAP | ≤0.01 | 0.30 | 0.57 | -1.63 | -0.92 | 344.32 | m6A-WTAP-sh3-MEF | 24981863 |
| Egfr | Writer | WTAP | 0.04 | 0.64 | 0.77 | -0.92 | -0.27 | 265.96 | m6A-WTAP-sh3-MEF | 24981863 |
| Fbln5 | Writer | WTAP | ≤0.01 | 0.33 | 0.54 | -1.20 | -0.68 | 300.09 | m6A-WTAP-sh3-MEF | 24981863 |

## Slide 7
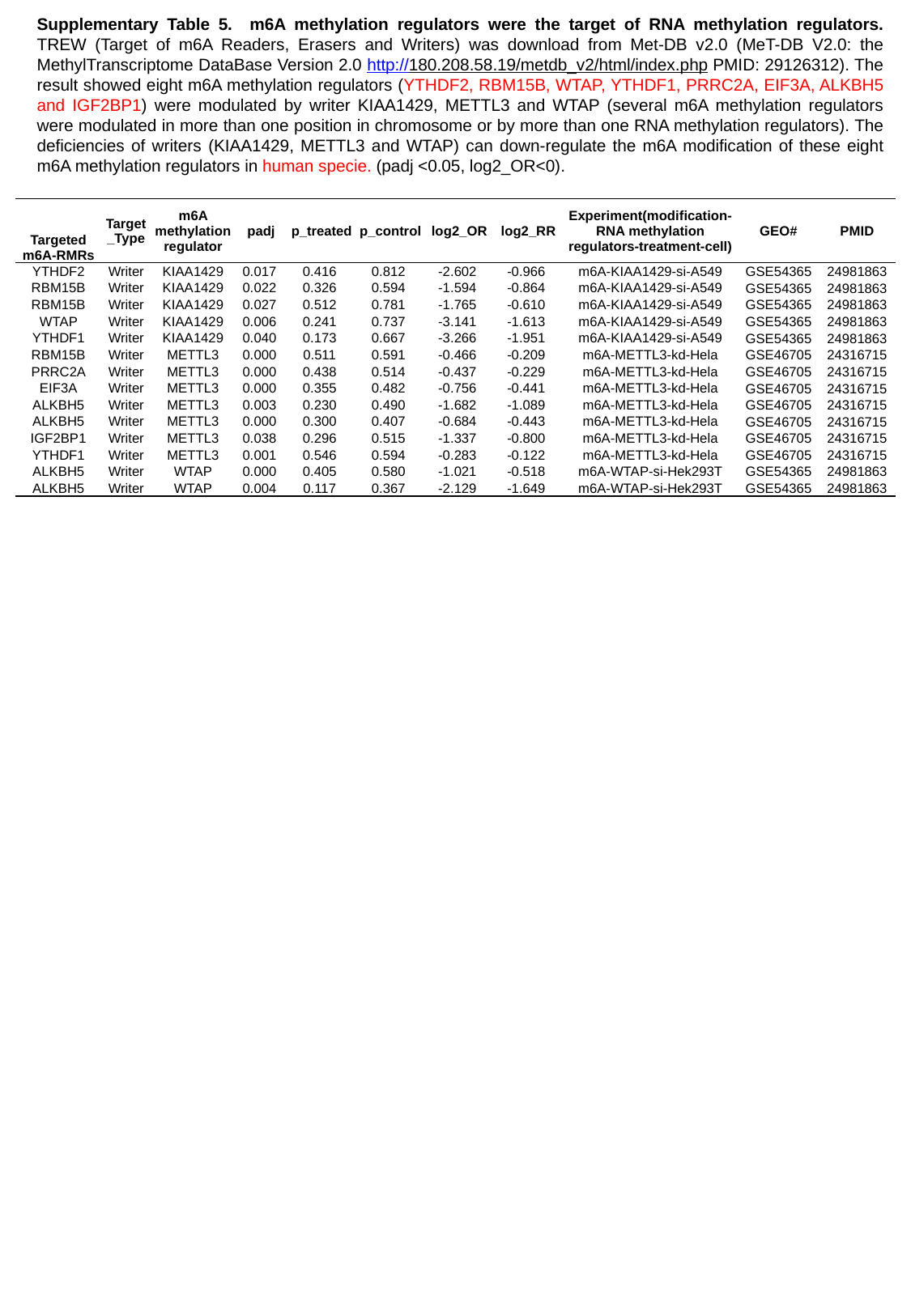

Supplementary Table 5. m6A methylation regulators were the target of RNA methylation regulators. TREW (Target of m6A Readers, Erasers and Writers) was download from Met-DB v2.0 (MeT-DB V2.0: the MethylTranscriptome DataBase Version 2.0 http://180.208.58.19/metdb_v2/html/index.php PMID: 29126312). The result showed eight m6A methylation regulators (YTHDF2, RBM15B, WTAP, YTHDF1, PRRC2A, EIF3A, ALKBH5 and IGF2BP1) were modulated by writer KIAA1429, METTL3 and WTAP (several m6A methylation regulators were modulated in more than one position in chromosome or by more than one RNA methylation regulators). The deficiencies of writers (KIAA1429, METTL3 and WTAP) can down-regulate the m6A modification of these eight m6A methylation regulators in human specie. (padj <0.05, log2_OR<0).
| Targeted m6A-RMRs | Target\_Type | m6A methylation regulator | padj | p\_treated | p\_control | log2\_OR | log2\_RR | Experiment(modification-RNA methylation regulators-treatment-cell) | GEO# | PMID |
| --- | --- | --- | --- | --- | --- | --- | --- | --- | --- | --- |
| YTHDF2 | Writer | KIAA1429 | 0.017 | 0.416 | 0.812 | -2.602 | -0.966 | m6A-KIAA1429-si-A549 | GSE54365 | 24981863 |
| RBM15B | Writer | KIAA1429 | 0.022 | 0.326 | 0.594 | -1.594 | -0.864 | m6A-KIAA1429-si-A549 | GSE54365 | 24981863 |
| RBM15B | Writer | KIAA1429 | 0.027 | 0.512 | 0.781 | -1.765 | -0.610 | m6A-KIAA1429-si-A549 | GSE54365 | 24981863 |
| WTAP | Writer | KIAA1429 | 0.006 | 0.241 | 0.737 | -3.141 | -1.613 | m6A-KIAA1429-si-A549 | GSE54365 | 24981863 |
| YTHDF1 | Writer | KIAA1429 | 0.040 | 0.173 | 0.667 | -3.266 | -1.951 | m6A-KIAA1429-si-A549 | GSE54365 | 24981863 |
| RBM15B | Writer | METTL3 | 0.000 | 0.511 | 0.591 | -0.466 | -0.209 | m6A-METTL3-kd-Hela | GSE46705 | 24316715 |
| PRRC2A | Writer | METTL3 | 0.000 | 0.438 | 0.514 | -0.437 | -0.229 | m6A-METTL3-kd-Hela | GSE46705 | 24316715 |
| EIF3A | Writer | METTL3 | 0.000 | 0.355 | 0.482 | -0.756 | -0.441 | m6A-METTL3-kd-Hela | GSE46705 | 24316715 |
| ALKBH5 | Writer | METTL3 | 0.003 | 0.230 | 0.490 | -1.682 | -1.089 | m6A-METTL3-kd-Hela | GSE46705 | 24316715 |
| ALKBH5 | Writer | METTL3 | 0.000 | 0.300 | 0.407 | -0.684 | -0.443 | m6A-METTL3-kd-Hela | GSE46705 | 24316715 |
| IGF2BP1 | Writer | METTL3 | 0.038 | 0.296 | 0.515 | -1.337 | -0.800 | m6A-METTL3-kd-Hela | GSE46705 | 24316715 |
| YTHDF1 | Writer | METTL3 | 0.001 | 0.546 | 0.594 | -0.283 | -0.122 | m6A-METTL3-kd-Hela | GSE46705 | 24316715 |
| ALKBH5 | Writer | WTAP | 0.000 | 0.405 | 0.580 | -1.021 | -0.518 | m6A-WTAP-si-Hek293T | GSE54365 | 24981863 |
| ALKBH5 | Writer | WTAP | 0.004 | 0.117 | 0.367 | -2.129 | -1.649 | m6A-WTAP-si-Hek293T | GSE54365 | 24981863 |

## Slide 8
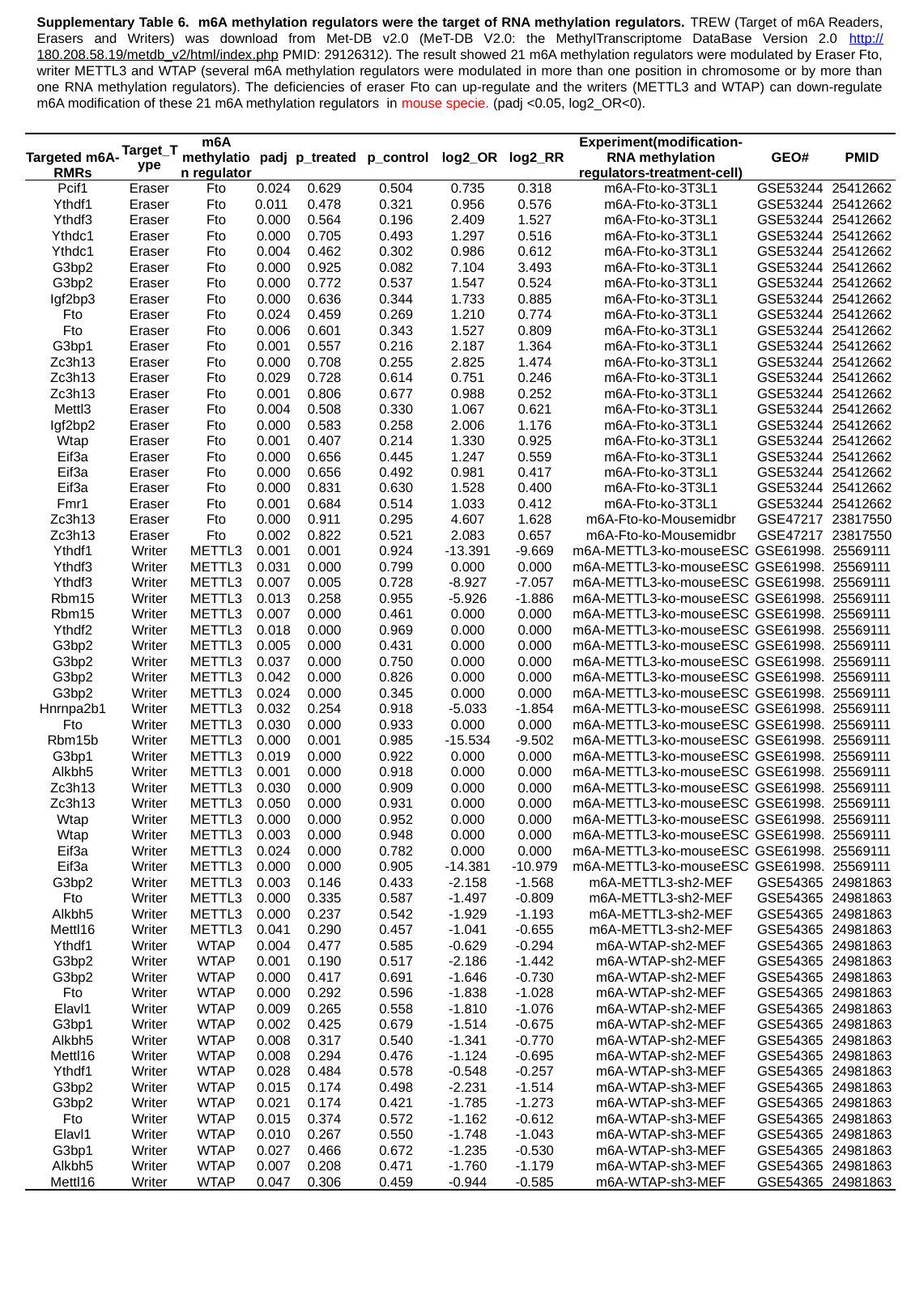

Supplementary Table 6. m6A methylation regulators were the target of RNA methylation regulators. TREW (Target of m6A Readers, Erasers and Writers) was download from Met-DB v2.0 (MeT-DB V2.0: the MethylTranscriptome DataBase Version 2.0 http://180.208.58.19/metdb_v2/html/index.php PMID: 29126312). The result showed 21 m6A methylation regulators were modulated by Eraser Fto, writer METTL3 and WTAP (several m6A methylation regulators were modulated in more than one position in chromosome or by more than one RNA methylation regulators). The deficiencies of eraser Fto can up-regulate and the writers (METTL3 and WTAP) can down-regulate m6A modification of these 21 m6A methylation regulators in mouse specie. (padj <0.05, log2_OR<0).
| Targeted m6A-RMRs | Target\_Type | m6A methylation regulator | padj | p\_treated | p\_control | log2\_OR | log2\_RR | Experiment(modification-RNA methylation regulators-treatment-cell) | GEO# | PMID |
| --- | --- | --- | --- | --- | --- | --- | --- | --- | --- | --- |
| Pcif1 | Eraser | Fto | 0.024 | 0.629 | 0.504 | 0.735 | 0.318 | m6A-Fto-ko-3T3L1 | GSE53244 | 25412662 |
| Ythdf1 | Eraser | Fto | 0.011 | 0.478 | 0.321 | 0.956 | 0.576 | m6A-Fto-ko-3T3L1 | GSE53244 | 25412662 |
| Ythdf3 | Eraser | Fto | 0.000 | 0.564 | 0.196 | 2.409 | 1.527 | m6A-Fto-ko-3T3L1 | GSE53244 | 25412662 |
| Ythdc1 | Eraser | Fto | 0.000 | 0.705 | 0.493 | 1.297 | 0.516 | m6A-Fto-ko-3T3L1 | GSE53244 | 25412662 |
| Ythdc1 | Eraser | Fto | 0.004 | 0.462 | 0.302 | 0.986 | 0.612 | m6A-Fto-ko-3T3L1 | GSE53244 | 25412662 |
| G3bp2 | Eraser | Fto | 0.000 | 0.925 | 0.082 | 7.104 | 3.493 | m6A-Fto-ko-3T3L1 | GSE53244 | 25412662 |
| G3bp2 | Eraser | Fto | 0.000 | 0.772 | 0.537 | 1.547 | 0.524 | m6A-Fto-ko-3T3L1 | GSE53244 | 25412662 |
| Igf2bp3 | Eraser | Fto | 0.000 | 0.636 | 0.344 | 1.733 | 0.885 | m6A-Fto-ko-3T3L1 | GSE53244 | 25412662 |
| Fto | Eraser | Fto | 0.024 | 0.459 | 0.269 | 1.210 | 0.774 | m6A-Fto-ko-3T3L1 | GSE53244 | 25412662 |
| Fto | Eraser | Fto | 0.006 | 0.601 | 0.343 | 1.527 | 0.809 | m6A-Fto-ko-3T3L1 | GSE53244 | 25412662 |
| G3bp1 | Eraser | Fto | 0.001 | 0.557 | 0.216 | 2.187 | 1.364 | m6A-Fto-ko-3T3L1 | GSE53244 | 25412662 |
| Zc3h13 | Eraser | Fto | 0.000 | 0.708 | 0.255 | 2.825 | 1.474 | m6A-Fto-ko-3T3L1 | GSE53244 | 25412662 |
| Zc3h13 | Eraser | Fto | 0.029 | 0.728 | 0.614 | 0.751 | 0.246 | m6A-Fto-ko-3T3L1 | GSE53244 | 25412662 |
| Zc3h13 | Eraser | Fto | 0.001 | 0.806 | 0.677 | 0.988 | 0.252 | m6A-Fto-ko-3T3L1 | GSE53244 | 25412662 |
| Mettl3 | Eraser | Fto | 0.004 | 0.508 | 0.330 | 1.067 | 0.621 | m6A-Fto-ko-3T3L1 | GSE53244 | 25412662 |
| Igf2bp2 | Eraser | Fto | 0.000 | 0.583 | 0.258 | 2.006 | 1.176 | m6A-Fto-ko-3T3L1 | GSE53244 | 25412662 |
| Wtap | Eraser | Fto | 0.001 | 0.407 | 0.214 | 1.330 | 0.925 | m6A-Fto-ko-3T3L1 | GSE53244 | 25412662 |
| Eif3a | Eraser | Fto | 0.000 | 0.656 | 0.445 | 1.247 | 0.559 | m6A-Fto-ko-3T3L1 | GSE53244 | 25412662 |
| Eif3a | Eraser | Fto | 0.000 | 0.656 | 0.492 | 0.981 | 0.417 | m6A-Fto-ko-3T3L1 | GSE53244 | 25412662 |
| Eif3a | Eraser | Fto | 0.000 | 0.831 | 0.630 | 1.528 | 0.400 | m6A-Fto-ko-3T3L1 | GSE53244 | 25412662 |
| Fmr1 | Eraser | Fto | 0.001 | 0.684 | 0.514 | 1.033 | 0.412 | m6A-Fto-ko-3T3L1 | GSE53244 | 25412662 |
| Zc3h13 | Eraser | Fto | 0.000 | 0.911 | 0.295 | 4.607 | 1.628 | m6A-Fto-ko-Mousemidbr | GSE47217 | 23817550 |
| Zc3h13 | Eraser | Fto | 0.002 | 0.822 | 0.521 | 2.083 | 0.657 | m6A-Fto-ko-Mousemidbr | GSE47217 | 23817550 |
| Ythdf1 | Writer | METTL3 | 0.001 | 0.001 | 0.924 | -13.391 | -9.669 | m6A-METTL3-ko-mouseESC | GSE61998. | 25569111 |
| Ythdf3 | Writer | METTL3 | 0.031 | 0.000 | 0.799 | 0.000 | 0.000 | m6A-METTL3-ko-mouseESC | GSE61998. | 25569111 |
| Ythdf3 | Writer | METTL3 | 0.007 | 0.005 | 0.728 | -8.927 | -7.057 | m6A-METTL3-ko-mouseESC | GSE61998. | 25569111 |
| Rbm15 | Writer | METTL3 | 0.013 | 0.258 | 0.955 | -5.926 | -1.886 | m6A-METTL3-ko-mouseESC | GSE61998. | 25569111 |
| Rbm15 | Writer | METTL3 | 0.007 | 0.000 | 0.461 | 0.000 | 0.000 | m6A-METTL3-ko-mouseESC | GSE61998. | 25569111 |
| Ythdf2 | Writer | METTL3 | 0.018 | 0.000 | 0.969 | 0.000 | 0.000 | m6A-METTL3-ko-mouseESC | GSE61998. | 25569111 |
| G3bp2 | Writer | METTL3 | 0.005 | 0.000 | 0.431 | 0.000 | 0.000 | m6A-METTL3-ko-mouseESC | GSE61998. | 25569111 |
| G3bp2 | Writer | METTL3 | 0.037 | 0.000 | 0.750 | 0.000 | 0.000 | m6A-METTL3-ko-mouseESC | GSE61998. | 25569111 |
| G3bp2 | Writer | METTL3 | 0.042 | 0.000 | 0.826 | 0.000 | 0.000 | m6A-METTL3-ko-mouseESC | GSE61998. | 25569111 |
| G3bp2 | Writer | METTL3 | 0.024 | 0.000 | 0.345 | 0.000 | 0.000 | m6A-METTL3-ko-mouseESC | GSE61998. | 25569111 |
| Hnrnpa2b1 | Writer | METTL3 | 0.032 | 0.254 | 0.918 | -5.033 | -1.854 | m6A-METTL3-ko-mouseESC | GSE61998. | 25569111 |
| Fto | Writer | METTL3 | 0.030 | 0.000 | 0.933 | 0.000 | 0.000 | m6A-METTL3-ko-mouseESC | GSE61998. | 25569111 |
| Rbm15b | Writer | METTL3 | 0.000 | 0.001 | 0.985 | -15.534 | -9.502 | m6A-METTL3-ko-mouseESC | GSE61998. | 25569111 |
| G3bp1 | Writer | METTL3 | 0.019 | 0.000 | 0.922 | 0.000 | 0.000 | m6A-METTL3-ko-mouseESC | GSE61998. | 25569111 |
| Alkbh5 | Writer | METTL3 | 0.001 | 0.000 | 0.918 | 0.000 | 0.000 | m6A-METTL3-ko-mouseESC | GSE61998. | 25569111 |
| Zc3h13 | Writer | METTL3 | 0.030 | 0.000 | 0.909 | 0.000 | 0.000 | m6A-METTL3-ko-mouseESC | GSE61998. | 25569111 |
| Zc3h13 | Writer | METTL3 | 0.050 | 0.000 | 0.931 | 0.000 | 0.000 | m6A-METTL3-ko-mouseESC | GSE61998. | 25569111 |
| Wtap | Writer | METTL3 | 0.000 | 0.000 | 0.952 | 0.000 | 0.000 | m6A-METTL3-ko-mouseESC | GSE61998. | 25569111 |
| Wtap | Writer | METTL3 | 0.003 | 0.000 | 0.948 | 0.000 | 0.000 | m6A-METTL3-ko-mouseESC | GSE61998. | 25569111 |
| Eif3a | Writer | METTL3 | 0.024 | 0.000 | 0.782 | 0.000 | 0.000 | m6A-METTL3-ko-mouseESC | GSE61998. | 25569111 |
| Eif3a | Writer | METTL3 | 0.000 | 0.000 | 0.905 | -14.381 | -10.979 | m6A-METTL3-ko-mouseESC | GSE61998. | 25569111 |
| G3bp2 | Writer | METTL3 | 0.003 | 0.146 | 0.433 | -2.158 | -1.568 | m6A-METTL3-sh2-MEF | GSE54365 | 24981863 |
| Fto | Writer | METTL3 | 0.000 | 0.335 | 0.587 | -1.497 | -0.809 | m6A-METTL3-sh2-MEF | GSE54365 | 24981863 |
| Alkbh5 | Writer | METTL3 | 0.000 | 0.237 | 0.542 | -1.929 | -1.193 | m6A-METTL3-sh2-MEF | GSE54365 | 24981863 |
| Mettl16 | Writer | METTL3 | 0.041 | 0.290 | 0.457 | -1.041 | -0.655 | m6A-METTL3-sh2-MEF | GSE54365 | 24981863 |
| Ythdf1 | Writer | WTAP | 0.004 | 0.477 | 0.585 | -0.629 | -0.294 | m6A-WTAP-sh2-MEF | GSE54365 | 24981863 |
| G3bp2 | Writer | WTAP | 0.001 | 0.190 | 0.517 | -2.186 | -1.442 | m6A-WTAP-sh2-MEF | GSE54365 | 24981863 |
| G3bp2 | Writer | WTAP | 0.000 | 0.417 | 0.691 | -1.646 | -0.730 | m6A-WTAP-sh2-MEF | GSE54365 | 24981863 |
| Fto | Writer | WTAP | 0.000 | 0.292 | 0.596 | -1.838 | -1.028 | m6A-WTAP-sh2-MEF | GSE54365 | 24981863 |
| Elavl1 | Writer | WTAP | 0.009 | 0.265 | 0.558 | -1.810 | -1.076 | m6A-WTAP-sh2-MEF | GSE54365 | 24981863 |
| G3bp1 | Writer | WTAP | 0.002 | 0.425 | 0.679 | -1.514 | -0.675 | m6A-WTAP-sh2-MEF | GSE54365 | 24981863 |
| Alkbh5 | Writer | WTAP | 0.008 | 0.317 | 0.540 | -1.341 | -0.770 | m6A-WTAP-sh2-MEF | GSE54365 | 24981863 |
| Mettl16 | Writer | WTAP | 0.008 | 0.294 | 0.476 | -1.124 | -0.695 | m6A-WTAP-sh2-MEF | GSE54365 | 24981863 |
| Ythdf1 | Writer | WTAP | 0.028 | 0.484 | 0.578 | -0.548 | -0.257 | m6A-WTAP-sh3-MEF | GSE54365 | 24981863 |
| G3bp2 | Writer | WTAP | 0.015 | 0.174 | 0.498 | -2.231 | -1.514 | m6A-WTAP-sh3-MEF | GSE54365 | 24981863 |
| G3bp2 | Writer | WTAP | 0.021 | 0.174 | 0.421 | -1.785 | -1.273 | m6A-WTAP-sh3-MEF | GSE54365 | 24981863 |
| Fto | Writer | WTAP | 0.015 | 0.374 | 0.572 | -1.162 | -0.612 | m6A-WTAP-sh3-MEF | GSE54365 | 24981863 |
| Elavl1 | Writer | WTAP | 0.010 | 0.267 | 0.550 | -1.748 | -1.043 | m6A-WTAP-sh3-MEF | GSE54365 | 24981863 |
| G3bp1 | Writer | WTAP | 0.027 | 0.466 | 0.672 | -1.235 | -0.530 | m6A-WTAP-sh3-MEF | GSE54365 | 24981863 |
| Alkbh5 | Writer | WTAP | 0.007 | 0.208 | 0.471 | -1.760 | -1.179 | m6A-WTAP-sh3-MEF | GSE54365 | 24981863 |
| Mettl16 | Writer | WTAP | 0.047 | 0.306 | 0.459 | -0.944 | -0.585 | m6A-WTAP-sh3-MEF | GSE54365 | 24981863 |

## Slide 9
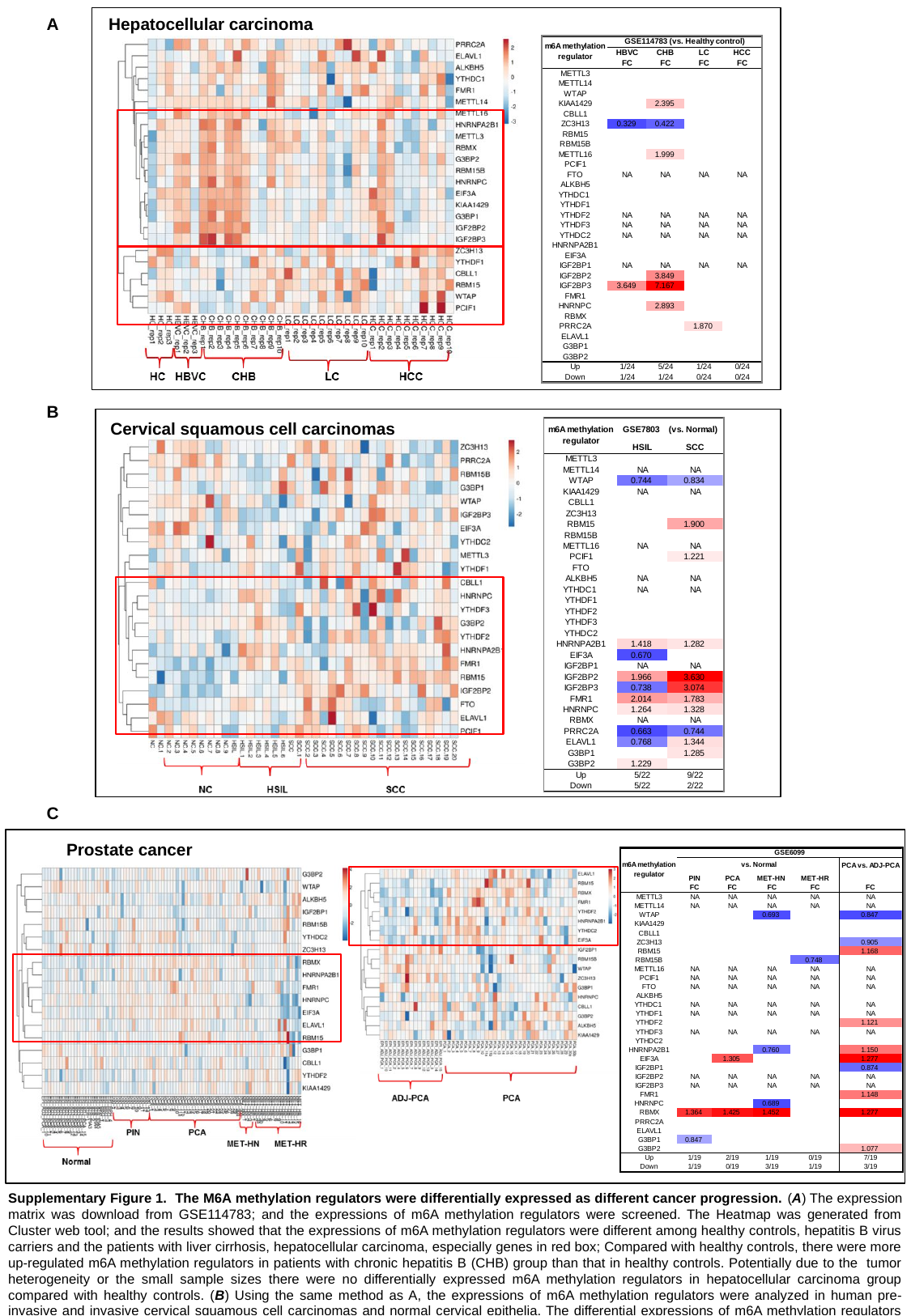

A
Hepatocellular carcinoma
B
Cervical squamous cell carcinomas
C
Prostate cancer
Supplementary Figure 1. The M6A methylation regulators were differentially expressed as different cancer progression. (A) The expression matrix was download from GSE114783; and the expressions of m6A methylation regulators were screened. The Heatmap was generated from Cluster web tool; and the results showed that the expressions of m6A methylation regulators were different among healthy controls, hepatitis B virus carriers and the patients with liver cirrhosis, hepatocellular carcinoma, especially genes in red box; Compared with healthy controls, there were more up-regulated m6A methylation regulators in patients with chronic hepatitis B (CHB) group than that in healthy controls. Potentially due to the tumor heterogeneity or the small sample sizes there were no differentially expressed m6A methylation regulators in hepatocellular carcinoma group compared with healthy controls. (B) Using the same method as A, the expressions of m6A methylation regulators were analyzed in human pre-invasive and invasive cervical squamous cell carcinomas and normal cervical epithelia. The differential expressions of m6A methylation regulators were shown in red box. The 22 m6A methylation regulators were examined in these three groups. The results showed that the expressions of WTAP were down-regulated; and the expressions of HNRNPA2B1, IGF2BP2, FMR1 and HNRNPC were up-regulated in patients with pre-invasive and invasive cervical squamous cell carcinomas compared with normal controls. (C) the expressions of m6A methylation regulators were analyzed in prostate cancer from benign prostatic hyperplasia to metastatic prostate cancer. The expression of RBMX was gradually increased from prostatic intraepithelial neoplasia to hormone-naïve prostate cancer compared with normal samples. There were more obvious differential expressions of m6A methylation regulators between adjacent prostate cancer samples and prostate carcinoma samples. The expressions of EIF3A and RBMX were up-regulated in prostate carcinoma samples compared with normal prostate epithelium-adjacent Samples.
Abbreviations: HC, healthy control; HBVC, hepatitis B virus carrier; CHB, chronic hepatitis B; LC, liver cirrhosis; HCC, hepatocellular carcinoma; NC, Normal Cervix; HSIL, high grade squamous intraepithelial lesion of the cervix; SCC, squamous cell carcinoma of the cervix. Note EPI_ADJ_PCA, EPI_ATR and EPI_BPH groups were looked as normal samples reference to the original article (PMID: 17173048). EPI_ADJ_PCA, Normal Prostate Epithelium-Adjacent Sample; EPI_ATR, Atrophic Prostate Epithelium - Simple Cystic Atrophy Sample; EPI_BPH, BPH Prostate Epithelium - Epithelial BPH Sample (benign prostatic hyperplasia); PIN, prostatic intraepithelial neoplasia; PCA, Prostate Carcinoma; MET_HN, hormone-naïve prostate cancer; MET_HR, hormone-refractory metastatic prostate cancer.

## Slide 10
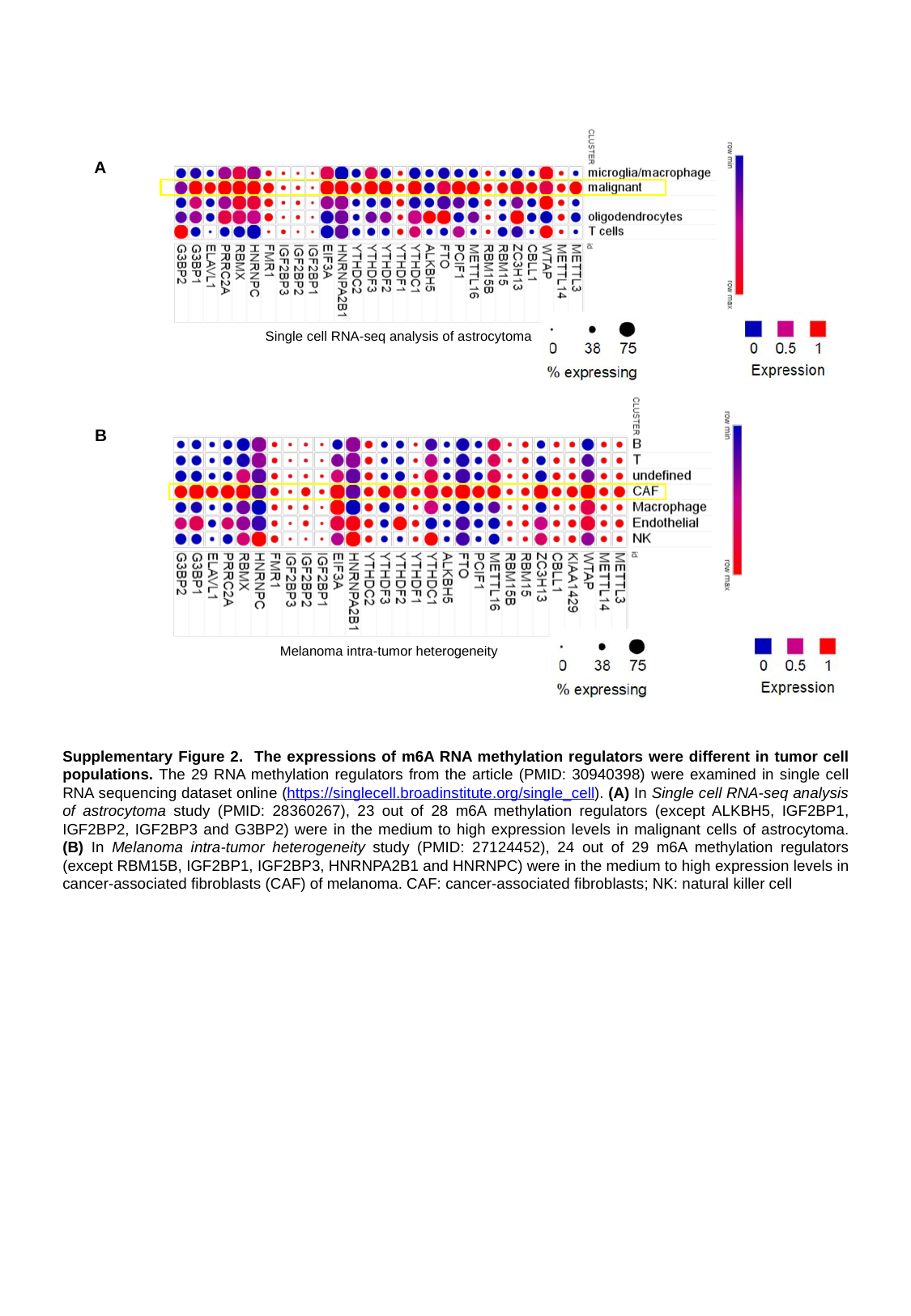

A
Single cell RNA-seq analysis of astrocytoma
B
Melanoma intra-tumor heterogeneity
Supplementary Figure 2. The expressions of m6A RNA methylation regulators were different in tumor cell populations. The 29 RNA methylation regulators from the article (PMID: 30940398) were examined in single cell RNA sequencing dataset online (https://singlecell.broadinstitute.org/single_cell). (A) In Single cell RNA-seq analysis of astrocytoma study (PMID: 28360267), 23 out of 28 m6A methylation regulators (except ALKBH5, IGF2BP1, IGF2BP2, IGF2BP3 and G3BP2) were in the medium to high expression levels in malignant cells of astrocytoma. (B) In Melanoma intra-tumor heterogeneity study (PMID: 27124452), 24 out of 29 m6A methylation regulators (except RBM15B, IGF2BP1, IGF2BP3, HNRNPA2B1 and HNRNPC) were in the medium to high expression levels in cancer-associated fibroblasts (CAF) of melanoma. CAF: cancer-associated fibroblasts; NK: natural killer cell

## Slide 11
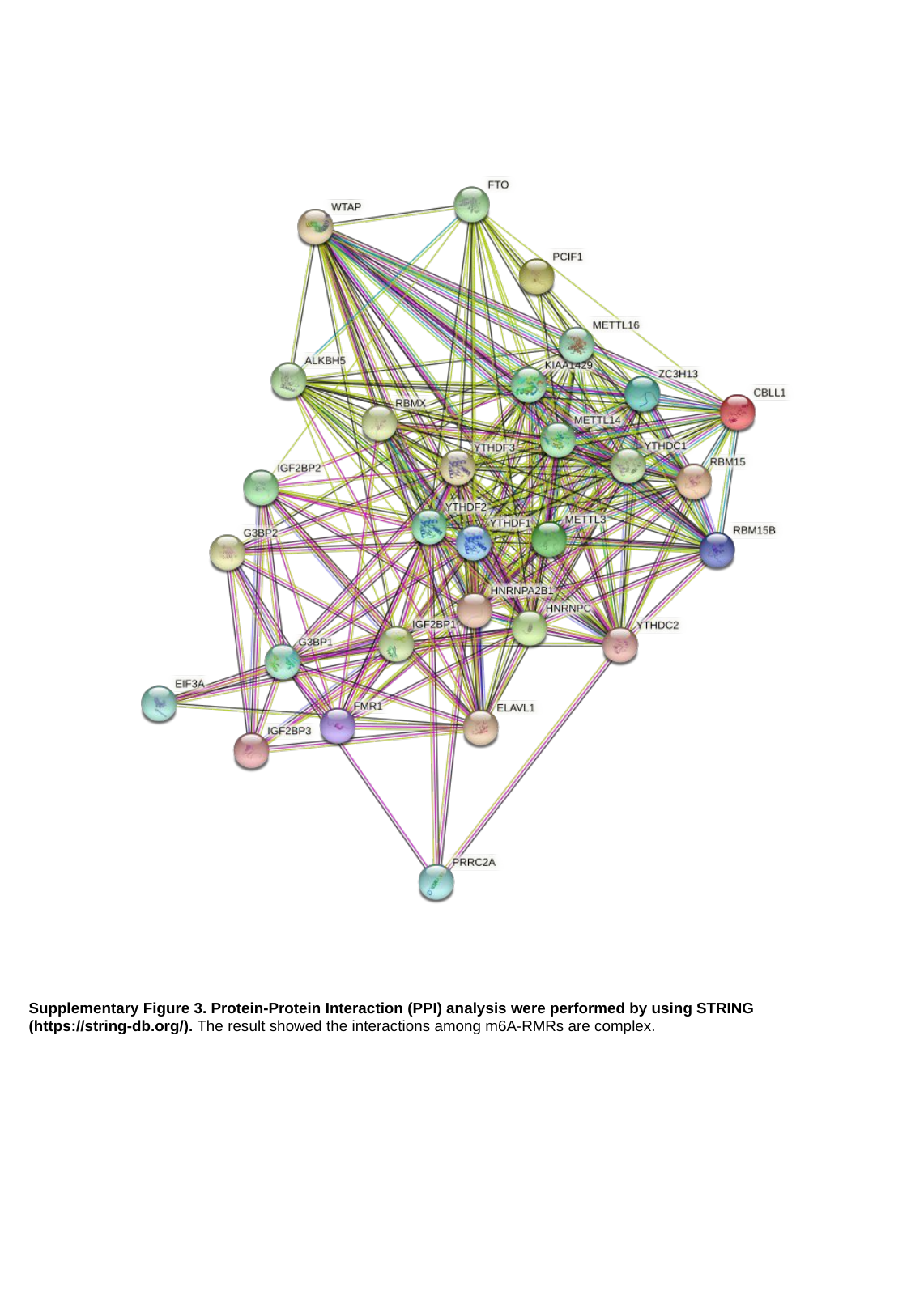

Supplementary Figure 3. Protein-Protein Interaction (PPI) analysis were performed by using STRING (https://string-db.org/). The result showed the interactions among m6A-RMRs are complex.

## Slide 12
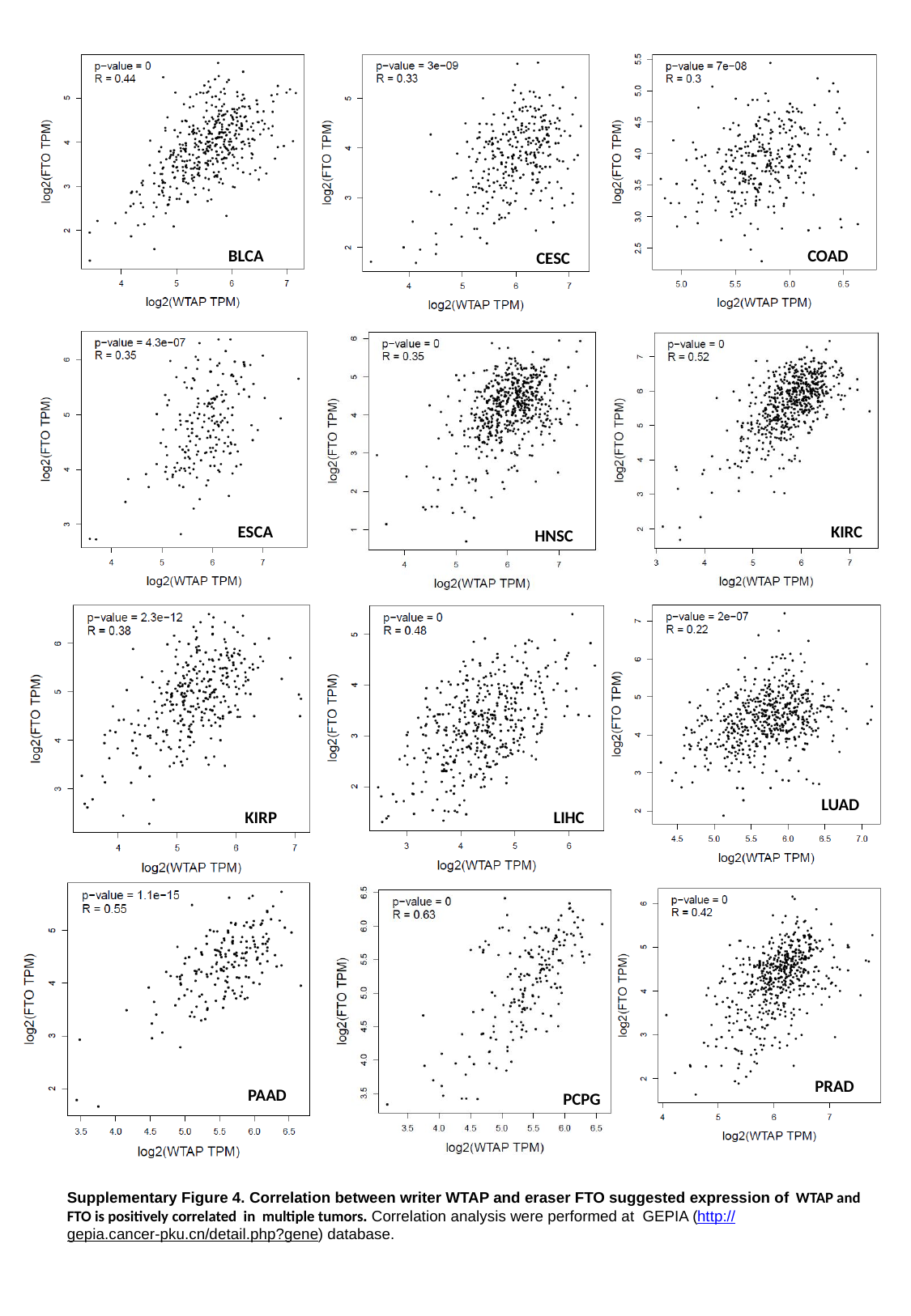

COAD
BLCA
CESC
KIRC
ESCA
HNSC
LUAD
LIHC
KIRP
PRAD
PAAD
PCPG
Supplementary Figure 4. Correlation between writer WTAP and eraser FTO suggested expression of WTAP and FTO is positively correlated in multiple tumors. Correlation analysis were performed at GEPIA (http://gepia.cancer-pku.cn/detail.php?gene) database.

## Slide 13
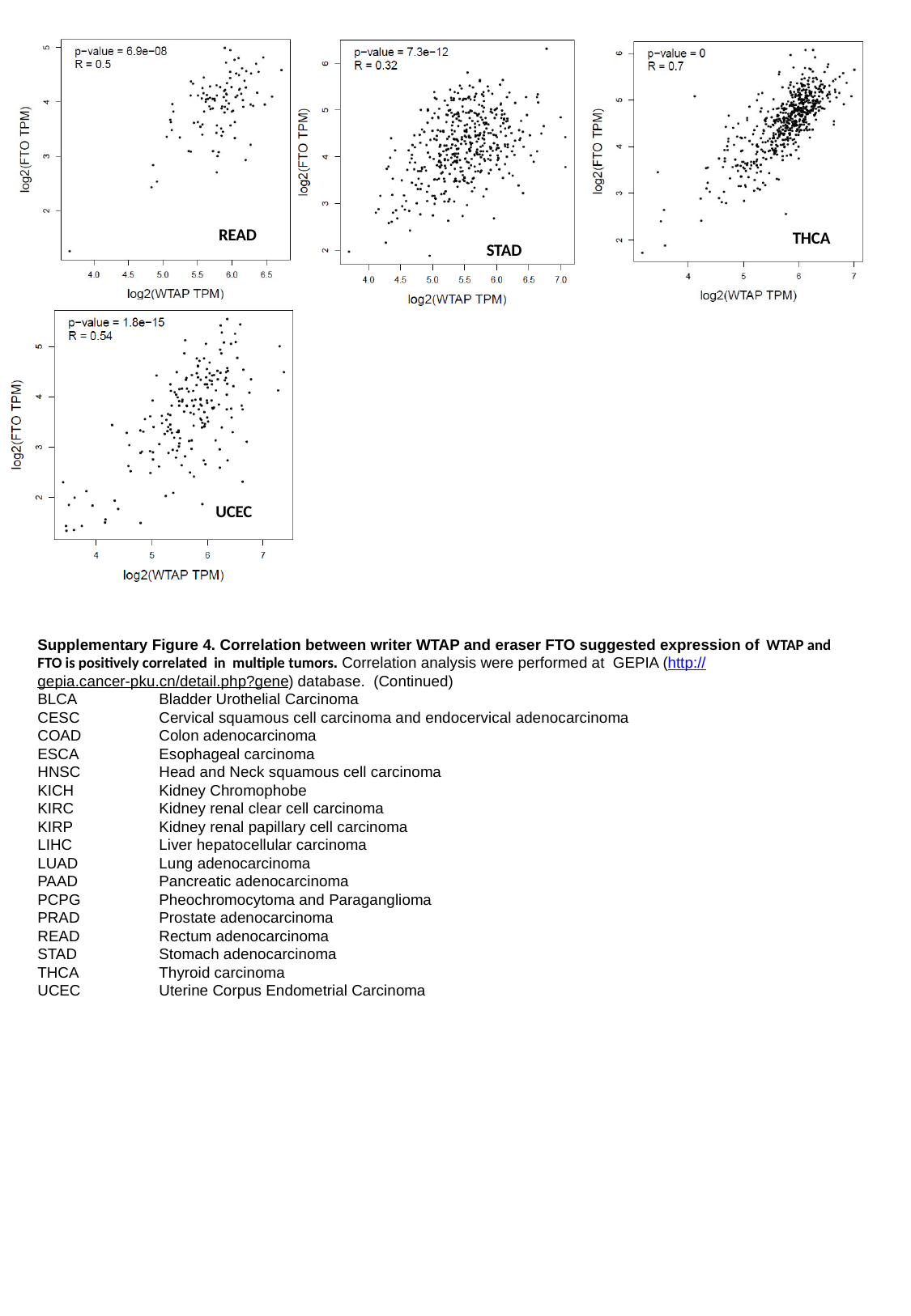

READ
THCA
STAD
UCEC
Supplementary Figure 4. Correlation between writer WTAP and eraser FTO suggested expression of WTAP and FTO is positively correlated in multiple tumors. Correlation analysis were performed at GEPIA (http://gepia.cancer-pku.cn/detail.php?gene) database. (Continued)
BLCA	Bladder Urothelial Carcinoma
CESC	Cervical squamous cell carcinoma and endocervical adenocarcinoma
COAD	Colon adenocarcinoma
ESCA	Esophageal carcinoma
HNSC	Head and Neck squamous cell carcinoma
KICH	Kidney Chromophobe
KIRC	Kidney renal clear cell carcinoma
KIRP	Kidney renal papillary cell carcinoma
LIHC	Liver hepatocellular carcinoma
LUAD	Lung adenocarcinoma
PAAD	Pancreatic adenocarcinoma
PCPG	Pheochromocytoma and Paraganglioma
PRAD	Prostate adenocarcinoma
READ	Rectum adenocarcinoma
STAD	Stomach adenocarcinoma
THCA	Thyroid carcinoma
UCEC	Uterine Corpus Endometrial Carcinoma
